# Inequality in human development amplifies climate-related disaster risk

**DOI:** 10.1038/s41467-026-73873-9

**Published:** 2026-06-17

**Authors:** Khalil Teber, Sebastian Sippel, Melanie Krause, Jakob Zscheischler, Miguel D. Mahecha

**Affiliations:** 1 Institute for Earth System Science and Remote Sensing, Leipzig University, Leipzig, Germany; 2 Leipzig Institute for Meteorology, Leipzig University, Leipzig, Germany; 3 Faculty for Economics and Management Sciences, Leipzig University, Leipzig, Germany; 4 Helmholtz Centre for Environmental Research—UFZ, Leipzig, Germany; 5 Department of Hydro Sciences, TUD Dresden University of Technology, Dresden, Germany; 6 Center for Scalable Data Analytics and Artificial Intelligence (ScaDS.AI), Dresden/Leipzig, Germany

**Keywords:** Natural hazards, Interdisciplinary studies, Climate-change impacts

## Abstract

The impacts of climate-related disasters are shaped by the interaction between hazard intensity, exposure, and vulnerability. However, the influence of hazard intensity and within-country inequality on impact magnitudes remains poorly quantified. Here, we present a global multi-hazard study of over 7000 climate-related disasters reported by the Emergency Events Database from 1990 to 2020. Using subnational indicators, we show that human development drives major shifts in global exposure and impact patterns, with societal vulnerability outweighing hazard intensity in shaping impacts. Despite a declining share of global exposure over the past three decades, regions with low subnational Human Development Index scores experience disproportionately higher human losses across most disaster types. For instance, individuals in these regions face an 8.2-fold higher risk of fatality associated with storms (95% confidence interval: 2.16-23.06) compared to those in very high human development regions. Our findings also indicate that within-country inequality in human development exacerbates disaster risk in regions with low and medium levels of human development. These results underscore the critical role of human development in managing disaster risks and highlight the link between socioeconomic conditions and vulnerability to climate-related hazards.

## Abstract (web summary)

A global analysis of over 7,000 climate-related disasters shows that human development is a stronger determinant of local disaster impacts than hazard intensity, and that inequality in human development amplifies risk in the least developed regions.

## Introduction

Climate-related disasters can cause loss of life, disease outbreaks, and medical emergencies^1^. They also damage infrastructure^2^; devastate economies^3^; ruin crops^4^; diminish economic growth^5^; and may destabilize governments^6,7^. In 2023 alone, the Emergency Events Database (EM-DAT) recorded 399 climate-related disasters affecting 93.1 million people, causing over 86,000 fatalities, and resulting in a total of US$202.7 billion in reported economic losses^8^. Climate extremes are expected to increase in both intensity and frequency^9^, which raises the question of how the associated disasters will impact societies differently, given their relative level of socioeconomic development.

The risk of disaster arising from a hazard is a function of natural and social factors^10,11^. Climate hazards constitute the physical elements of risk, while societal aspects are represented by population and economic exposure, socioeconomic vulnerability, and response or adaptive capacity. Higher levels of economic and human development generally reduce the risk of adverse impacts from climate-related hazards^12–14^, and the associated fatality tolls are typically lower^15^. Conversely, people in low-income or low human development countries typically experience more severe impacts from climate-related hazards, such as floods^16–19^ and tropical cyclones^20^.

Studies on disaster impacts that consider vulnerability, inequality, and hazard intensity typically rely on national-level indicators or country classifications^14,16–19,21^. However, climate-related hazards rarely impact entire countries; they more often affect specific regions, and their impacts depend on local geographic and human factors^22^. Using national-level indices for subnational events obscures within-country disparities, especially in large nations with internally diverse climates and socioeconomic development. Understanding how local socioeconomic conditions influence the extent to which people are impacted by climate-related hazards is essential^23^. People living in poverty face reduced access to resources, quality infrastructure, healthcare, and education^24^. These disparities are captured in the Human Development Index (HDI)^25^, a composite indicator that measures achievements in health, education, and income. Although the HDI is a commonly reported national statistic, differences in its underlying components (education, health, and income) within one country can be as large as differences between countries^26^. Recently, subnational Human Development Index (sHDI) data have become available^27^, enabling global-scale analyses based on regional estimates of socioeconomic vulnerability. Despite these advances, the integration of subnational socioeconomic heterogeneity with global multi-hazard disaster impact assessments remains in its infancy.

In this work, we quantify how inequalities in subnational human development influence the impacts of climate-related disasters at the global scale. We analyze a sample of over 7000 climate-related disaster events documented by EM-DAT^28^ from 1990 to 2020 across 154 countries. We show that lower levels of human development are associated with disproportionately higher human losses and elevated relative economic losses. We further demonstrate that this development-impact gradient persists across most disaster types and hazard intensity levels, and that within-country inequalities amplify disaster risk. These findings provide a global perspective on how inequalities in human development relate to climate-related disaster impacts.

## Results

**Fig. 1:**
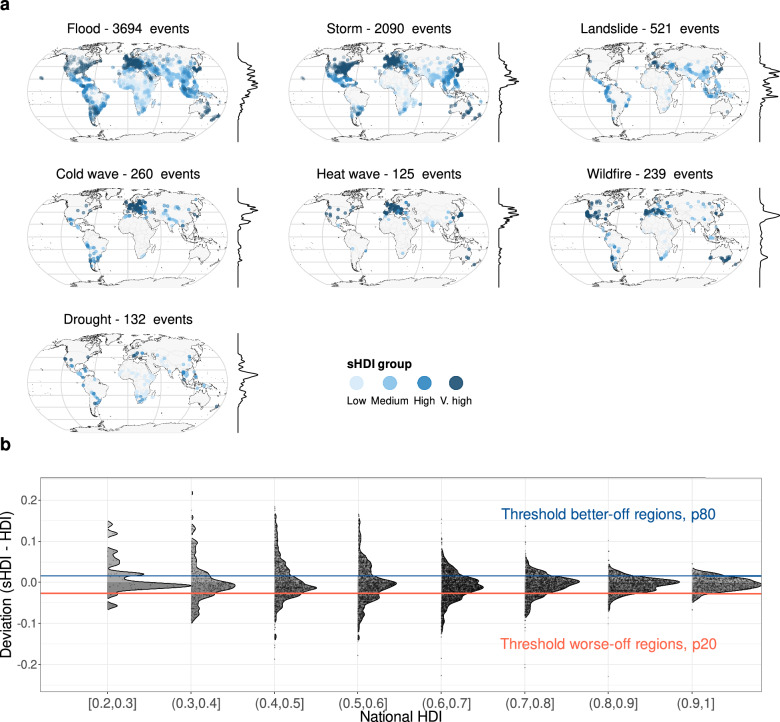
Climate-related disasters and intra-country deviation of human development in impacted regions. **a** Locations of climate-related disasters from the EM-DAT colored by sHDI group, shown separately for each disaster type between 1990 and 2020 (total *n* = 7061; per-type counts indicated in each panel). The four sHDI groups follow the United Nations Development Programme (UNDP) thresholds: low (sHDI < 0.55), medium (0.55 ≤ sHDI < 0.7), high (0.7 ≤ sHDI < 0.8), and very high (sHDI ≥ 0.8). Disaster counts by latitude are shown on the right of each map. Point density reflects only the number of events, and the apparent clustering results from spatial overlap of points, rather than representing any additional variable. Country boundaries and coastlines are from Natural Earth (naturalearthdata.com). Map projection: Robinson. **b** Difference between sHDI and national HDI for each impacted region, grouped by national HDI bin (*x*-axis). Horizontal lines indicate the thresholds used to define deviation groups: better-off regions (≥80th percentile, blue) and worse-off regions (≤20th, red); regions between the two thresholds are classified as national-average.

Our analysis of 7061 climate-related disaster events from 1990 to 2020 reveals that floods, storms, and landslides account for 89% of the reported events, causing human and economic losses across regions with diverse socioeconomic conditions on all continents (Fig. 1a and Supplementary Table 1). Disasters associated with cold waves, heat waves, and wildfires are predominantly reported in mid and high latitude regions that tend to exhibit high or very high sHDI. In contrast, drought-related disasters are more frequently reported in low-latitude regions, typically characterized by lower levels of development (low or medium sHDI; see Fig. 1a and Supplementary Table 2). The heterogeneous structure of the EM-DAT sample and the geographic variability of reported impacts are reflected in the number of events and in the mix of disaster types across countries (Supplementary Figs. 1 and 2).

Particularly in countries with low and medium national HDI, disaster-affected regions exhibit notable within-country variability in human development (Fig. 1b). In both groups (HDI < 0.7), worse-off regions (below the 20th percentile of sHDI deviation from the national HDI) and better-off regions (above the 80th percentile) are overrepresented in our sample. While 627 of 1840 low sHDI regions (34%) fall below the national HDI, 540 medium sHDI regions (24%) exceed it (Supplementary Table 3). In contrast, only 5.7% (*n* = 92) of the very high sHDI regions are below their national HDI, while 12.6% (*n* = 202) exceed it. Overall, 13.9% of disasters occurred in regions where the sHDI classification differed from the national HDI at the time of impact (Supplementary Fig. 3 and Supplementary Table 4). For example, out of 1347 events reported in countries with high HDI, 214 occurred in medium sHDI regions and 48 in very high sHDI regions.

**Fig. 2:**
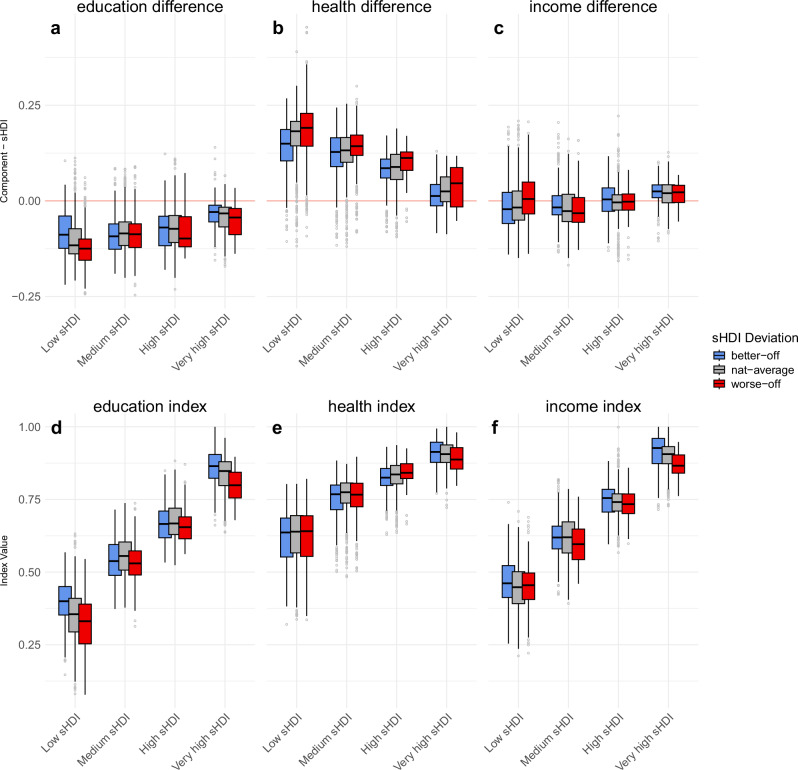
Components of sHDI by sHDI group and deviation from the national HDI. **a**–**c** Show the difference between each HDI component—education, health, and income, respectively - and the overall sHDI score (*y*-axis: Component − sHDI). The horizontal red line at zero indicates equal contribution of the component to sHDI. Positive values indicate an above-average contribution of the component; negative values indicate a below-average contribution. **d**–**f** Show the absolute index value of each component. In all panels, colors indicate the deviation from national HDI: better-off (≥ 80th percentile, blue), national-average (> 20th and < 80th percentile, gray; abbreviated to nat-average in the legend), and worse-off (≤ 20th, red). The box plots show the median (center line), interquartile range (box limits, 25th–75th percentiles), 1.5× interquartile range (whiskers), and outliers (individual points beyond the whiskers).

High and very high sHDI regions show balanced contributions of education, health and income to the composite sHDI (Fig. 2). By contrast, low sHDI regions display a pronounced imbalance, with the health index contributing more strongly than education or income (Fig. 2b). This pattern reflects the fact that health outcomes can improve relatively quickly through the diffusion of medical knowledge and targeted public health interventions, whereas progress in education and income requires long-term institutional capacity and structural transformation^29^. Because the sHDI is a geometric mean, weak performance in education and income substantially constrains overall development. Similar imbalances are also observed within comparable development levels (regions in the same sHDI group and even within the same country). Worse-off regions consistently lag behind in education and income, making health the dominant driver of their sHDI (Fig. 2e).

### Development drives major shifts in global exposure and impact patterns

**Fig. 3:**
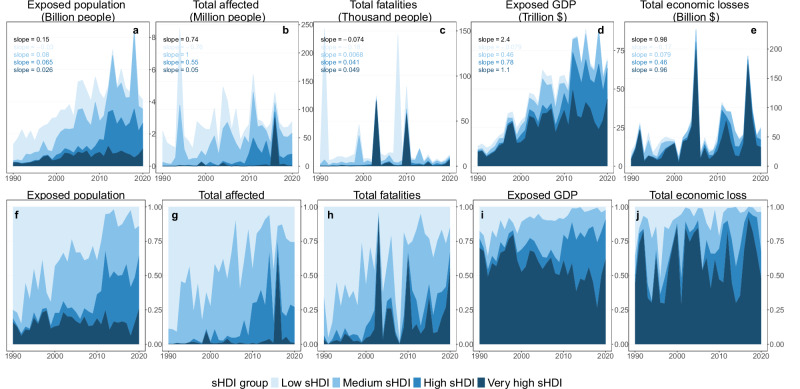
Temporal evolution of exposure and disaster impacts from 1990 to 2020 by sHDI group. **a**–**e** Show annual sums of exposed population (billion people), affected people (million people), fatalities (thousand people), exposed GDP (trillion US dollars), and total economic losses (billion US dollars) as stacked area charts, with shades of blue indicating sHDI from lightest (low) to darkest (very high). Median trends are estimated using quantile regression (*τ* = 0.5, the 0.5 quantile) for the global aggregate and for each sHDI group separately; the five corresponding slopes per panel are indicated in the figure, and *p*-values are provided in Supplementary Table 6. **f**–**j** Show the proportional share of each sHDI group of the corresponding exposure and impact variable (*y*-axis, 0–1).

Between 1990 and 2020, the majority of population exposure (64.7%) and human losses (83.1% of people affected and 75.3% of fatalities) were reported in regions with low and medium sHDI (Supplementary Table 5). In contrast, 76.6% economic exposure, measured as gross domestic product (GDP), and 78.4% of total economic losses were concentrated in regions with high or very high sHDI (Fig. 3 and Supplementary Table 6). Annual population exposure increased significantly over the same period (*p* < 0.05) by an average of 150 million people per year, while economic exposure rose by US$2.4 trillion per year (2011 prices; Fig. 3a, e). Despite rising population exposure, there was no corresponding increase in human losses. However, economic losses increased by an average of US$0.98 billion per year (Fig. 3b, c, e). Floods and storms accounted for the majority of the estimated exposure (population 84.6%, economic 81.6%) and reported losses (people affected 87.2%, fatalities 71.2%, economic losses 90%; see Supplementary Fig. 4 and Supplementary Table 7). The observed increase in exposed population and GDP may also reflect underlying demographic and economic growth rather than a direct rise in disaster risk^30^. This highlights the need to consider relative impact rates (e.g., fatalities per exposed population or economic losses per exposed GDP), which better capture disaster impacts in relation to the scale of exposure. Moreover, as regions and countries change sHDI groups over time, the composition and resilience of their exposed populations and assets also change. Tracking both relative impact metrics and shifts in sHDI groups is therefore essential for understanding the implications of evolving exposure to climate-related disasters.

Exposure and impacts are unevenly distributed across the different sHDI groups. As countries and regions achieve higher human development levels, the socioeconomic composition of the exposed populations changes. In 1990, low sHDI regions accounted for the highest share of global population exposure and human losses: over 75% of the exposed population, 88% of people affected, and 65% of fatalities (Fig. 3f–h). By 2020, these values decreased to 14% of the exposed population, 25% of people affected, and 15% of fatalities (Fig. 3f–h). In the past three decades, the share of global exposure in low sHDI group as a whole declined, while increasing in medium, high, and very high sHDI groups both for population and economic assets (Supplementary Fig. 5). These patterns in exposure coincide with observed global development trends^26^, particularly in India and China (Supplementary Fig. 6). Since 2000, climate-related disaster events have been increasingly reported in medium, high, and very high sHDI regions within these countries. This shift partly explains the increase in population exposure and reported impacts in the medium and high sHDI groups (Fig. 3f, g). The improvement in subnational human development is notable across Asia, which remains the main hotspot for population exposure and human loss (Supplementary Figs. 7 and 8). The same shift did not happen in Africa, where most reported events occurred in low sHDI regions throughout the study period (Supplementary Fig. 9). This heterogeneity reflects distinct country-level development trajectories within each continent (Supplementary Fig. 10). However, the majority of estimated economic exposure and reported losses continue to be concentrated in high and very high sHDI regions (Fig. 3d, e, i, j and Supplementary Fig. 11).

### Uneven impact trends across human development groups

**Fig. 4:**
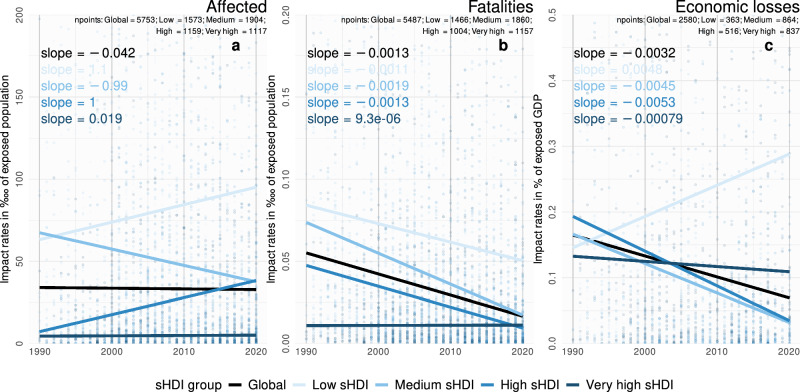
Trends in event-level disaster impact rates from 1990 to 2020 by sHDI group. Impact rates are defined as the ratio of reported impacts to potential exposure: affected or fatality counts divided by exposed population, and economic losses divided by exposed GDP. **a** Shows affected rates, **b** fatality rates (both in parts per 10,000 of exposed population), and **c** economic loss rates (as a percentage of exposed GDP), pooled across disaster types. Trends are estimated using quantile regression (*τ* = 0.5, the 0.5 quantile) for the global aggregate (black line) and for each sHDI group separately; slopes are indicated in the figure, and full results are reported in Supplementary Table 8. The number of observations per sHDI group and globally (*n*) is indicated in each panel.

To understand the dynamics of vulnerability and adaptive capacity, we used quantile regression to estimate robust median trends in impact rates (affected populations, fatalities, and economic losses), offering insights that are less sensitive to outliers and better reflect typical event outcomes. The analysis shows statistically significant trends (*p* < 0.05) estimated globally across different sHDI groups (Fig. 4). Overall, median fatality rates show a pronounced negative trend (Fig. 4b), declining 69.8% globally from 1990 to 2020 (Supplementary Table 8). Fatality rates in medium and high sHDI groups also show significant (*p* < 0.05) negative trends, with values converging to levels similar to those of very high sHDI regions by 2020. The higher fatality rates in low sHDI regions confirm the link between lower socioeconomic development and increased disaster fatalities. In 2020, the median fatality rate in low sHDI regions was approximately 0.05 per 10,000 people—three to five times higher than in other sHDI groups. This negative association between human development and disaster impacts holds consistently across continents and across all impact variables (Supplementary Fig. 12).

The overall reduction in fatality rates at both the global and sHDI group levels is largely driven by a decline in fatalities caused by floods and storms (Supplementary Fig. 13 and Supplementary Tables 9–13). For both disaster types, we observe a convergence among medium, high, and very high sHDI groups. In medium sHDI regions, median fatalities per event decreased by 78.6% for floods and 65.3% for storms (Supplementary Table 14). Although fatalities due to floods also declined significantly in low sHDI regions (by 61.2%), storm-related fatalities in these regions remain disproportionately—four to eight times—higher than in other sHDI groups. This global decline in vulnerability to floods is well documented in the literature^14,16,17^. While a recent study^19^ suggests limited progress in reducing vulnerability to floods after the year 2000, our findings strongly indicate substantial improvements during the period from 1990 to 2020. This discrepancy may be attributable to the larger sample size in our analysis (913 events from ref. ^19^, compared to 3694 in our sample for the whole study period and 3,099 events since 2000). Nonetheless, fatality rates in low sHDI regions remain disproportionately high.

Regarding other disaster types—storms, landslides, and droughts—residents of low and medium-HDI regions experience higher impact rates, in line with previous research^14^. Higher impact rates are observed for low sHDI regions for both human and economic losses (Supplementary Fig. 13). Only extreme temperature events (heat waves and cold waves) show a contrasting pattern. However, these disaster types represent only 5.5% of the events in our sample, so the lower rates may reflect a weak link between the hazard and the impacted regions’ vulnerability, or may result from limited data availability.

Despite substantial declines in the rates of fatalities and economic losses, the rate at which exposed people are affected in high sHDI regions increased significantly, particularly for floods, storms, landslides, and cold waves (Fig. 4a, Supplementary Fig. 13, and Supplementary Tables 14 and 16). This suggests that rapid urban expansion (especially in high HDI countries^31^) may increase exposure to hazards like floods^32,33^, storms, and landslides^34^ potentially limiting efforts to reduce vulnerability. While improved infrastructure and other social factors can limit fatalities and economic losses, they do not necessarily prevent exposed populations from being affected by these hazards in other ways.

Impact rates are imperfect proxies for vulnerability because they encode information about all elements of the disaster risk, including hazard intensity^10,11^. Nevertheless, their trends suggest underlying reductions in vulnerability across sHDI groups and continents. Low sHDI regions—concentrated mainly in Sub-Saharan Africa– experience the highest relative human and economic losses, consistent with higher underlying vulnerability. By contrast, Central and South America and large parts of Asia show more heterogeneous patterns, with many regions transitioning to medium and high sHDI levels. Overall, these disparities highlight the uneven global distribution of climate-related disaster impacts and the underlying disaster risk, as well as the importance of local development dynamics in shaping vulnerability. Consistent with these global patterns, the continent-level co-evolution trajectories of sHDI and disaster impacts also indicate gradual improvements in development alongside declining impact rates (Supplementary Figs. 14–16). However, these results do not isolate the influence of hazard intensity on the observed impact patterns. Typically, disaster impact and risk assessment studies^14,16–19^ omit the analysis of hazard intensity, and estimate vulnerability from the ratio of reported impacts to modeled or estimated exposure instead. In the following, we aim to address this limitation.

### Effects of hazard intensity on impacts are strongly modulated by development level

**Fig. 5:**
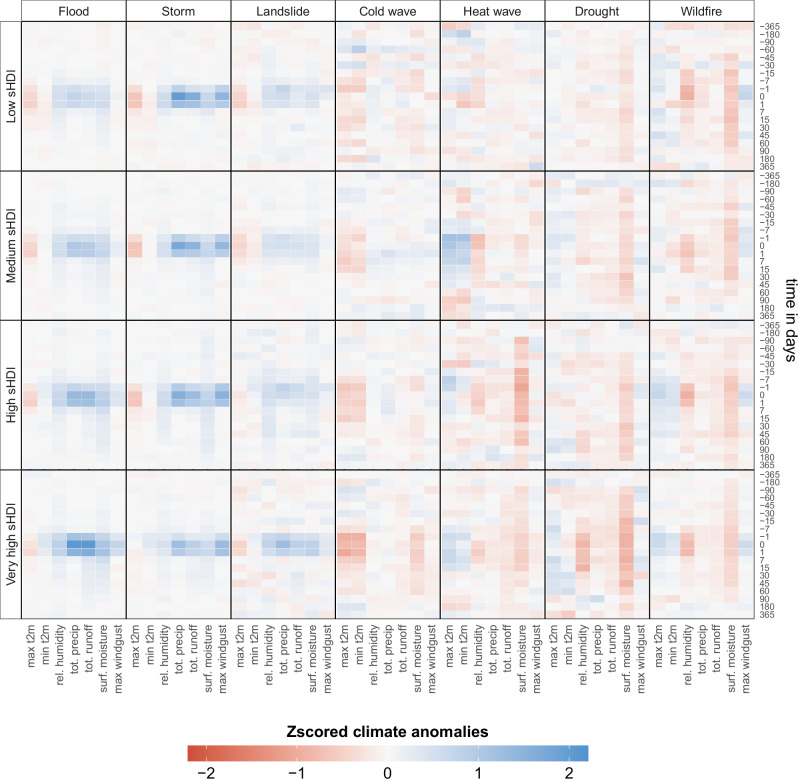
Composite climatological anomalies stratified by disaster type and sHDI. Average climate anomalies are shown per disaster type (columns) and sHDI group (rows). Each panel represents the temporal behavior of selected climate anomalies of a specific disaster type at selected dates ranging from one year before to one year after the reported start of the events (*y*-axis). Composite anomaly profiles were estimated using SEA^67^. All climate variables (*x*-axis) are standardized (zero mean, unit variance) for comparability. Color indicates the z-scored anomaly magnitude, with red denoting positive anomalies and blue denoting negative anomalies. Climate variables: maximum temperature at 2 m (max t2m), minimum temperature at 2 m (min t2m), relative humidity (rel. humidity), total precipitation (tot. precip), total runoff (tot. runoff), surface moisture (surf. moisture), and maximum wind gust (max windgust).

To understand the influence of hazard intensity on observed impacts, we calculated a composite of meteorological anomalies (Fig. 5) that describes the typical evolution of the hazard associated with each disaster type. Overall, the anomalies exhibit similar patterns across all sHDI regions: floods, storms, and landslides are associated with anomalously high precipitation, runoff, soil moisture, and relative humidity (Fig. 5 and Supplementary Fig. 17). In contrast, heat waves, droughts, and wildfires are associated with concurrent positive temperature anomalies and negative moisture anomalies. The stratification of the climate anomalies by sHDI group shows that hazards tend to be, on average, more intense in regions with higher levels of human development to be reported in EM-DAT (Fig. 5). This pattern, previously observed at the national scale^21^, suggests that higher human development is associated with a higher hazard intensity threshold required to result in a disaster (Supplementary Fig. 18). In contrast, in regions with low sHDI, higher vulnerability and variable adaptive capacity mean that weaker hazards can already lead to significant adverse impacts.

We also examine the association between impact rates and the intensity of hazard-related hydrometeorological anomalies. Overall, the distance correlation between impact rates and hazard intensity is weak, particularly for floods (Supplementary Fig. 19). However, when we stratify these correlations by sHDI groups, stronger and statistically significant associations emerge (Supplementary Fig. 20; *p* < 0.05). In the case of floods, the distance correlations between total daily precipitation, total daily runoff, and all impact rates are statistically significant and systematically higher in the low, medium, and high sHDI groups (Supplementary Fig. 20). For heat waves, higher rates of affected people and fatalities in very high sHDI regions are significantly correlated with maximum daily temperature. Not all correlations are meaningful, though. Low correlations in fatality rates due to floods do not imply that hazard intensity is unrelated to impacts, as disasters cannot occur without the hazard itself. Rather, we interpret these cases as instances where the effect of hazard intensity on impacts is strongly modulated by the socioeconomic status of the impacted region (i.e., sHDI) and the corresponding vulnerability levels. To assess whether these patterns persist when hazard intensity is held approximately constant, we further stratified flood and storm events into intensity bins. Across all comparable hazard intensity ranges, the development-impact gradients remain evident (Supplementary Figs. 21 and 22).

Together, these findings indicate that climate-related hazards need to be more intense to cause impacts in regions with higher levels of socioeconomic and human development. This underscores the importance of incorporating hazard intensity into impact and vulnerability assessments. Furthermore, it provides evidence of the higher vulnerability in low sHDI regions: less severe climate hazards result in higher impact rates, thereby putting these regions disproportionately at risk compared to other groups.

### Unequal human development, unequal disaster risk

To quantify the comparative risk of different disaster types per sHDI group, we calculated the odds of impacts within each sHDI group and compared them to those in very high sHDI regions, which serve as the baseline. A coefficient greater than 1 indicates a higher risk of impact in the considered sHDI group, whereas a coefficient less than 1 indicates a higher risk of impact in the very high sHDI group.

**Fig. 6:**
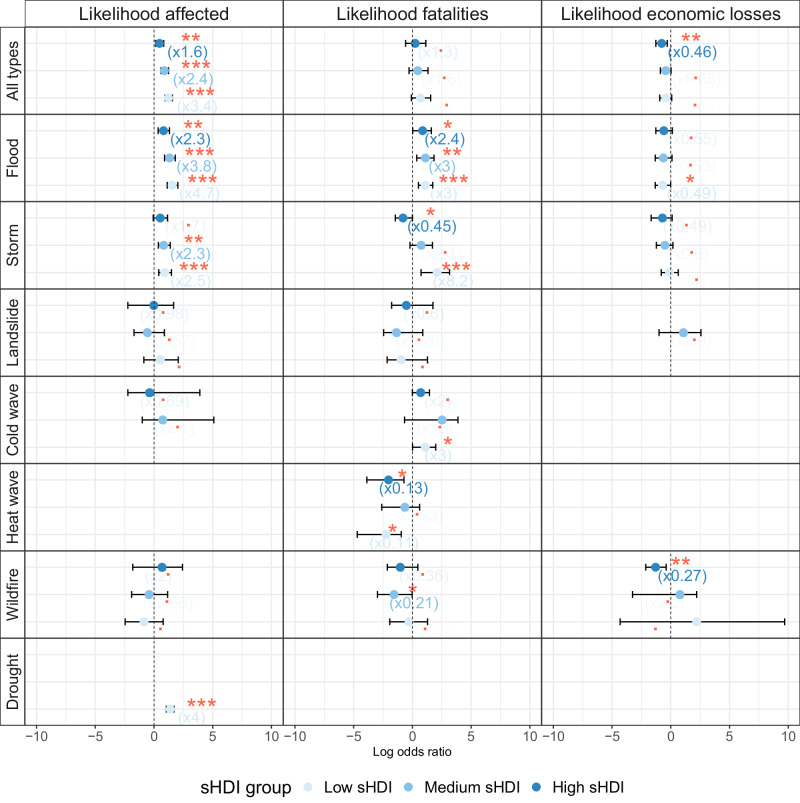
Odds ratios comparing disaster impact likelihood across subnational human development groups and disaster types. Odds ratios (ORs) from logistic regression comparing the likelihood of impacts - affected people, fatalities, and economic losses - between low, medium, and high sHDI groups and the very high sHDI reference group, shown both pooled across all disaster types and stratified by individual disaster type. Points represent ${\log }_{10}$-transformed odds ratios (${\log }_{10}({{\rm{OR}}})$; *x*-axis), while adjacent labels indicate the corresponding OR values. The vertical dashed line (${\log }_{10}({{\rm{OR}}})=0$) denotes no difference relative to the reference group (OR = 1); OR > 1 indicates a higher likelihood of impact occurrence, whereas OR < 1 indicates a lower likelihood relative to the very high sHDI group. The horizontal bars indicate 95% confidence intervals estimated by bootstrapping the logistic regression coefficients (5000 iterations). Asterisks indicate statistical significance (non-significant, ^*^p < 0.05, ^**^p < 0.01, ^***^p < 0.001).

Given the available data, significant odds ratios (*p* < 0.05) show that the likelihood of human losses—both people affected and fatalities—is disproportionately higher in low and medium sHDI groups (Fig. 6; *p*-values and 95% CI in Supplementary Tables 17–19). For example, when exposed, a resident of a low sHDI region faces, on average, a risk of fatalities three times higher due to floods and 8.2 times higher due to storms, compared to a resident of a very high sHDI region. In high sHDI regions, these comparative risk factors drop to 2.4 for floods and 0.45 for storms. The likelihood of being affected shows a similar pattern; overall, across all disaster types, the risk is 3.4 times higher in low sHDI, 2.4 times in medium sHDI, and 1.6 times in high sHDI than in regions with very high sHDI.

Heat waves are an exception. The risk of human loss from heat waves is significantly higher in very high sHDI regions (Fig. 6). On average, fatalities in these regions are nine times higher than in low sHDI regions and 7.9 times higher than in high sHDI regions. One potential explanation is that the estimated vulnerability or resilience to one specific hazard type is not necessarily applicable to another, since different hazard types have distinct impact mechanisms and, consequently, different vulnerability factors. In this case, demographics may play an important role, as aging populations—more prevalent in very high sHDI regions—are more vulnerable to heat mortality^35^. However, the reported odds ratios only reflect the events included in our dataset. Therefore, another explanation could be reporting bias for heat waves: such events may be underreported in low and medium sHDI groups^36,37^. Very high sHDI regions, where impact estimates are also of higher quality^37^, account for 58% of reported heat waves.

Similarly, in the case of droughts, we hypothesize that the calculated coefficient may not reflect the real disparity in comparative risk for low sHDI regions—the sHDI group where the affected numbers and rates associated with droughts have the sharpest increase (Supplementary Figs. 5 and 13). In very high sHDI regions, too few droughts are considered and reported as climate-related disaster events, which limits comparability. In high and very high sHDI regions, droughts are perceived mainly as financial or insurance issues, unlike in low sHDI regions, where droughts represent food security threats^38^.

Regarding economic losses, we find that very high sHDI regions are at greater risk in only a few cases. For example, they face greater risk compared to high sHDI regions across all disaster types, and compared to low sHDI regions specifically in the case of floods. The absence of significant coefficients in most other cases —especially for floods and storms, where far more observations are available— suggests comparable risk levels of relative economic losses. This finding is supported by the comparable levels of economic loss rates observed across storms and floods (Fig. 4c and Supplementary Fig. 13), where sample sizes are robust (Table 1). For the low sHDI group, and landslides and wildfires as a whole, data are scarce, and results should be interpreted with caution. Overall, the absolute economic losses are orders of magnitude higher in very high sHDI regions compared to all other groups (Fig. 3e and Supplementary Fig. 4). However, event-level consequences may be more severe for low and medium sHDI regions due to reduced insurance penetration^39^ and weaker social safety nets in general.

**Table 1:** Counts and percentages of missing values for the three impact variables (number of affected people, number of fatalities, and economic losses) per disaster type and sHDI group

Variable	Number affected (*n* missing)	Number affected (% missing)	Number fatalities (*n* missing)	Number fatalities (% missing)	Economic losses (*n* missing)	Economic losses (% missing)	*n*
Total	1308	18.50	1574	22.30	4481	63.50	7061
Hazard: Flood	387	10.50	911	24.70	2508	67.90	3694
Hazard: Storm	427	20.40	379	18.10	964	46.10	2090
Hazard: Landslide	186	35.70	19	3.60	456	87.50	521
Hazard: Cold wave	155	59.60	39	15.00	240	92.30	260
Hazard: Heat wave	85	68.00	8	6.40	109	87.20	125
Hazard: Wildfire	43	18.00	95	39.70	124	51.90	239
Hazard: Drought	25	18.90	123	93.20	80	60.60	132
sHDI: Low sHDI	267	14.50	374	20.30	1477	80.30	1840
sHDI: Medium sHDI	345	15.30	389	17.30	1385	61.60	2249
sHDI: High sHDI	210	15.30	365	26.70	853	62.30	1369
sHDI: Very high sHDI	486	30.30	446	27.80	766	47.80	1603
Flood/Low sHDI	124	10.80	252	22.00	926	80.90	1145
Flood/Medium sHDI	118	9.60	236	19.20	794	64.70	1228
Flood/High sHDI	65	8.70	213	28.60	490	65.90	744
Flood/Very high sHDI	80	13.90	210	36.40	298	51.60	577
Storm/Low sHDI	57	14.10	56	13.90	289	71.50	404
Storm/Medium sHDI	73	11.50	86	13.60	267	42.10	634
Storm/High sHDI	47	12.60	87	23.30	164	44.00	373
Storm/Very high sHDI	250	36.80	150	22.10	244	35.90	679
Landslide/Low sHDI	57	35.00	1	0.60	150	92.00	163
Landslide/Medium sHDI	98	39.20	10	4.00	222	88.80	250
Landslide/High sHDI	26	35.60	4	5.50	61	83.60	73
Landslide/Very high sHDI	5	14.30	4	11.40	23	65.70	35
Cold wave/Low sHDI	17	54.80	1	3.20	30	96.80	31
Cold wave/Medium sHDI	26	53.10	14	28.60	44	89.80	49
Cold wave/High sHDI	38	41.30	19	20.70	85	92.40	92
Cold wave/Very high sHDI	74	84.10	5	5.70	81	92.00	88
Heat wave/Low sHDI	10	62.50	0	0.00	15	93.80	16
Heat wave/Medium sHDI	12	66.70	2	11.10	16	88.90	18
Heat wave/High sHDI	9	50.00	4	22.20	15	83.30	18
Heat wave/Very high sHDI	54	74.00	2	2.70	63	86.30	73
Wildfire/Low sHDI	1	6.70	4	26.70	12	80.00	15
Wildfire/Medium sHDI	13	35.10	11	29.70	25	67.60	37
Wildfire/High sHDI	15	32.60	15	32.60	31	67.40	46
Wildfire/Very high sHDI	14	9.90	65	46.10	56	39.70	141
Drought/Low sHDI	1	1.50	60	90.90	55	83.30	66
Drought/Medium sHDI	5	15.20	30	90.90	17	51.50	33
Drought/High sHDI	10	43.50	23	100.00	7	30.40	23
Drought/Very high sHDI	9	90.00	10	100.00	1	10.00	10

The four sHDI groups follow the UNDP thresholds: low (sHDI < 0.55), medium (0.55 ≤ sHDI  < 0.70), high (0.70 ≤ sHDI < 0.80), and very high (sHDI ≥ 0.80).

**Fig. 7:**
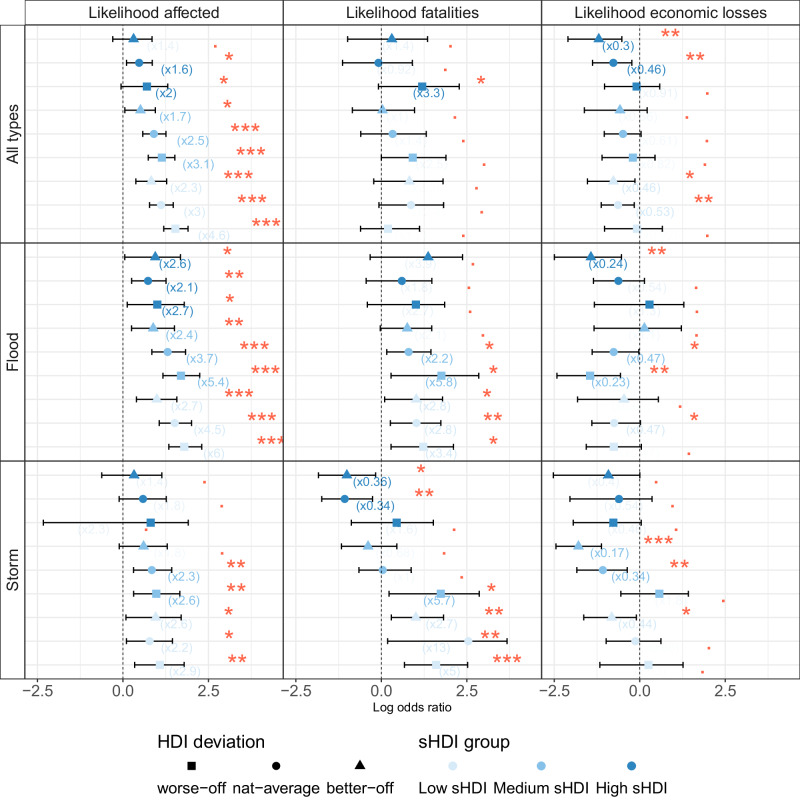
Odds ratios comparing disaster impact likelihood across subnational human development groups, stratified by deviation from national HDI and disaster types. Odds ratios (ORs) from logistic regression comparing the likelihood of impacts - affected people, fatalities, and economic losses—between the very high sHDI reference group and all combinations of sHDI group (low, medium, and high) and deviation from national HDI (better-off, national-average, worse-off), shown pooled across all disaster types and separately for floods and storms. Points represent ${\log }_{10}$-transformed odds ratios (${\log }_{10}({{\rm{OR}}})$; *x*-axis), while adjacent labels indicate the corresponding OR values. The vertical dashed line (${\log }_{10}({{\rm{OR}}})=0$) denotes no difference relative to the reference group (OR = 1); OR > 1 indicates a higher likelihood of impact occurrence, whereas OR < 1 indicates a lower likelihood relative to the very high sHDI group. The shape represents the deviation category (better-off, national-average, or worse-off relative to national HDI), and color indicates the sHDI group. Horizontal bars indicate 95% confidence intervals estimated by bootstrapping the logistic regression coefficients (5000 iterations). Asterisks indicate statistical significance (non-significant, ^*^p < 0.05, ^**^p < 0.01, ^***^p < 0.001).

To account for intra-country inequalities in the risk assessment, we recalculated the comparative risk coefficients using very high sHDI as the baseline, comparing all other sHDI groups stratified by the deviation from national HDI. Due to the limited number of observations available for most disaster types, we only present results for floods, storms, and all disasters combined. Although the recalculated comparative risk coefficients follow similar patterns to the previous analysis, the risk of human loss is amplified for lower human development levels associated with negative deviations from the national HDI (Fig. 7; *p*-values and CI in Supplementary Tables 20–22).

In medium and low sHDI regions, deviations from the national HDI have a clear influence on the likelihood of impact. The risk of being affected, across all disaster types, is higher in subgroups with negative deviations from national HDI (worse-off) and lower in subgroups with positive deviations (better-off) or those matching the national average. This effect is particularly pronounced for floods and storms. Overall, these results indicate that both between-group and within-group sHDI inequalities are relevant for determining risk. More importantly, the most vulnerable people are typically the most disadvantaged in terms of human development at both levels.

However, there are some diverging patterns. For example, in low sHDI regions, the highest risk of fatalities from storms is observed in the national-average subgroup (13 times higher than in very high sHDI) rather than in the worse-off subgroup (5 times higher). Such discrepancies may be attributed to natural factors, like geographic location or topography, which can exacerbate vulnerabilities beyond regional development levels. Another notable exception is the worse-off subgroup of the medium sHDI regions. In all examined cases, the comparative risk of human loss is distinctly higher for this subgroup and approaches levels observed in low sHDI subgroups. In fact, medium sHDI regions show the widest range of risk coefficients, with the better-off subgroup exhibiting similar risk levels to high sHDI regions. Given that medium sHDI regions have the widest range of deviations from the national HDI (Fig. 1b), this emphasizes the importance of considering both between-group and within-group inequalities in human development when estimating the risk of adverse impacts caused by climate-related hazards.

## Discussion

This study presents a global multi-hazard assessment of disaster impacts and risk that exclusively relies on sHDI and integrates climatological event characterization. Our analyses demonstrate that human development is closely linked to disaster impacts, as it shapes the critical context in which hazards and exposure interact to produce impacts. Impact rates clearly decline with increasing levels of human development, from medium to high and very high sHDI groups. This trend persists despite the shift in exposure between 1990 and 2020, which leads to higher sHDI groups (medium, high, and very high) having increasing shares of exposed populations. However, the low sHDI regions continue to experience disproportionately high impact rates, even as their share of global exposure declines. Compared to the very high sHDI group, these regions exhibit higher risk levels for human losses and comparable risk levels for economic losses, also in the case of lower hazard intensities. Furthermore, higher within-country inequality levels in low and medium sHDI regions amplify the risk of human losses, especially in the cases of floods and storms. Having established these global patterns, we next examine the underlying mechanisms that link human development to disaster impacts.

Disaggregating sHDI into its components (Fig. 2) shows that low sHDI values are mainly driven by low education and income levels, while health contributes relatively more. This pattern suggests that higher vulnerabilities in low sHDI regions are largely associated with deficits in education and income. While our approach does not causally test these roles, we know from a disaster risk perspective that education is often linked to preventive capacity-supporting preparedness, awareness, and institutional capability^40,41^; health systems, instead, are primarily associated with post-disaster response and recovery^39,42^; and income plays a dual role by enabling preventive investments in infrastructure and providing resources for recovery^15,39^. Taken together, these component differences inform the vulnerability profiles of impacted regions.

The disparities in human development and its underlying dimensions reflect differences in vulnerability and adaptive capacity among sHDI groups. Previous studies have suggested a general decline in vulnerability to climate-related hazards globally^14,16,17^. However, our analysis at the subnational level shows a decline in impact rates for low sHDI regions only in the case of floods. Despite progress in reducing flood vulnerability^17^, the risk of human loss in low sHDI regions remains significantly higher than in other groups. Moreover, our findings suggest that future risk may increase in high sHDI regions, especially for floods, storms, and landslides, due to rising exposure.

**Fig. 8:**
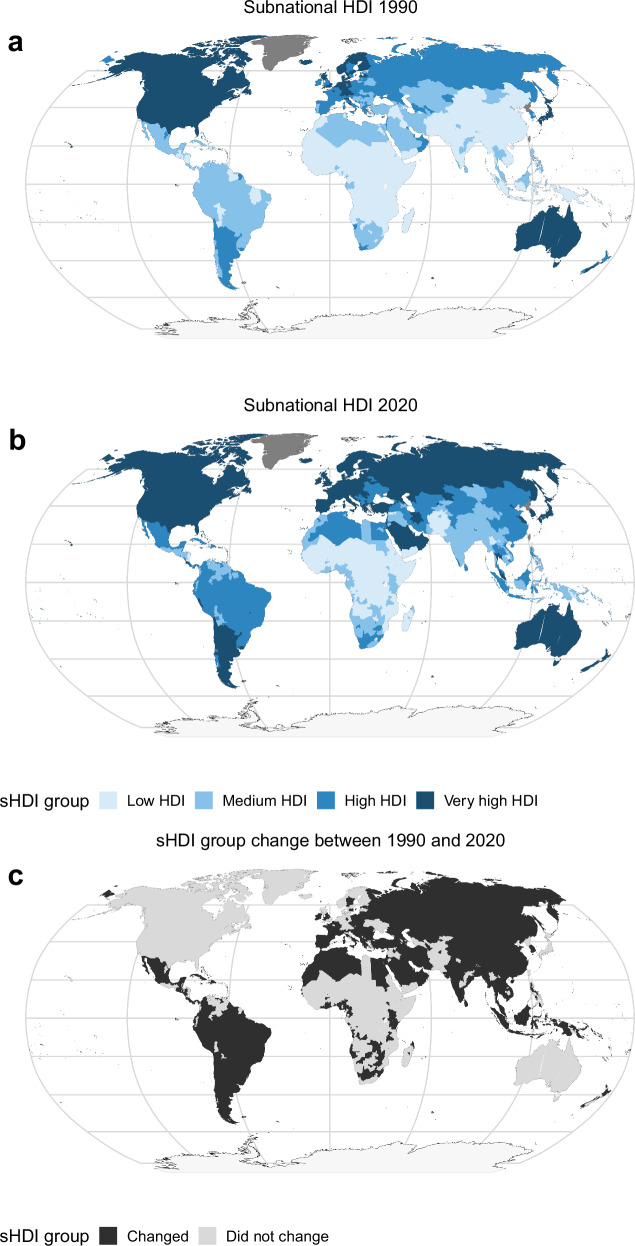
sHDI trajectories between 1990 and 2020. The maps show **a** the sHDI groups in 1990; **b** sHDI groups in 2020; **c** regions that changed sHDI group between 1990 and 2020 (dark) versus those that did not (light). Gray areas indicate regions with missing sHDI data in 1990, 2020, or both. The four sHDI groups follow the UNDP human development thresholds: low (<0.55), medium (0.55 ≤ sHDI < 0.70), high (0.70 ≤ sHDI < 0.80), and very high (sHDI ≥ 0.80). sHDI data and administrative boundaries are from the Global Data Lab (globaldatalab.org). Map projection: Robinson.

Despite the observed decreases in the relative share of population and economic exposure to climate-related hazards, low sHDI regions will remain at high risk in the foreseeable future. By 2020, sub-Saharan Africa encompassed the majority of low-sHDI regions (Fig. 8). Projections of future demographic growth^43^ suggest that the largest population increase will occur in Sub-Saharan Africa^44^, with rapid urban expansion placing a growing number of people in hazard-exposed areas^45,46^. At the same time, climate change is projected to slow income growth and hinder economic convergence in the region^47^. When combined with high levels of vulnerability, these demographic and socioeconomic trends imply that impacts and risk are likely to increase considerably in low sHDI regions in the coming decades^23^. However, tracking this evolution is challenging, as reliable data are often scarce in these areas.

Our results must be interpreted in light of key limitations in the human development metrics and the disaster impact data. The sHDI inherits the well-known constraints of the national HDI, which condenses complex socio-economic realities into a single number, ignores non-linear data dependencies, and therefore leaves much variance unexplained^48^. While sHDI provides important structural information, it is not a disaster-specific vulnerability index and should not be interpreted as such. For instance, it does not capture governance quality or local environmental and infrastructural conditions that influence disaster impacts. A second set of limitations concerns the availability and completeness of disaster impact data, which remain uneven, particularly in low sHDI regions. For example, impact reporting for heat waves is especially sparse despite well-studied biophysical exposure^49–52^. Moreover, the higher share of missing economic loss estimates for disasters reported in these regions (Table 1) reduces the robustness of comparative analyses. Impact underreporting and incomplete loss accounting affect most disaster datasets^53^: EM-DAT does not capture every climate-related disaster^54,55^, and its impact estimates involve uncertainty, although human loss figures are generally more reliable and less prone to systematic bias^56^. Event reporting may also be shaped by political realities that are difficult to correct objectively^36^. Although EM-DAT has improved its methodology and documentation^55^, these updates do not resolve the underlying reporting gaps and regional inconsistencies. The geocoding approach adopted here provides improved spatial alignment with EM-DAT compared to earlier studies^57^, but it cannot compensate for incomplete or missing impact information. Complementary impact datasets, including national disaster inventories and emerging satellite-based assessments, offer promising avenues for validation and improved coverage in regions with sparse reporting. Integrating and harmonizing such sources in future work could substantially enhance empirical assessments of disaster impacts.

Despite these limitations, several actionable insights emerge regarding pathways to reduce disaster risk. Vulnerability and adaptive capacity are critical levers for reducing disaster risk. Climate change and land surface transformations that exacerbate climate hazards^58^ are likely to persist over the coming decades due to past and ongoing greenhouse gas emissions and land use changes^59^. Abrupt and substantial changes in exposure are also unlikely, as current exposure patterns are projected to increase^44^. Planning large-scale community relocation to less exposed areas is impractical and ethically questionable^60^. Reducing vulnerability and building adaptive capacity, particularly in low sHDI regions, is the most time and resource-efficient approach to reducing impacts and risks. However, in this process, the observed hazard-specific nature of vulnerability must be recognized, as resilience to one hazard type does not guarantee resilience to another. While vulnerability to floods has been extensively studied and documented^16–19^, other hazard types, such as storms and landslides, have received comparatively less attention. Hot and dry events (heat waves, wildfires, and droughts) are also understudied and present biases even in data collection and reporting. Therefore, there is a need for disaster-specific and empirical impact-based vulnerability assessments to match the progress achieved in predicting the evolution of climate hazards and future exposure.

Both hazard intensity^61^ and exposure^62^ are expected to increase in the near future as a consequence of climate change, which reinforces the importance of vulnerability reduction. People in low sHDI regions will face tremendous challenges in the coming decades. This raises important questions about the development pathways available to these regions, and challenges our current perception of climate justice^63^ and adaptation^64^. Yet, the dependence between development and disaster impacts is not unidirectional. When hazards strike frequently or with great intensity, they can lock vulnerable communities into persistent low-development traps^13^. Such a vicious cycle erodes human development gains and perpetuates increased vulnerability^39^. This underscores the importance of post-disaster recovery efforts that not only restore, but also reinforce resilience and human capital. Therefore, addressing both global and intra-country inequalities is crucial for effective and just climate action that benefits everyone.

## Methods

### Geocoding of disaster events

We investigate EM-DAT disaster events reported between 1990 and 2020 that were geocoded by the Geo-Disasters database^57^. This geocoding is based on the Global Administrative Unit Layers (GAUL) database at administrative levels 1 and 2^65^, the reference administrative database used by EM-DAT. All other information about the events is extracted from EM-DAT (disaster type, start and end dates, human and economic losses). To limit spatial uncertainty, we excluded events with a total area larger than one million square km. EM-DAT reports impacts at the subnational administrative unit level rather than the hazard footprint^57^, and subnational administrative regions are not necessarily fully impacted by a hazard^66^, meaning that aggregation uncertainty grows with area. The vast majority of events in our sample fall well below this threshold, with a median impacted area of 27,004 km^2^ and a 75th percentile of 125,003 km^2^.

For event classification, we primarily followed the EM-DAT^28^ disaster typology. We directly used the EM-DAT disaster types for flood, storm, wildfire, and drought events. We divided extreme temperature events into two groups based on the indicated disaster subtype: heat wave for heat-related events (subtype: heat wave) and cold wave for cold-related events (subtypes: cold wave and severe winter conditions). Finally, we classified all events under the mass movement type as landslides. The disaster type classification is summarized in Supplementary Table 23.

### Composite climate anomalies

We estimated the composite climate anomalies of each disaster type (Supplementary Fig. 17) and of the different disaster types by sHDI group (Fig. 5) using the Superposed Epoch Analysis (SEA), or composite analysis^67^. Climate variables were obtained from the European Centre for Medium-Range Weather Forecasts Reanalysis v5 (ERA5)^68^ and the Global Land Evaporation Amsterdam Model (GLEAM)^69^ datasets. Relative humidity was derived from ERA5 variables.

For each event, we extracted six years of daily climate data (variables listed in Table 2) and spatially aggregated it by calculating the daily average, producing a single time series for each variable per event. The time series of standardized anomalies was then aligned with the reported event start dates, and the average was computed across all events to estimate the typical event profile. Using a fixed multi-year window is standard practice in SEA applications in climate impact research, as it provides a consistent temporal frame for averaging heterogeneous events and allows comparison across disaster types. The time series of standardized climate anomalies (Eq. 1) was calculated for each variable *x*.

1 $${{{\rm{Anomaly}}}}_{x}(t)=\frac{x(t)-{\mu }_{x,{{\rm{month}}}}}{{\sigma }_{x,{{\rm{clim}}}}}$$

where *μ*_*x*,month_ is the long-term monthly mean and ${\sigma }_{x,{{\rm{clim}}}}$ is the climatological standard deviation.

**Table 2:** List and sources of the climate variables used in the analysis

Variable	Long name	Units	Source
t2mmax	Maximum 2m temperature	°C	ERA5
t2mmin	Minimum 2m temperature	°C	ERA5
rh	Relative humidity	%	Derived from ERA5
smsurf	Surface soil moisture	mm day^−1^	GLEAM
tp	Total precipitation	mm day^−1^	ERA5
tro	Total runoff	mm day^−1^	ERA5
windgustmax	Maximum wind gust	km h^−1^	ERA5

*ERA5* fifth-generation European Centre for Medium-Range Weather Forecasts (ECMWF) reanalysis, *GLEAM* global land evaporation Amsterdam model.

The SEA is used for descriptive context only (Fig. 5 and Supplementary Fig. 17); event-level anomalies used in the correlation and stratified analyses are computed separately as described in the Distance correlation subsection.

### Distance correlation

We used distance correlation^70^ to assess the strength of association between impact rates (i.e., ratios of reported impacts to the number of exposed people or exposed GDP for each event) and climate anomalies characterizing the hazards associated with each disaster event. Unlike Pearson correlation, which is limited to linear relationships, distance correlation captures a broader range of dependencies, including complex and non-monotonic patterns that are often present in climate-society interactions. The distance correlation coefficient ranges from 0 to 1, where 0 indicates no dependence, and 1 indicates perfect dependence. Hazard anomalies used in the analysis are computed with a ±3-day window around the reported event start date to capture its peak intensity. The event-specific climate anomalies are defined using maximum daily temperature, total daily precipitation, total daily runoff, relative humidity, and maximum daily wind gust, and the minimum values for minimum daily temperature and daily surface moisture.

For each event in our sample, we define impact rates as the ratio between the reported impacts and potential exposure for both human (Eq. 2), and economic (Eq. 3) losses.

2 $${{\rm{Impact}}}\,{{{\rm{Rate}}}}_{{{\rm{people}}}}=\frac{{{\rm{Affected}}}\,{{\rm{or}}}\,{{\rm{Fatalities}}}}{{{\rm{Exposed}}}\,{{\rm{Population}}}}$$

3 $${{\rm{Impact}}}\,{{{\rm{Rate}}}}_{{{\rm{economic}}}}=\frac{{{\rm{Economic}}}\,{{\rm{Losses}}}}{{{\rm{Exposed}}}\,{{\rm{GDP}}}}$$

Additionally, we performed a hazard intensity-stratified comparison of impact rates for floods and storms to assess whether development-impact differences could be confounded by variations in hazard intensity. The results are presented in Supplementary Figs. 21 and 22.

### Impact data

Human and economic loss data are provided by the EM-DAT database^28^. We selected variables for total affected people, total fatalities, and total economic losses. The three impact variables are not available for all of the events. The number of available observations and the fraction of missing values are reported in Table 1, including a full breakdown by disaster type and sHDI group. Only reported values are analyzed, as validated methods for unbiased imputation of disaster impact data are not currently available.

We adjusted the total economic losses to constant 2011 USD (Eq. 4) to allow for joint use with the GDP data and to estimate economic loss as a fraction of GDP. We used the Consumer Price Index data (CPI) provided by EM-DAT, using 2011 as the base year for the conversion.

4 $${{{\rm{Loss}}}}_{2011\,{{\rm{USD}}}}=\frac{{{{\rm{CPI}}}}_{2011}}{{{{\rm{CPI}}}}_{{{\rm{year}}}}}\,{{{\rm{Loss}}}}_{{{\rm{year}}}{{\rm{USD}}}}$$

### Population and economic exposure

The Global Human Settlement (GHS) population grid^71^ was used to estimate population exposure, and to normalize the human losses impact variables. Since population counts are only available for the years 1990, 1995, 2000, 2005, 2015, and 2020, we filled the gaps for the intervening years using linear interpolation. Similarly, we used the gridded global dataset for GDP^72^. We applied linear interpolation between 1990 and 2015, and linear extrapolation from 2015 to 2020, to estimate yearly GDP values. We estimated potential population and economic exposure by summing the population counts and GDP values corresponding to each reported event area in the year of the event.

Previous studies estimated exposure in the case of floods using simulated proportions of populations exposed to a 100-year return period^19^ or used other modeling approaches^16,17^. Because such approaches are specific to floods and come with their own limitations^73,74^, we chose to rely entirely on EM-DAT reporting. To maintain a consistent approach across disaster types, we considered all inhabitants and the corresponding GDP within the reported region to be potentially exposed. For simplicity, we refer to potential exposure—both population and GDP-based economic exposure—in the text as exposure.

### Impact rates trend analysis

We estimated the trends in impact rates using quantile regression at the median (*τ* = 0.5). Statistical significance was assessed at the 5% level using 95% confidence intervals derived from the regression fits. Quantile regression is less sensitive to outliers than ordinary least squares regression, and, therefore, provides a more robust estimate of trends in impact rates. Given the highly skewed distributions of variables representing human and economic losses, quantile regression offers a more accurate representation of underlying trends by focusing on the median rather than the mean. This approach reduces the influence of extreme values, and provides clearer insights into the typical behavior of the data. Numerous studies have used quantile regression to estimate trends for similar variables^75^ or different variables^76,77^.

### sHDI

We use the 2023 release (7th update) of the Global Data Lab (GDL) sHDI^27^, which provides annual sHDI estimates and their components (education, health, income) for administrative level-1 regions from 1990 to 2020.

In cases where disaster events spanned multiple administrative regions, we calculated a population-weighted average to estimate the overall sHDI, or component (education, health, income) of the impacted area. In a small number of cases involving short missing sequences, we applied a conservative gap-filling procedure using linear interpolation for up to five consecutive years; gaps exceeding this limit were treated as missing, and the corresponding events were removed.

We classified regions into four development groups using thresholds consistent with the Human Development Reports^78^ from the UNDP; low (sHDI < 0.55; *n* = 1840), medium (0.55 ≤ sHDI < 0.7; *n* = 2249), high (0.7 ≤ sHDI < 0.8; *n* = 1369), and very high (sHDI ≥ 0.8; *n* = 1603).

We assess inequalities in human development across two levels: first, between the different sHDI groups (low, medium, high, and very high); second, within each of these groups using the deviation of the sHDI from the national HDI of the country in the event year (Eq. 5).

A region is considered better off than the national average if its sHDI deviation from the national HDI falls at or above the 80th percentile of the distribution of all deviations. Conversely, if the deviation falls at or below the 20th percentile, the region is classified as worse off or lagging behind the national average. Regions with sHDI deviations between the 20th and 80th percentiles are considered to have a development level comparable to the national average.

5 $${{{\rm{Deviation}}}}_{{\mbox{region}},{\mbox{year}}}={{{\rm{sHDI}}}}_{{\mbox{region}},{\mbox{year}}}-{{{\rm{HDI}}}}_{{\mbox{country}},{\mbox{year}}}$$

### Odds ratio calculation

Odds ratios express risk differences between groups. In our case, we used odds ratios to quantify the likelihood of impact—specifically, being affected, sustaining fatalities, or incurring economic losses—in low, medium, or high sHDI regions, compared to very high sHDI regions.

Using Eq. (6), we compared the likelihood of impact—being affected, sustaining fatalities, or incurring economic losses—across sHDI groups and disaster types, using the very high sHDI group as the baseline. This allowed us to quantify the relative risk in low, medium, and high sHDI regions, providing a metric for the differential impact of disasters across varying levels of human development.

6 $${{\rm{OR}}}=\frac{{{{\rm{Odds}}}}_{x}}{{{{\rm{Odds}}}}_{{{\rm{VH}}}}}$$

where OR represents the odds ratio. Odds_*x*_ denotes the odds of impacts in the group of interest, with *x* indicating the sHDI group. Odds_VH_ denotes the odds of impacts in the very high sHDI group.

In the second step (Eq. 7), we examined how the likelihood of the same impacts varied within each sHDI group when stratified by deviation from national HDI, again using the very high sHDI group as the baseline.

7 $${{\rm{OR}}}=\frac{{{{\rm{Odds}}}}_{x,y}}{{{{\rm{Odds}}}}_{{{\rm{VH}}}}}$$

where OR represents the odds ratio. Odds_*x*,*y*_ denotes the odds of impacts in the group of interest, with *x* indicating the sHDI group and *y* the deviation from national HDI. Odds_VH_ denotes the odds of impacts in very high sHDI regions.

For each case, logistic regression with 5000 bootstrap iterations was used to estimate 95% confidence intervals and *p*-values of the odds ratio coefficients. The number of observations per case is reported in Supplementary Tables 16–21. Cases with fewer than 30 observations were excluded from the analysis.

## Supplementary information

Supplementary information

Transparent Peer Review file

## Source data

Source Data

## Data Availability

The data used in this study are derived from publicly accessible sources. Information about the disaster events, including disaster types, reported impacts, start and end dates, and country of occurrence, are available from the EM-DAT (https://public.emdat.be/), which requires free registration to access as mandated by the data provider’s licensing terms. The geocoded locations of disaster events are available via Zenodo^57^ (https://zenodo.org/records/15487667). The sHDI and its components (education, health, and income indices) are provided by the Global Data Lab (https://globaldatalab.org/shdi/download/), which requires free registration to access as mandated by the data provider’s terms of use. Gridded data used to estimate population and economic exposure are available from the Global Human Settlement population grid (GHS-POP) (https://human-settlement.emergency.copernicus.eu/ghs_pop2023.php) and from the gridded GDP dataset (10.5061/dryad.dk1j0). Climate data used to characterize hazard intensities are obtained from the ERA5 reanalysis (https://www.ecmwf.int/en/forecasts/dataset/ecmwf-reanalysis-v5) and the GLEAM surface soil moisture dataset (https://www.gleam.eu/). Due to licensing restrictions, EM-DAT data cannot be redistributed. We provide all derived analysis-ready datasets and a script that automatically integrates EM-DAT data obtained directly from the original source, reconstructing all inputs required to reproduce the figures and analyses presented in the paper. Source data are provided with this paper.

## Code availability

The code and analysis-ready data used in the analysis^79^ are available at https://github.com/khalilT/inequality-hdev-disaster-riskand archived at 10.5281/zenodo.19361064.
