# Supplementary material for: Inequality in human development amplifies climate-related disaster risk
Source: Nat Commun. 2026 Jun 17;17:5067. doi: 10.1038/s41467-026-73873-9 (PMC13276189; doi:10.1038/s41467-026-73873-9)
Supplement: Supplementary file 1 — Supplementary information [file 41467_2026_73873_MOESM1_ESM.pdf]

Supplementary information for: Inequality in human development amplifies climate-related disaster risk

Khalil Teber[1*], Sebastian Sippel[2], Melanie Krause[3], Jakob Zscheischler[4, 5, 6], Miguel D. Mahecha[1, 4, 6]

[1*]Institute for Earth System Science and Remote Sensing, Leipzig University, Leipzig, 04103, Germany.
[2]Leipzig Institute for Meteorology, Leipzig University, Leipzig, 04103, Germany.
[3]Faculty for Economics and Management Sciences, Leipzig University, Leipzig, 04103, Germany.
[4]Helmholtz Centre for Environmental Research – UFZ, Leipzig, 04318, Germany.
[5]Department of Hydro Sciences, TUD Dresden University of Technology, Dresden, Germany.
[6]Center for Scalable Data Analytics and Artificial Intelligence (ScaDS.AI), Dresden/Leipzig, Germany.

*Corresponding author(s). E-mail(s): khalil.teber@uni-leipzig.de;

List of supplementary figures

Supplementary Figure 1 4
Supplementary Figure 2 5
Supplementary Figure 3 6
Supplementary Figure 4 7
Supplementary Figure 5 8
Supplementary Figure 6 9
Supplementary Figure 7 10
Supplementary Figure 8 11
Supplementary Figure 9 12
Supplementary Figure 10 13
Supplementary Figure 11 14
Supplementary Figure 12 15
Supplementary Figure 13 16
Supplementary Figure 14 17
Supplementary Figure 15 18
Supplementary Figure 16 19
Supplementary Figure 17 20
Supplementary Figure 18 21
Supplementary Figure 19 22
Supplementary Figure 20 23
Supplementary Figure 21 24
Supplementary Figure 22 25

List of supplementary tables

Supplementary Table 1 26
Supplementary Table 2 26
Supplementary Table 3 26
Supplementary Table 4 27
Supplementary Table 5 29
Supplementary Table 6 30
Supplementary Table 7 31
Supplementary Table 8 32

Supplementary Table 9 33
Supplementary Table 10 34
Supplementary Table 11 35
Supplementary Table 12 36
Supplementary Table 13 37
Supplementary Table 14 38
Supplementary Table 15 39
Supplementary Table 16 40
Supplementary Table 17 41
Supplementary Table 18 42
Supplementary Table 19 43
Supplementary Table 20 44
Supplementary Table 21 45
Supplementary Table 22 46
Supplementary Table 23 47

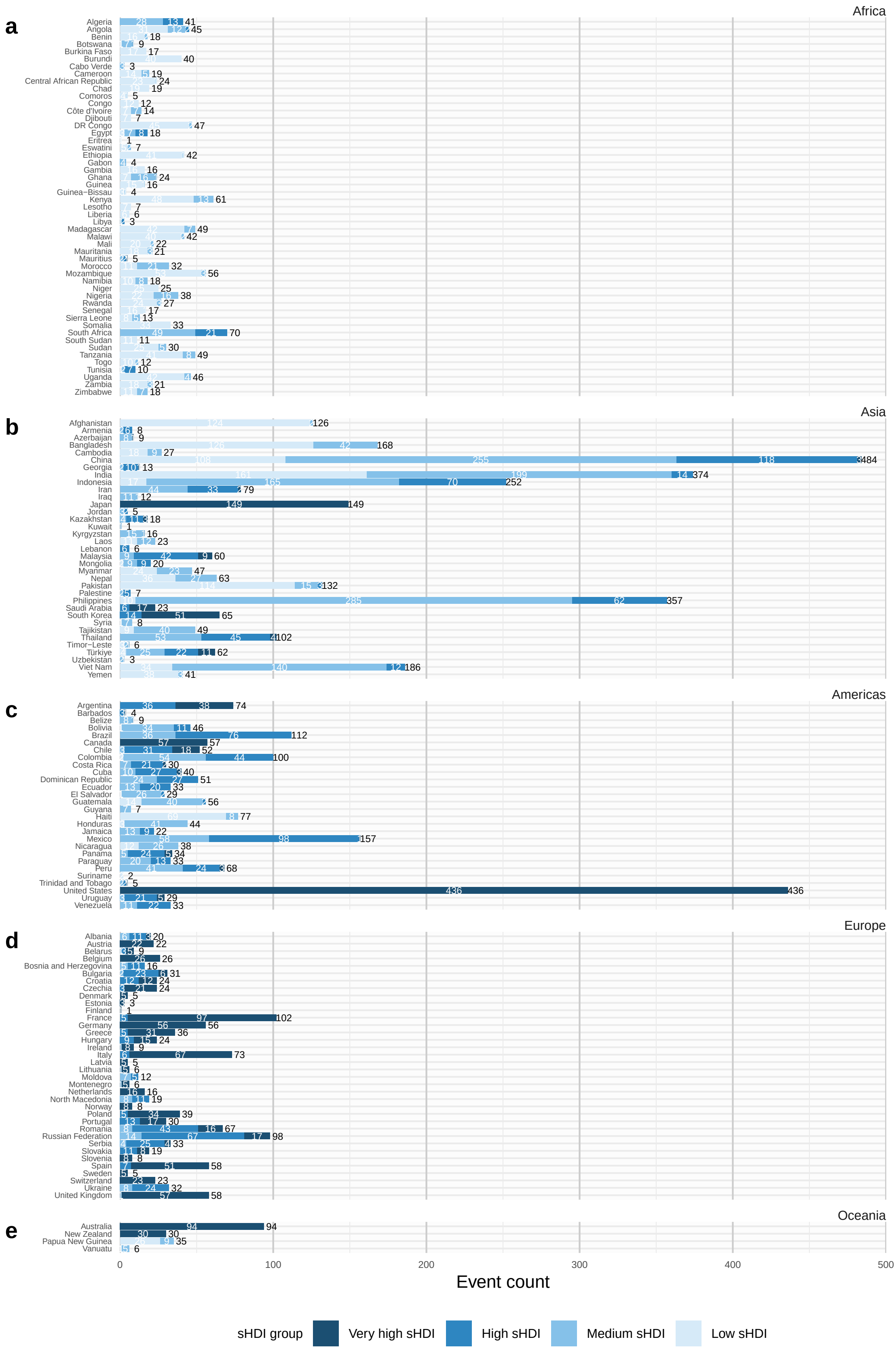

Supplementary Figure 1: Cumulative counts of reported disaster events from 1990 to 2020 per country and breakdown by subnational Human Development Index (sHDI) group of impacted regions. Panels are labeled as Africa (a), Asia (b), the Americas (c), Europe (d), and Oceania (e). For every continent, the barplot shows the number of reported events per country and the corresponding distribution of sHDI groups of the impacted regions. The five countries with highest numbers of reported events are: China, the United States, India, The Philippines and Indonesia. Two countries had reported regions from all sHDI groups: Türkiye and China, which were also among the countries with the largest increases in sHDI over the past 30 years.

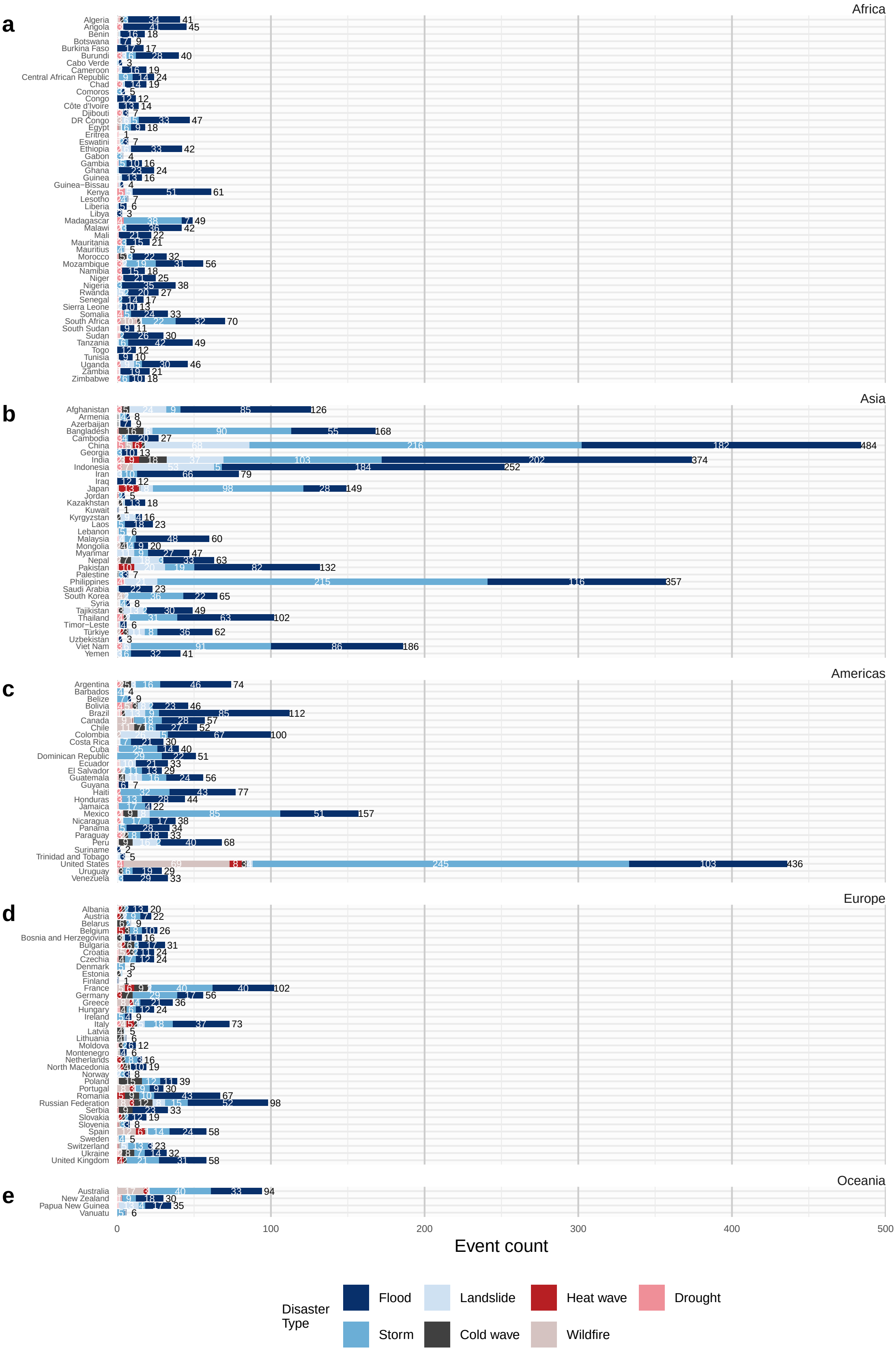

Supplementary Figure 2: Cumulative counts of reported disaster events from 1990 to 2020 per country and breakdown by disaster types. The barplot shows the count of reported events per country and the distribution of disaster types impacting each country. Panels are labeled as Africa (a), Asia (b), the Americas (c), Europe (d), and Oceania (e). Most reported wildfires occur in the United States. Floods are the most common disaster type in every continent, storms are predominantly reported in Asia and the Americas, and drought events in Europe are rare. Disaster types are color-coded: floods, storms, and landslides in shades of blue; heat waves, droughts, and wildfires in shades of red; cold waves in dark grey

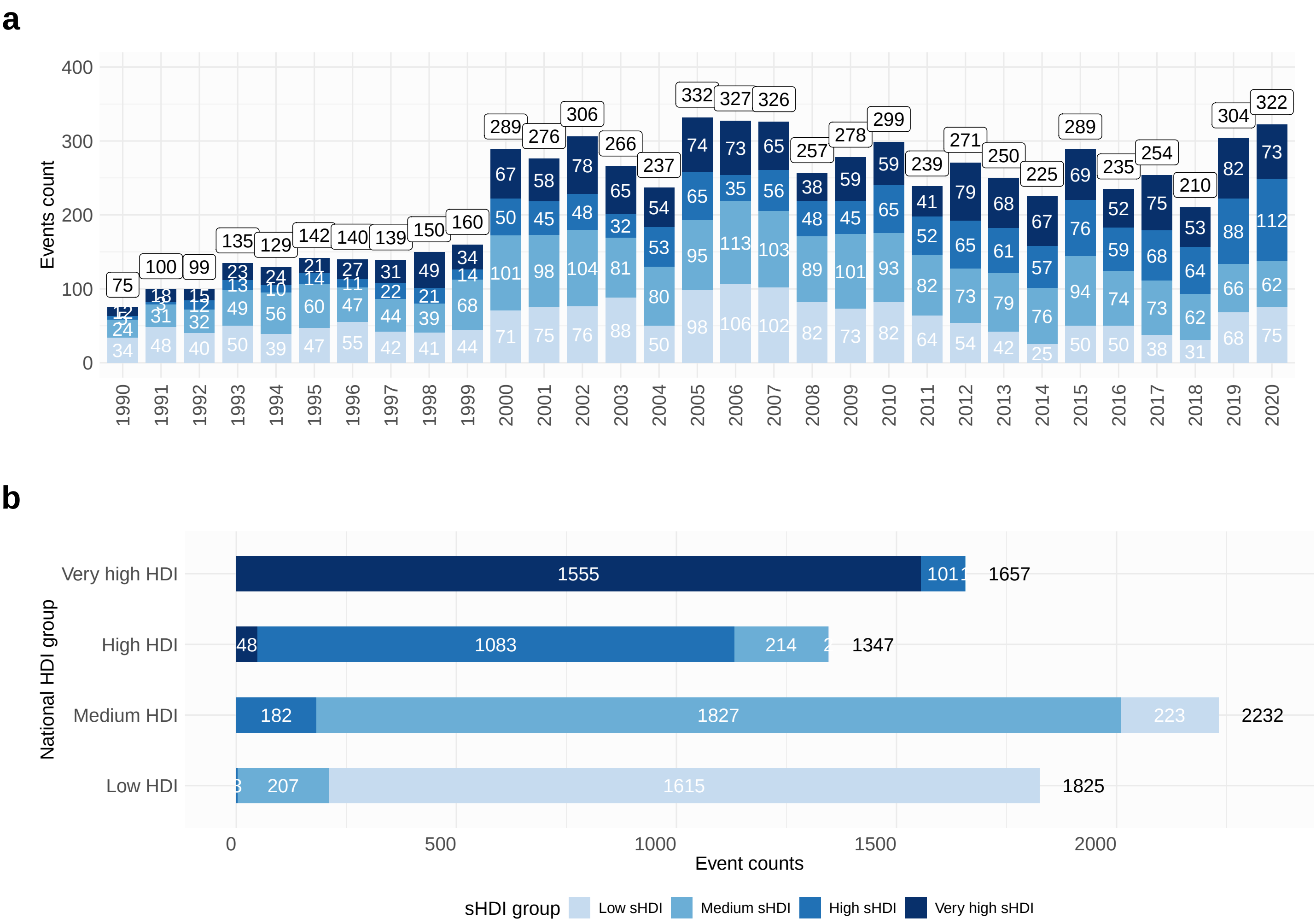

Supplementary Figure 3: Annual counts of climate-related disasters and mismatch between national Human Development Index (HDI) and subnational Human Development Index (sHDI) classifications in the impacted regions. (a) Annual counts of reported events between 1990 and 2020, and the breakdown per sHDI group. (b) Across all continents, approximately 13.56% of events occur in regions whose sHDI is different from the national HDI of the country at the time of impact, illustrating important within-country disparities that national-level metrics do not capture.

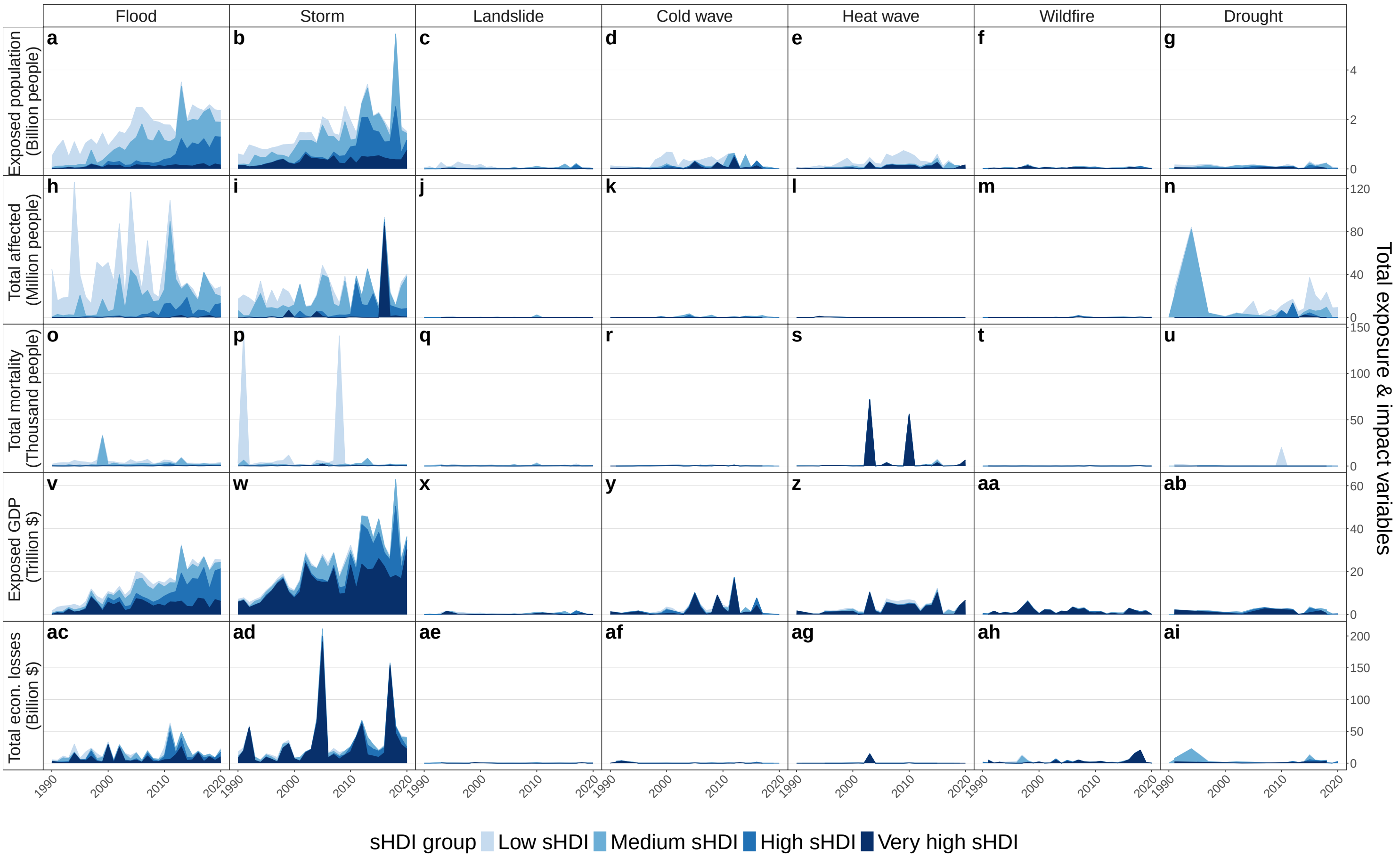

Supplementary Figure 4: Temporal evolution of exposure and disaster impacts from 1990 to 2020 by disaster type and subnational Human Development Index (sHDI) group. Annual sums of exposed population (billion people), affected people (million people), fatalities (thousand people), exposed gross domestic product (GDP; trillion US dollars), and total economic losses (billion US dollars) as stacked area charts, with shades of blue indicating sHDI from lightest (low) to darkest (very high). Panels are labelled (a—z, aa—ai) in reading order from left to right and top to bottom. Median trends are estimated using quantile regression ($\tau = 0.5$, the 0.5 quantile) and are indicated in Supplementary Tables 9–13 with the corresponding slopes and p-values.

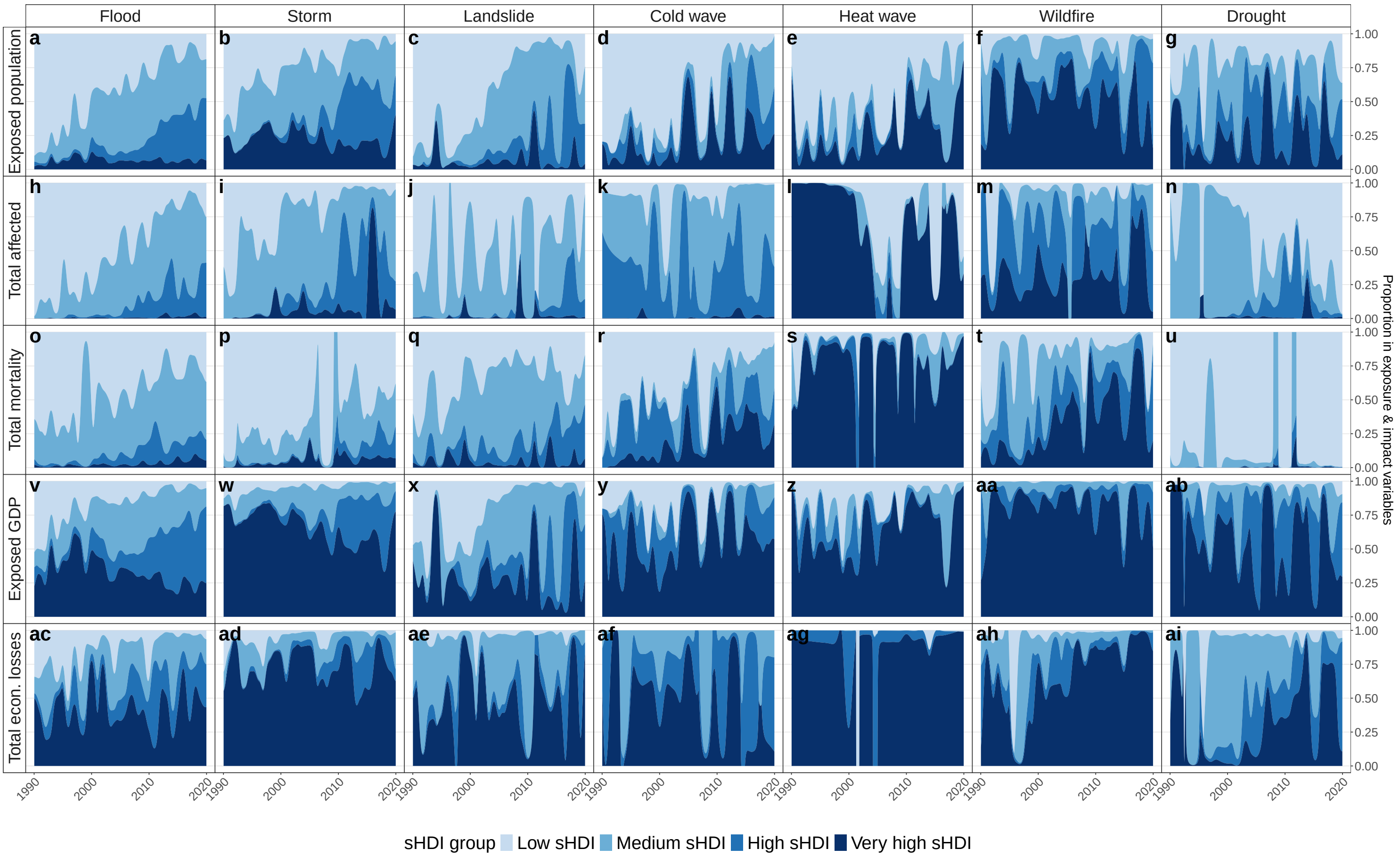

Supplementary Figure 5: Proportional exposure and disaster impacts from 1990 to 2020 by disaster type and subnational Human Development Index (sHDI) group. The figure shows the fractional contribution of each sHDI group to annual exposed population, affected people, fatalities, exposed gross domestic product (GDP), and total economic losses. Panels are labelled (a—z, aa—ai) in reading order from left to right and top to bottom. Shifts in these proportions reflect long-term socioeconomic transitions, including the decreasing global share of low-sHDI regions in population exposure and the increasing contributions from medium and high sHDI regions.

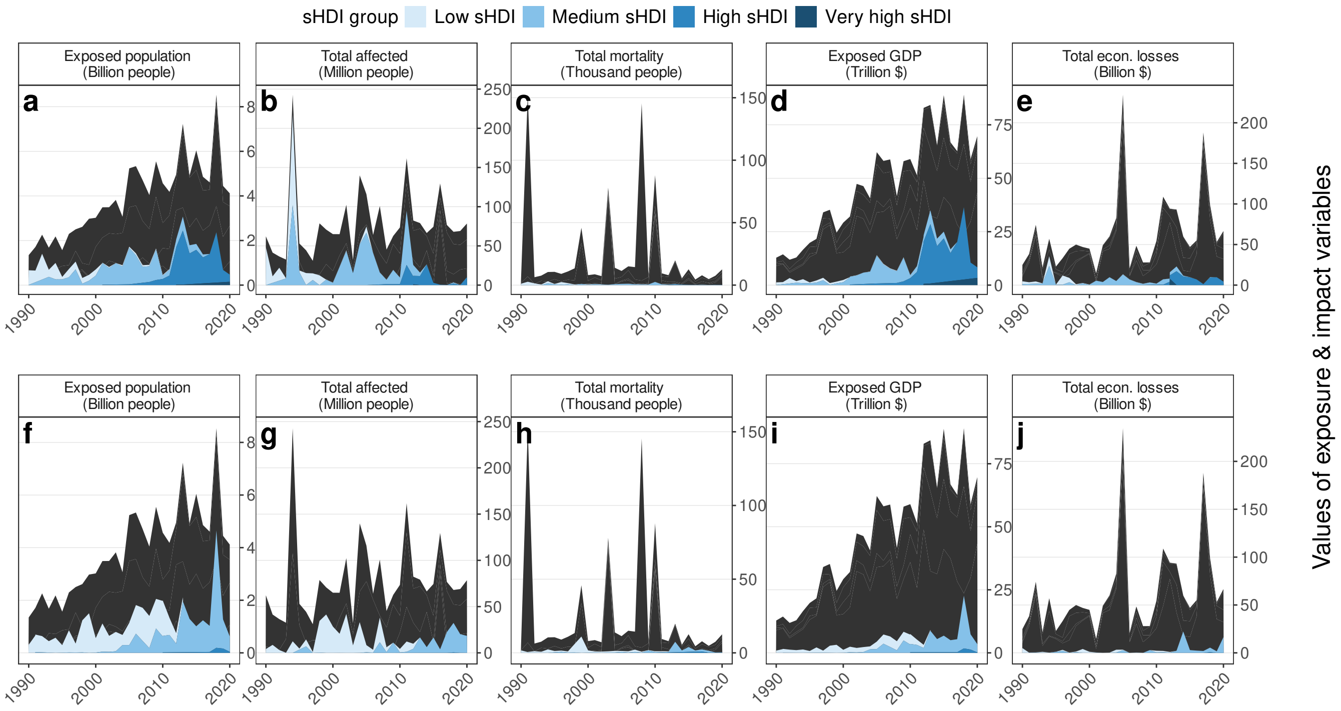

Supplementary Figure 6: Evolution of exposure and disaster impacts from 1990 to 2020 in China and India. Panels are labelled (a–j) in reading order from left to right and top to bottom, with the first row corresponding to China (a–e) and the second row to India (f–j). Panels show annual totals for exposed population (billion people), affected people (million people), fatalities (thousand people), exposed gross domestic product (GDP; trillion US dollars), and total economic losses (billion US dollars) for regions affected by climate-related disasters. Stacked areas represent contributions from subnational Human Development Index (sHDI) groups (low, medium, high, and very high, following United Nations Development Programme thresholds). Black shading indicates global annual totals for comparison. Exposure refers to population or GDP located in disaster-affected regions.

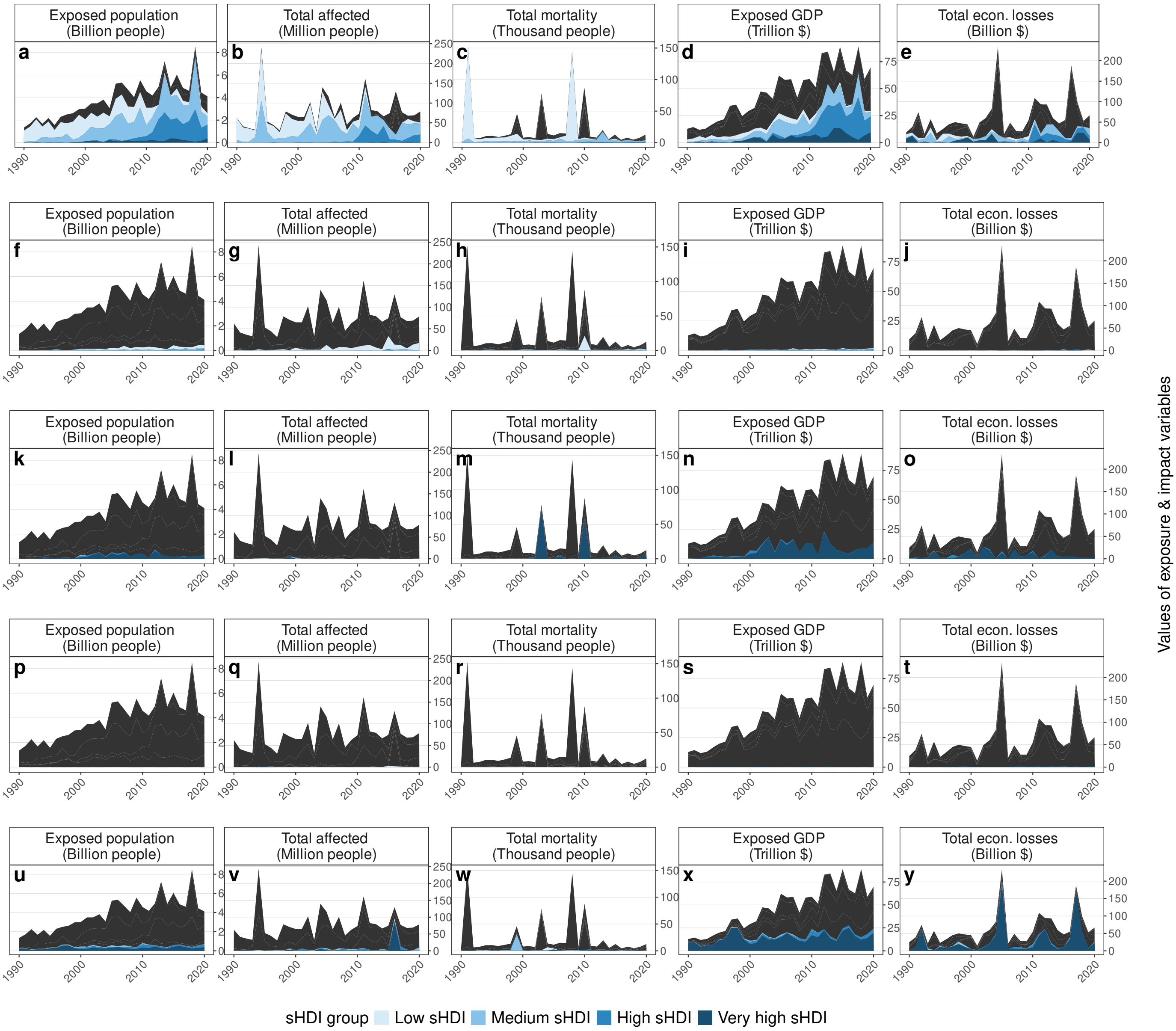

Supplementary Figure 7: Evolution of exposure and disaster impacts from 1990 to 2020 by continent. Panels are arranged by continent and labelled in reading order from left to right and top to bottom: Asia (a–e), Africa (f–j), Europe (k–o), Oceania (p–t), and the Americas (u–y). Within each continent, the five panels show annual totals of exposed population (billion people), affected people (million people), fatalities (thousand people), exposed gross domestic product (GDP; trillion US dollars), and total economic losses (billion US dollars), in that order, for regions impacted by climate-related disasters. Stacked areas represent contributions from subnational Human Development Index (sHDI) groups (low, medium, high, and very high, following United Nations Development Programme thresholds). Black shading indicates global annual totals for comparison. Exposure refers to population or GDP located in disaster-affected regions.

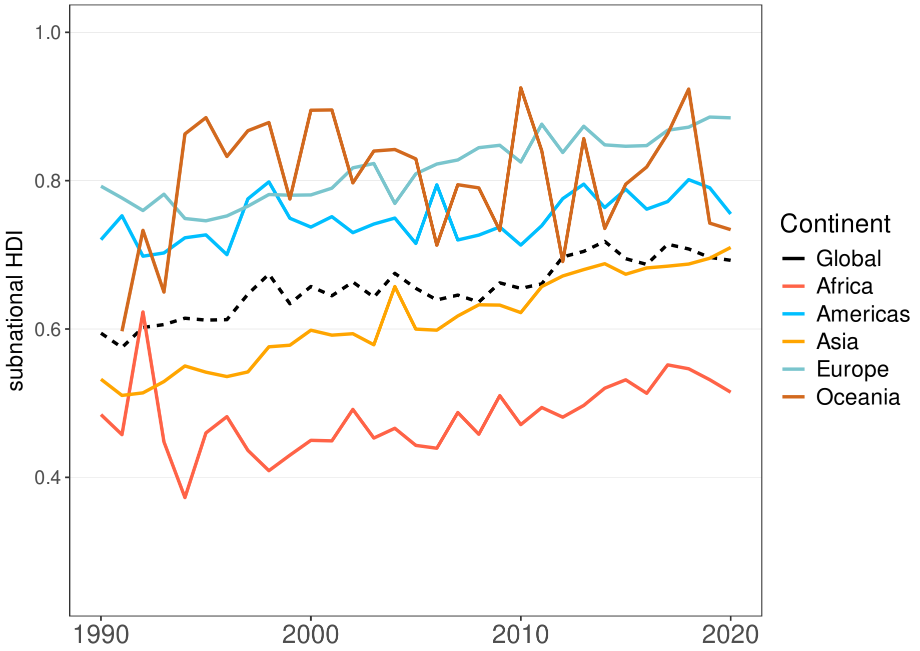

Supplementary Figure 8: Evolution of mean subnational Human Development Index (sHDI) at the continental level from 1990 to 2020. The lines show the evolution of the average sHDI of impacted regions, aggregated by continent. These trajectories illustrate patterns of socioeconomic development across continents, with all continents except Africa showing convergence towards higher sHDI levels over time. The important variability in Oceania's mean sHDI is explained by the small number of countries constituting this group and their contrasting development levels: two highly developed countries (AUS, Australia; NZL, New Zealand), and two substantially less developed countries (PNG, Papua New Guinea; VUT, Vanuatu).

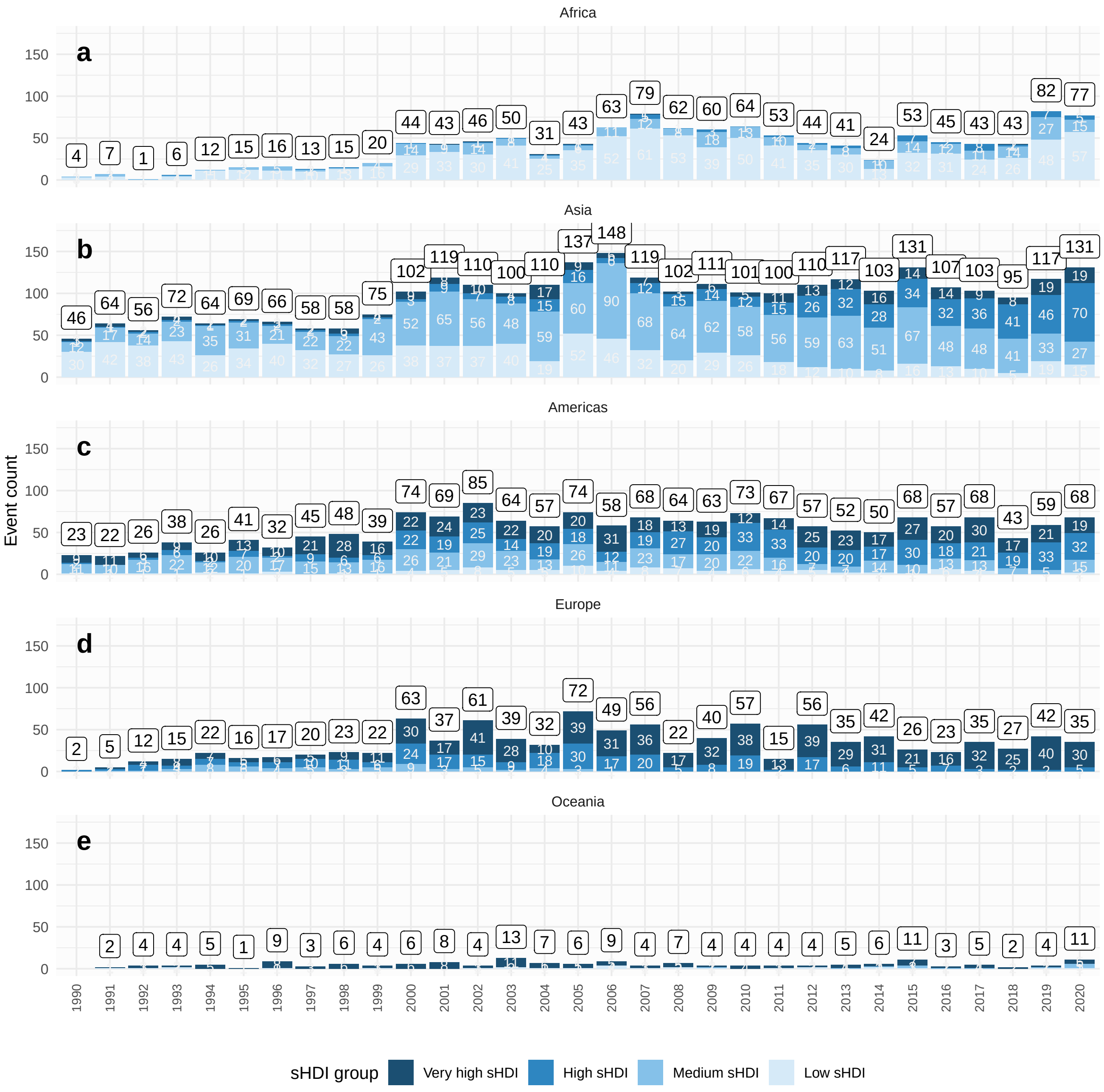

Supplementary Figure 9: Global Annual counts of climate-related disasters by continent and subnational Human Development Index (sHDI) group from 1990 to 2020. Panels are labeled as Africa (a), Asia (b), the Americas (c), Europe (d), and Oceania (e). The panels show the annual count of climate-related disasters disaggregated by continent. Over the past two decades, Asia recorded the highest absolute number of events, followed by the Americas. While low and medium sHDI regions account for 59% and 36%, respectively, of all reported impacts during 1990–2020, the largest increases in exposure and reported impacts over time originate from medium, high, and very high sHDI regions, reflecting global socioeconomic transitions and shifting reporting patterns.

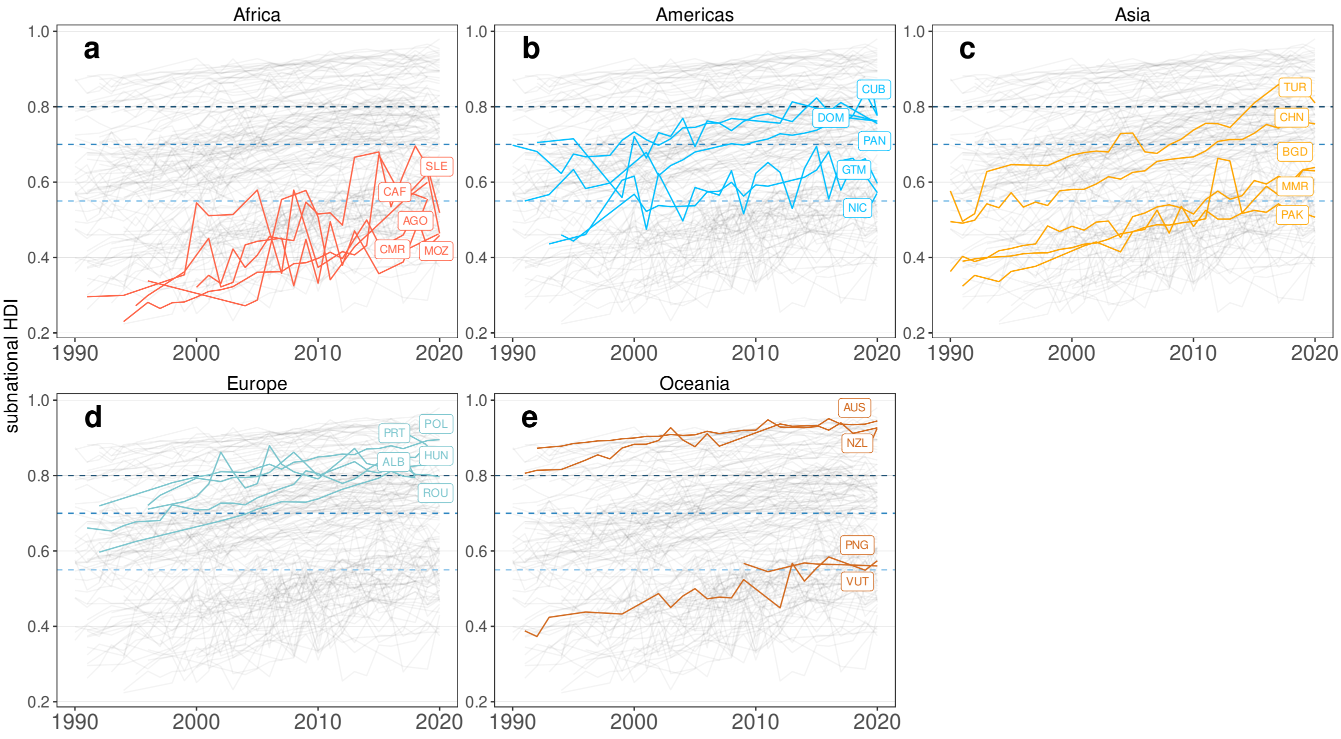

Supplementary Figure 10: Evolution of mean subnational human development index (sHDI) at country level from 1990 to 2020. The lines show the evolution of average sHDI of reported impacted regions aggregated at country level per continent (panel). Panels are labeled as Africa (a), the Americas (b), Asia (c), Europe (d), and Oceania (e). For every continent, we show the 5 countries with highest mean sHDI increase between 1990 and 2020. The different countries take distinct socioeconomic development paths and promoting from one sHDI group to the next is the case in the Americas, Asia and Europe. In Africa, the average sHDI at 2020 for the countries with the highest evolution still lies in low sHDI (< 0.55). In Oceania, the four countries that make this group are shown. The grey lines in the background represent all other countries. Horizontal dashed lines indicate the thresholds separating the four sHDI groups (low, medium, high, and very high). The highlighted country names corresponding to iso codes are: In Africa: AGO (Angola), CAF (Central African Republic), CMR (Cameroon), MOZ (Mozambique), SLE (Sierra Leone). In the Americas: CUB (Cuba), DOM (Dominican Republic), GTM (Guatemala), NIC (Nicaragua), PAN (Panama). In ASIA: BGD (Bangladesh), CHN (China), MMR (Myanmar), PAK (Pakistan), TUR (Türkiye). In Europe: ALB (Albania), HUN (Hungary), POL (Poland), PRT (Portugal), PRT (Azores Islands), ROU (Romania). In Oceania: AUS (Australia), NZL (New Zealand), PNG (Papua New Guinea), VUT (Vanuatu).

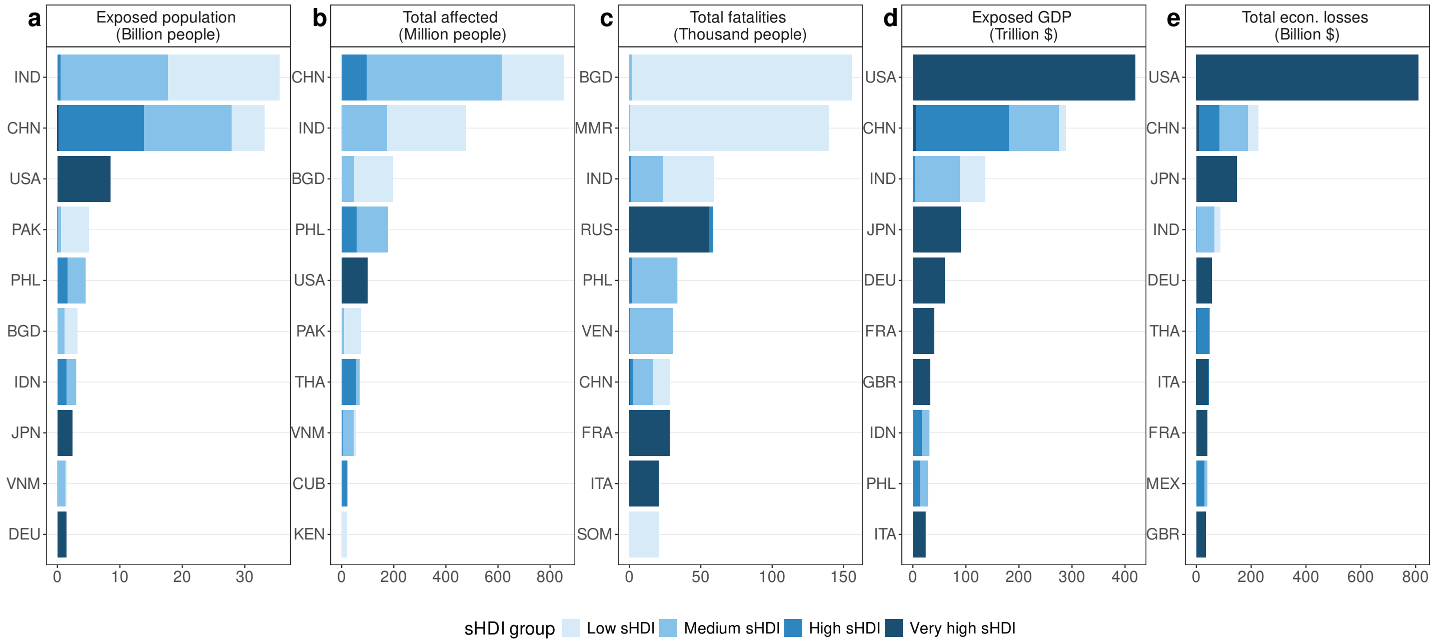

Supplementary Figure 11: Most exposed and most impacted countries between 1990 and 2020. The panels highlight the ten countries with the highest exposed population (a), total affected people (b), total fatalities (c), exposed gross domestic product (d), and total economic losses (e). These countries collectively account for a disproportionately large share of global exposure and impacts. The proportion per sHDI group within each country is indicated with the stacked bars. The country names corresponding to iso codes are: AUS (Australia), BGD (Bangladesh), BRA (Brazil), CHN (China), CUB (Cuba), DEU (Germany), ETH (Ethiopia), FRA (France), GBR (United Kingdom), IDN (Indonesia), IND (India), ITA (Italy), JPN (Japan), MEX (Mexico), MMR (Myanmar), PAK (Pakistan), PHL (Philippines), RUS (Russian Federation), SOM (Somalia), THA (Thailand), USA (United States of America), VEN (Venezuela), VNM (Viet Nam).

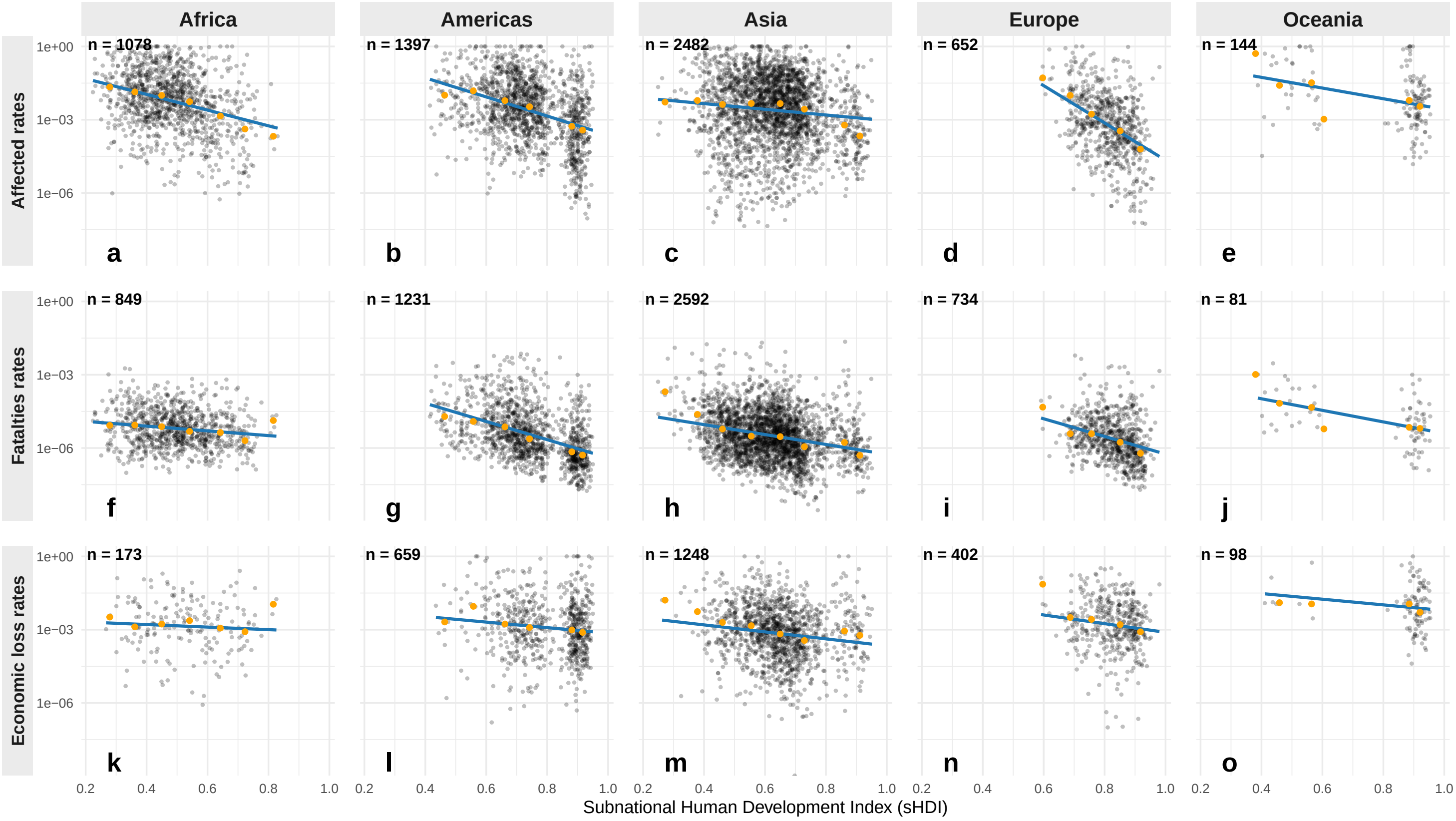

Supplementary Figure 12: Relationship between subnational Human Development Index (sHDI) and disaster impact rates across continents (1990–2020). Panels are arranged by impact type and continent, and labelled in reading order from left to right and top to bottom: affected-population rates (a–e), fatality rates (f–j), and economic-loss rates (k–o), each shown for Africa (a, f, k), the Americas (b, g, l), Asia (c, h, m), Europe (d, i, n), and Oceania (e, j, o). Each panel shows impact rates (normalized affected-population rates, fatality rates, and economic-loss rates) as functions of sHDI for disaster-impacted regions. Regions are grouped by continent, with clusters illustrating how impact severity decreases with increasing sHDI, but with substantial intra-continental heterogeneity. In three continents, we see a divide between a cluster of highly developed regions (sHDI ≥ 0.80) and the rest—formed by Australia and New Zealand in Oceania; Japan, South Korea, Saudi Arabia, and Türkiye in Asia; and the United States, Canada, Chile, and Argentina in the Americas. In every panel, the smoothed line corresponds to the linear regression fit. The orange points represent the median impact rates within sHDI bins of width 0.1. The impacts are shown on log10 scale on the y-axis.

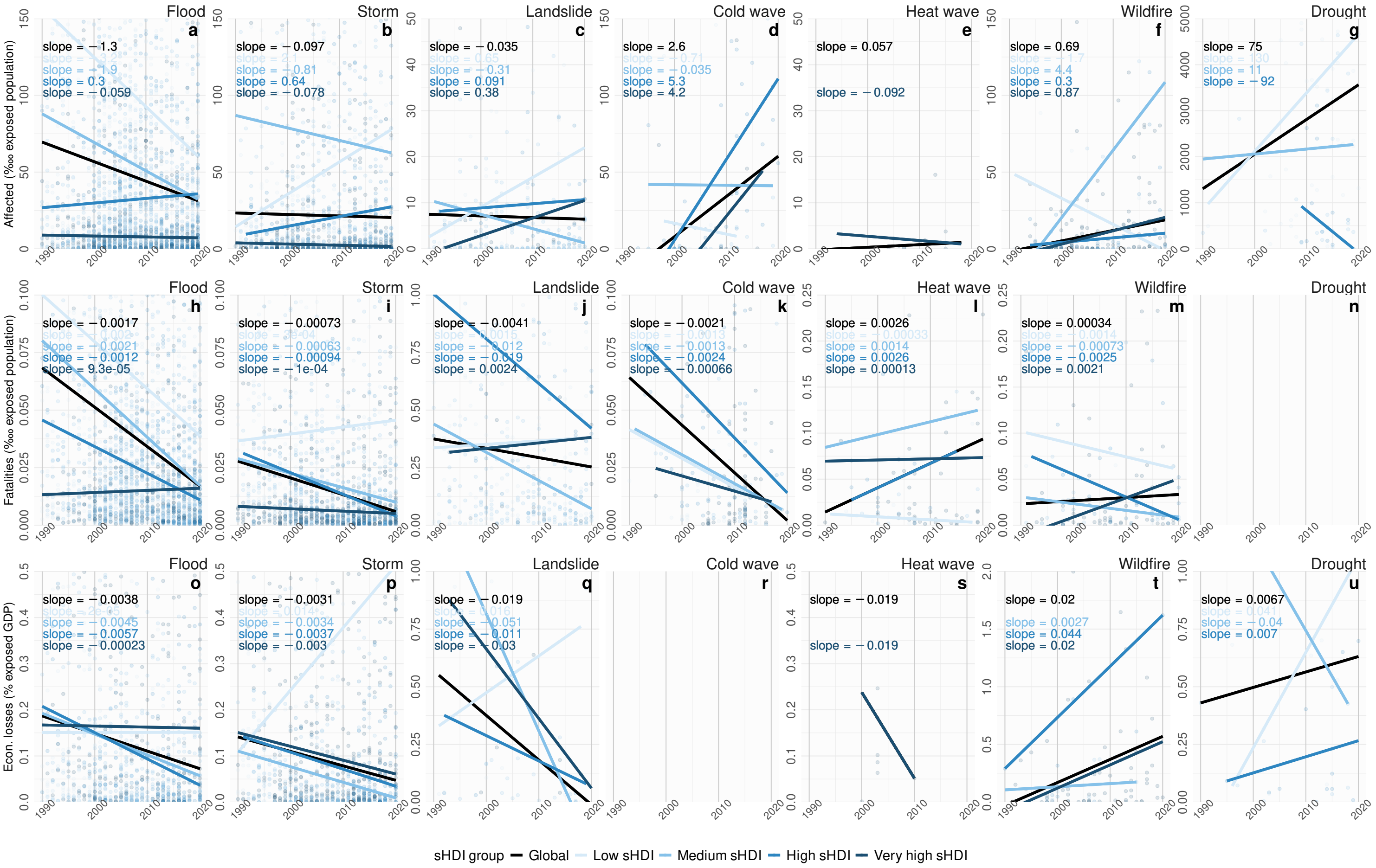

Supplementary Figure 13: Median trends of event-level impact rates of human (affected and fatalities) and economic losses per disaster type from 1990 to 2020. Panels are arranged by impact type and disaster type, and labelled in reading order from left to right and top to bottom: affected-population rates (a–g), fatality rates (h–n), and economic-loss rates (o–u), each shown for flood, storm, landslide, cold wave, heat wave, wildfire, and drought. For each disaster type and each sHDI group, we estimated trends using quantile regression ($\tau = 0.5$), the corresponding slopes and p-values are indicated on the figure and in Supplementary Tables 13–15. The black lines indicates global trends. Trend lines with less than 10 observations were not considered. For human losses, impact rates are defined as the number of affected individuals or fatalities per 10,000 exposed population. For economic losses, impact rates are defined as reported damages expressed as a percentage of exposed gross domestic product (GDP).

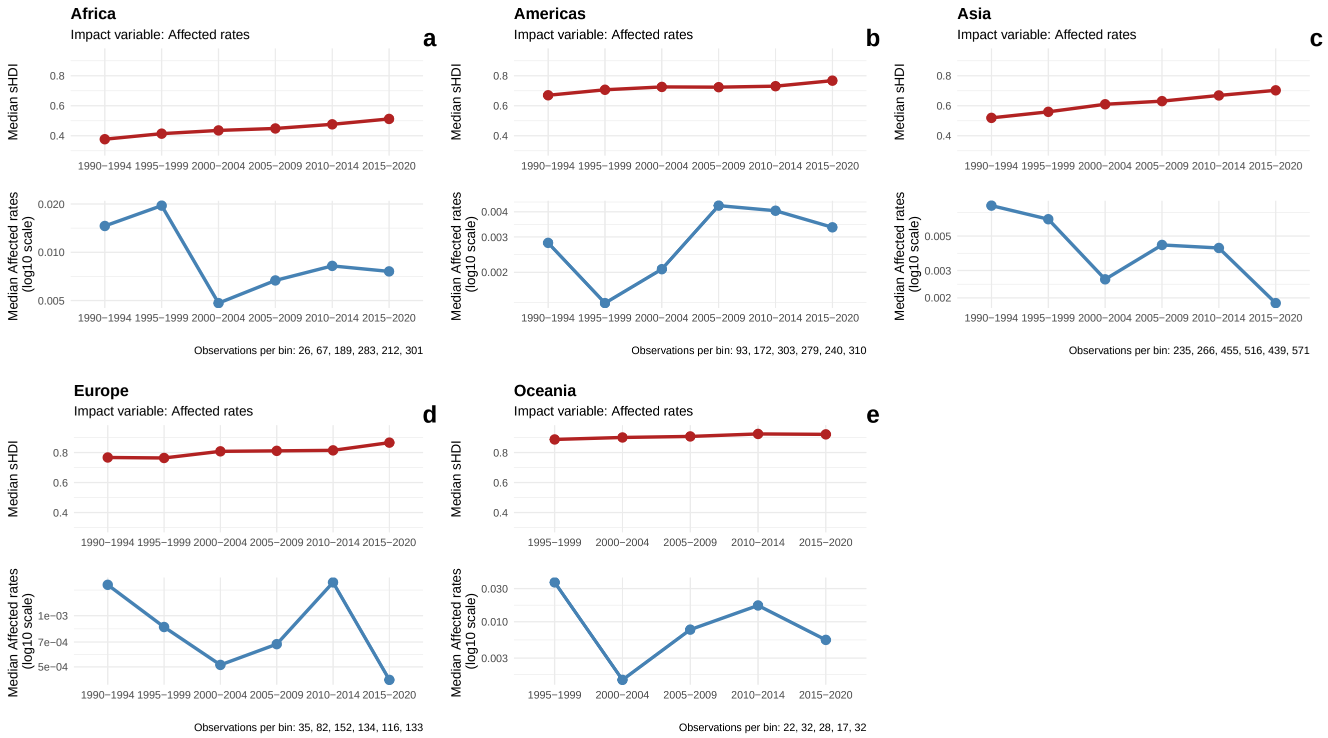

Supplementary Figure 14: Temporal co-evolution of median subnational Human Development Index (sHDI) and affected rates across continents from 1990 to 2020. Panels are labeled in reading order from left to right as Africa (a), the Americas (b), Asia (c), Europe (d), and Oceania (e). Median sHDI and median affected rates are computed over 5-year time bins for each continent (bins with fewer than 20 events excluded). Within each panel, the upper subpanel shows long-term evolution in sHDI, while the lower subpanel shows corresponding changes in affected rates (log10 scale). Affected rates are defined as the number of affected people per exposed population. Event counts per bin are reported below each lower subpanel.

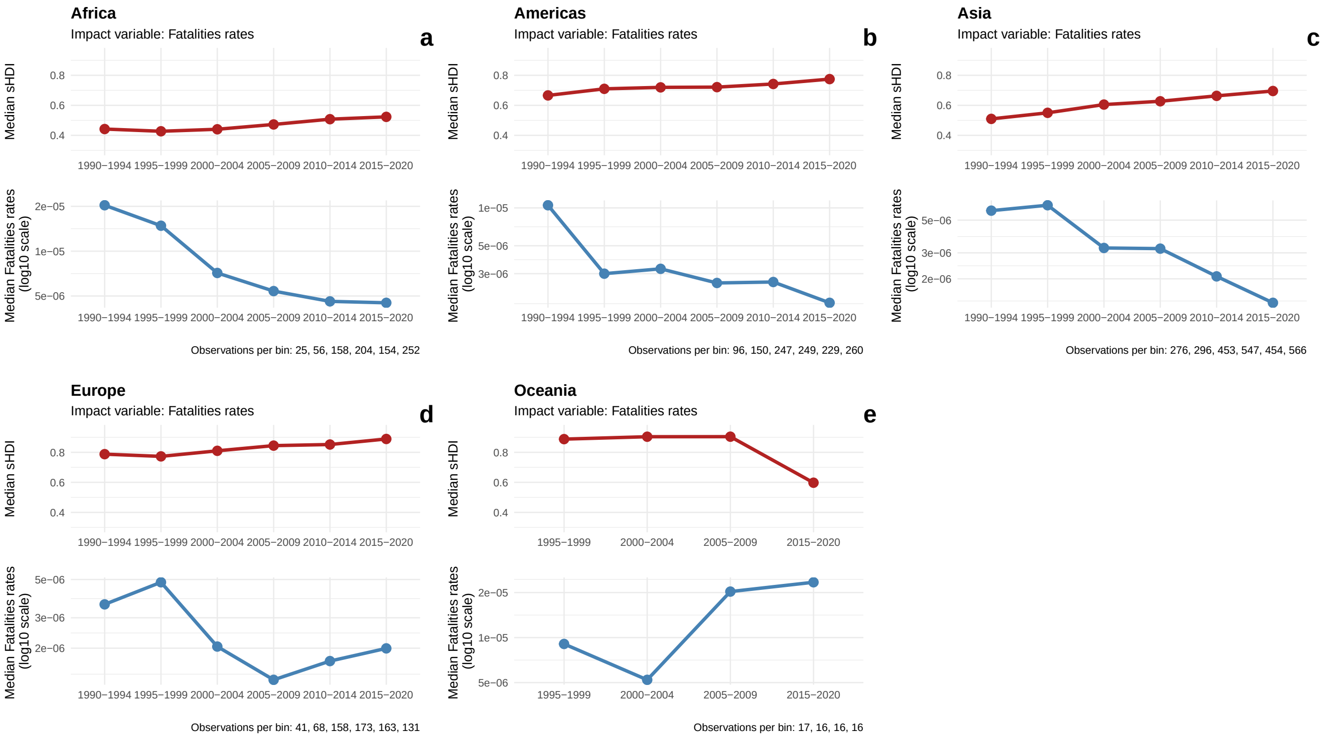

Supplementary Figure 15: Temporal co-evolution of median subnational Human Development Index (sHDI) and fatality rates across continents from 1990 to 2020. Panels are labeled in reading order from left to right as Africa (a), the Americas (b), Asia (c), Europe (d), and Oceania (e). Median sHDI and median fatality rates are computed over 5-year time bins for each continent (bins with fewer than 20 events excluded). Within each panel, the upper subpanel shows long-term evolution in sHDI, while the lower subpanel shows corresponding changes in fatality rates (log10 scale). Fatality rates are defined as the number of fatalities per exposed population. Event counts per bin are reported below each lower subpanel. The apparent decline in median sHDI for Oceania during 2015-2020 reflects the composition of events with available fatality data in recent years, which are predominantly from lower-development countries (Papua New Guinea and Vanuatu) and therefore disproportionately influence the regional median.

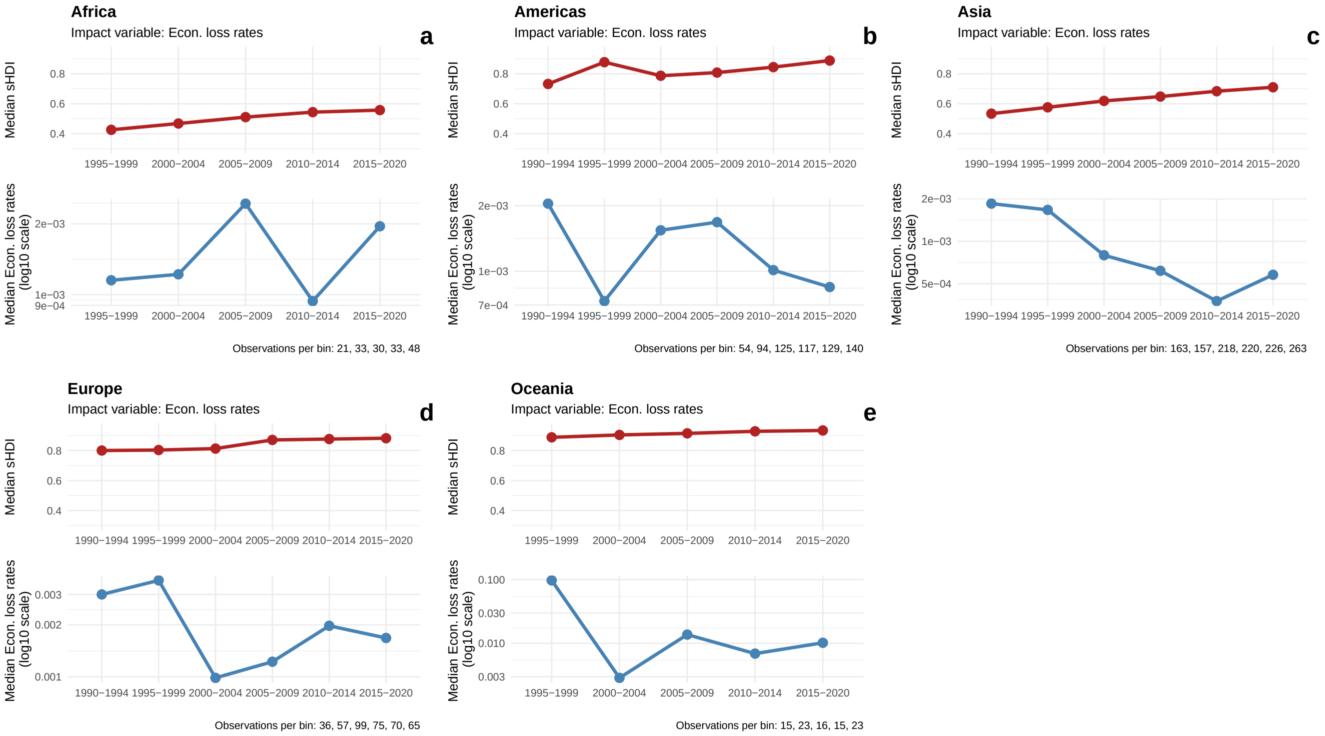

Supplementary Figure 16: Temporal co-evolution of median subnational Human Development Index (sHDI) and economic loss rates across continents from 1990 to 2020. Panels are labeled in reading order from left to right as Africa (a), the Americas (b), Asia (c), Europe (d), and Oceania (e). Median sHDI and median economic loss rates are computed over 5-year time bins for each continent (bins with fewer than 20 events excluded). Within each panel, the upper subpanel shows long-term evolution in sHDI, while the lower subpanel shows corresponding changes in economic loss rates (log10 scale). Economic loss rates are defined as reported damages expressed as a fraction of exposed gross domestic product (GDP). Event counts per bin are reported below each lower subpanel. Temporal patterns show increased variability, reflecting both the sparse and heterogeneous reporting of economic loss data in EM-DAT and the heavy-tailed distribution of disaster-related economic losses.

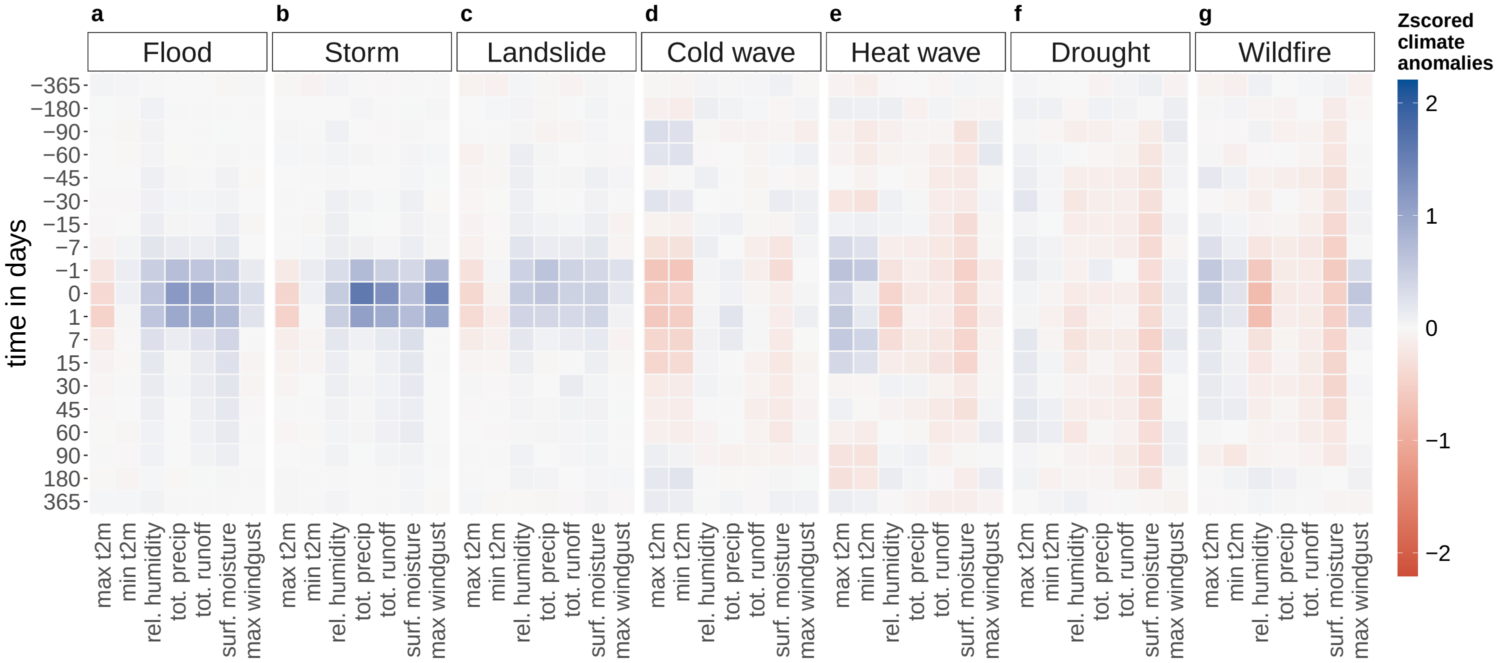

Supplementary Figure 17: Composite climatological anomalies stratified by disaster type. Panels are labeled as Flood (a), Storm (b), Landslide (c), Cold wave (d), Heat wave (e), Drought (f), and Wildfire (g). Average climate anomalies per disaster type at selected dates between one year before and after the reported start of the events. The Composite climatological anomalies were calculated using the superposed epoch analysis (see methods). These anomalies show the temporal evolution of the averaged daily anomalies. All climate variables (x-axis) are standardized (zero mean, unit variance) for comparability. Color indicates the z-scored anomaly magnitude, with red denoting positive anomalies and blue denoting negative anomalies. Climate variables: maximum temperature at 2 metres (max t2m), minimum temperature at 2 metres (min t2m), relative humidity (rel. humidity), total precipitation (tot. precip), total runoff (tot. runoff), surface moisture (surf. moisture), and maximum wind gust (max windgust).

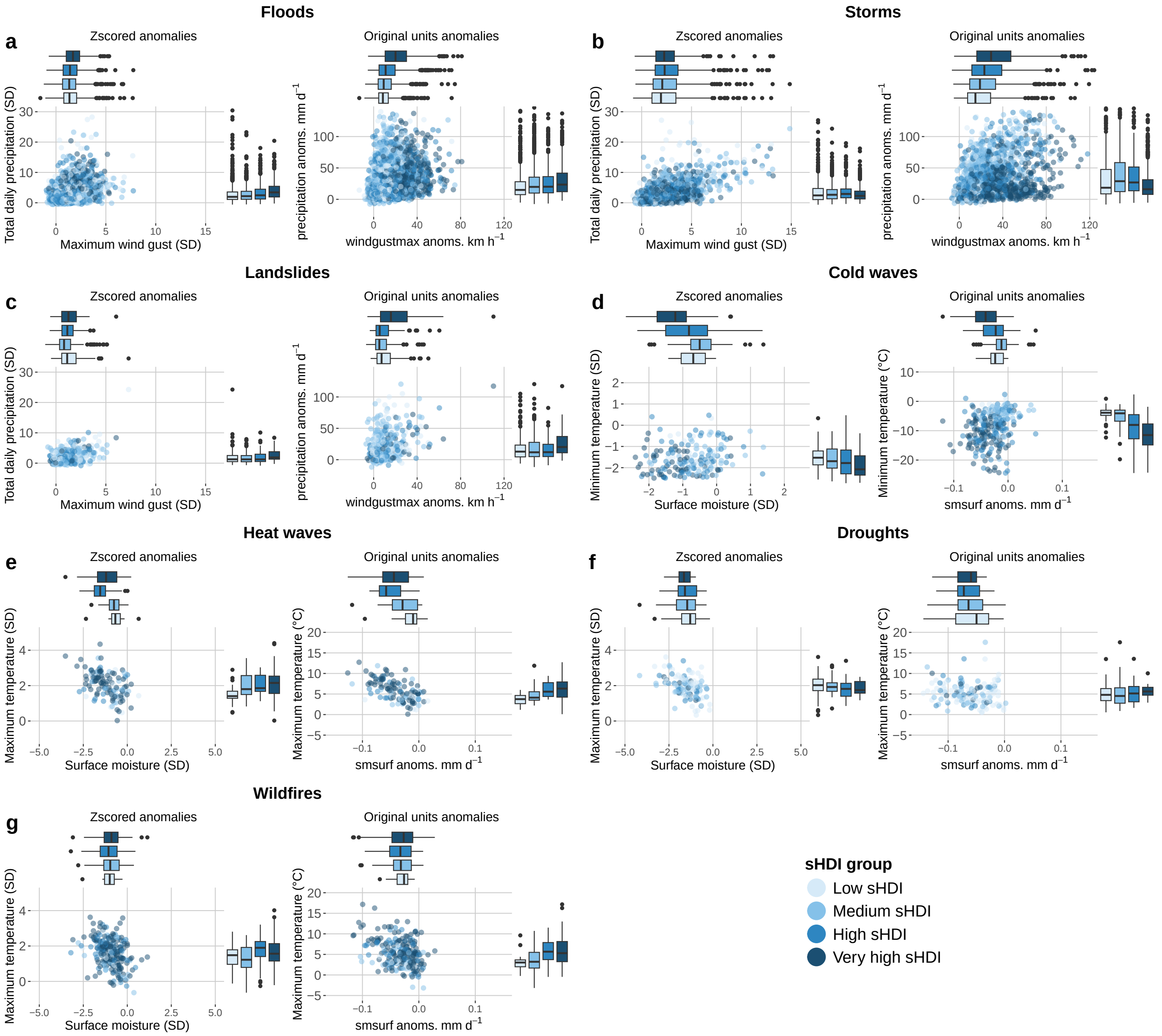

Supplementary Figure 18: Bivariate distributions of climate anomalies stratified by subnational Human Development Index (sHDI) and by disaster type. Panels are labelled in reading order from left to right as floods (a), storms (b), landslides (c), cold waves (d), heat waves (e), droughts (f), and wildfires (g). For each disaster type, the left subpanel shows z-scored anomalies and the right subpanel shows anomalies in original units. The scatter plots show, for every disaster type, the bivariate distribution of climatological anomalies in both original units and z-scored normalization. Original anomalies represent the absolute magnitude of the underlying climate conditions, whereas z-scored anomalies indicate the severity of each event relative to its local climatology. The marginal boxplots show both aspects of severity per sHDI group. For floods, storms and landslides, the considered variables are total daily precipitation (y-axis) and maximum daily wind gust (x-axis). For cold waves, the considered variables are minimum daily temperature (y-axis) and daily surface moisture (x-axis). For heat waves, droughts and wildfires, the considered variables are maximum daily temperature (y-axis) and daily surface moisture (x-axis). For each disaster event, the corresponding climate variables were sampled within a $\pm$ 3-day window around the reported event start date. The box plots show the median (center line), interquartile range (box limits, 25th–75th percentiles), 1.5$\times$ interquartile range (whiskers), and outliers (individual points beyond the whiskers).

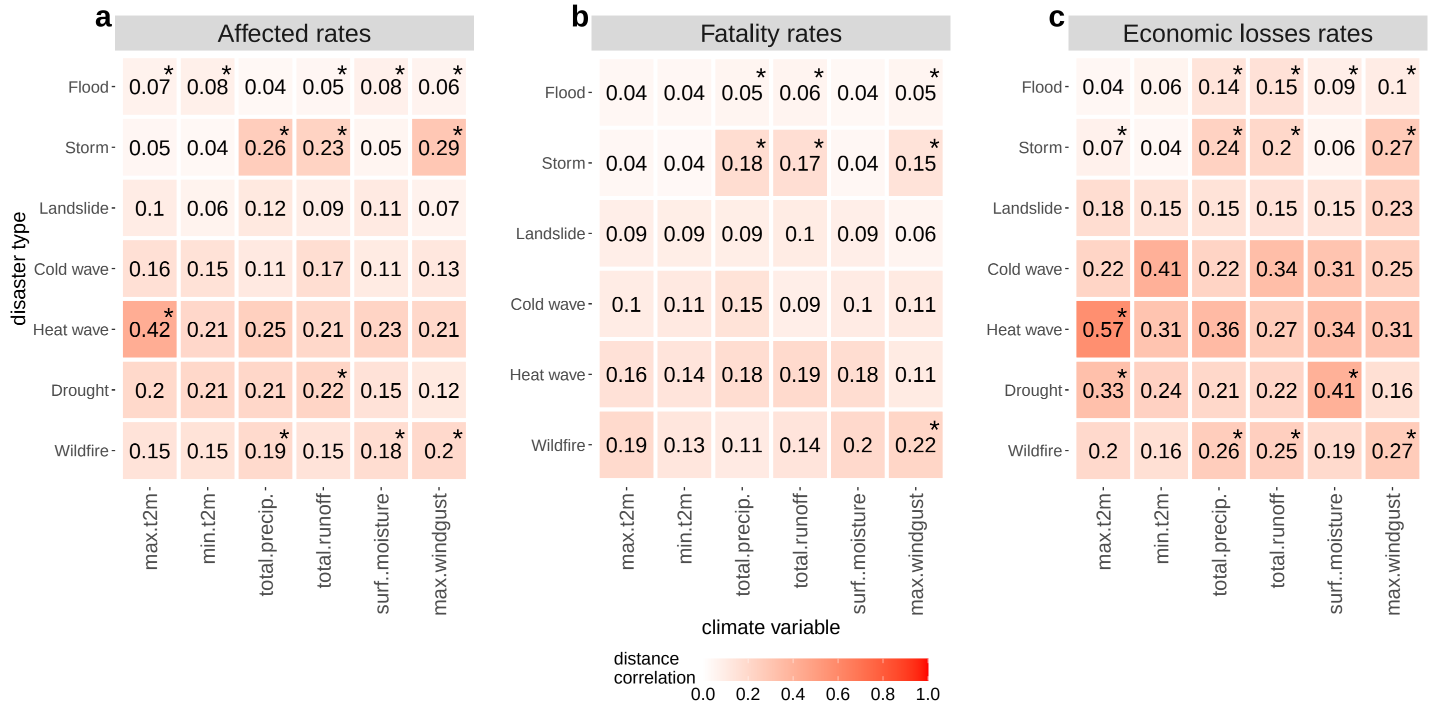

Supplementary Figure 19: Correlation between impact rates and climate anomalies. Distance correlation between the climate anomalies (x-axis) and the impact rates for affected (a), fatalities (b), and economic losses (c) for each disaster type (y-axis). Statistical significance ($p < 0.05$) is determined with permutation testing and indicated with asterisk in case of significance. Climate variables: maximum temperature at 2 metres (max t2m), minimum temperature at 2 metres (min t2m), total precipitation (tot. precip), total runoff (tot. runoff), surface moisture (surf. moisture), and maximum wind gust (max windgust).

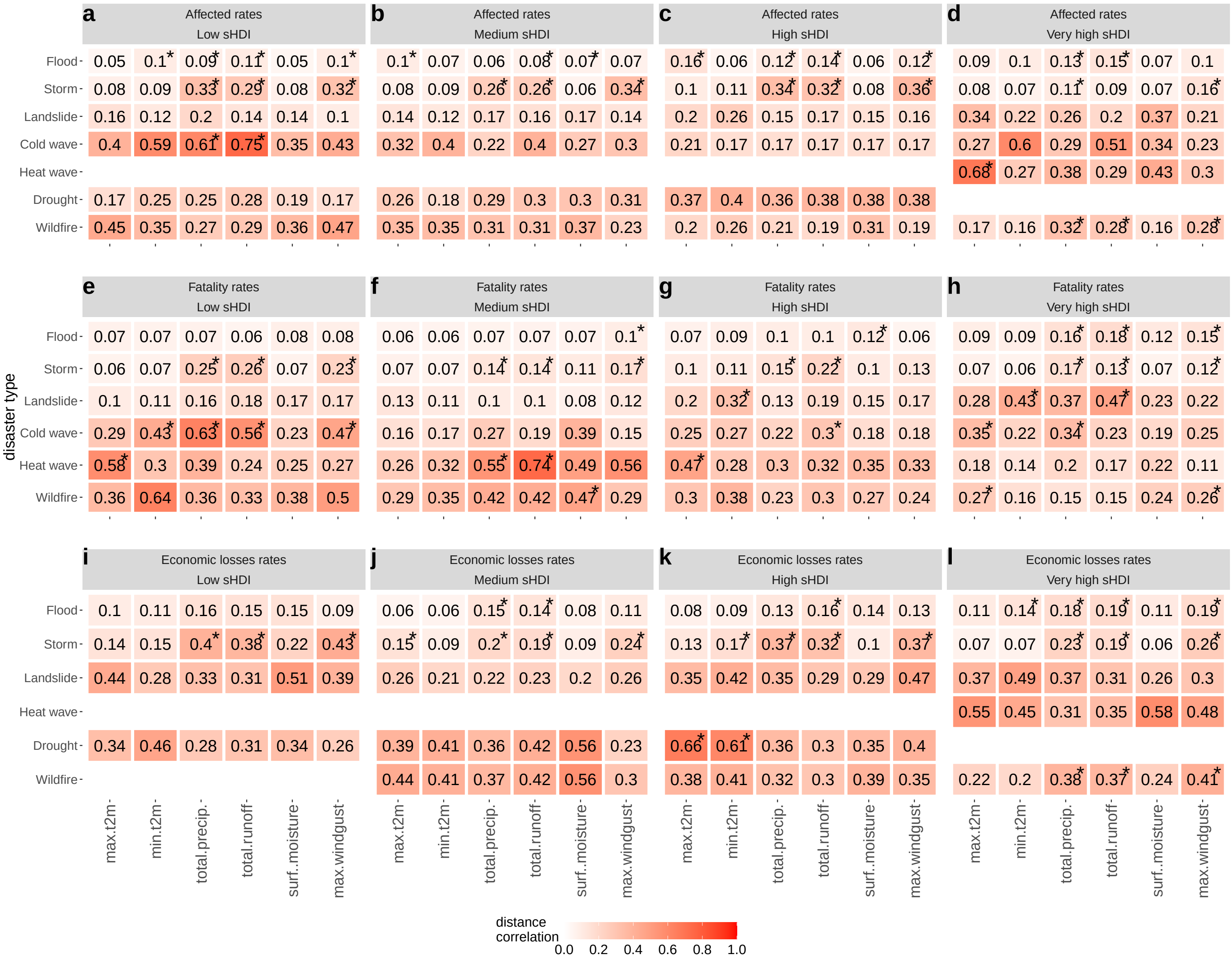

Supplementary Figure 20: Correlation between impact rates and climate anomalies stratified by subnational human development index (sHDI) group. Distance correlation between the climate anomalies (x-axis) and impact rates for affected (a–d), fatalities (e–h), and economic losses (i–l) for each disaster type (y-axis). Within each row, panels are ordered from left to right by sHDI group: low (a, e, i), medium (b, f, j), high (c, g, k), and very high (d, h, l). Statistical significance ($p < 0.05$) is determined with permutation testing and indicated with asterisk in case of significance. The climate anomalies (x-axis) are as follow: maximum temperature, minimum temperature, total precipitation, surface moisture, total runoff and maximum wind gust.

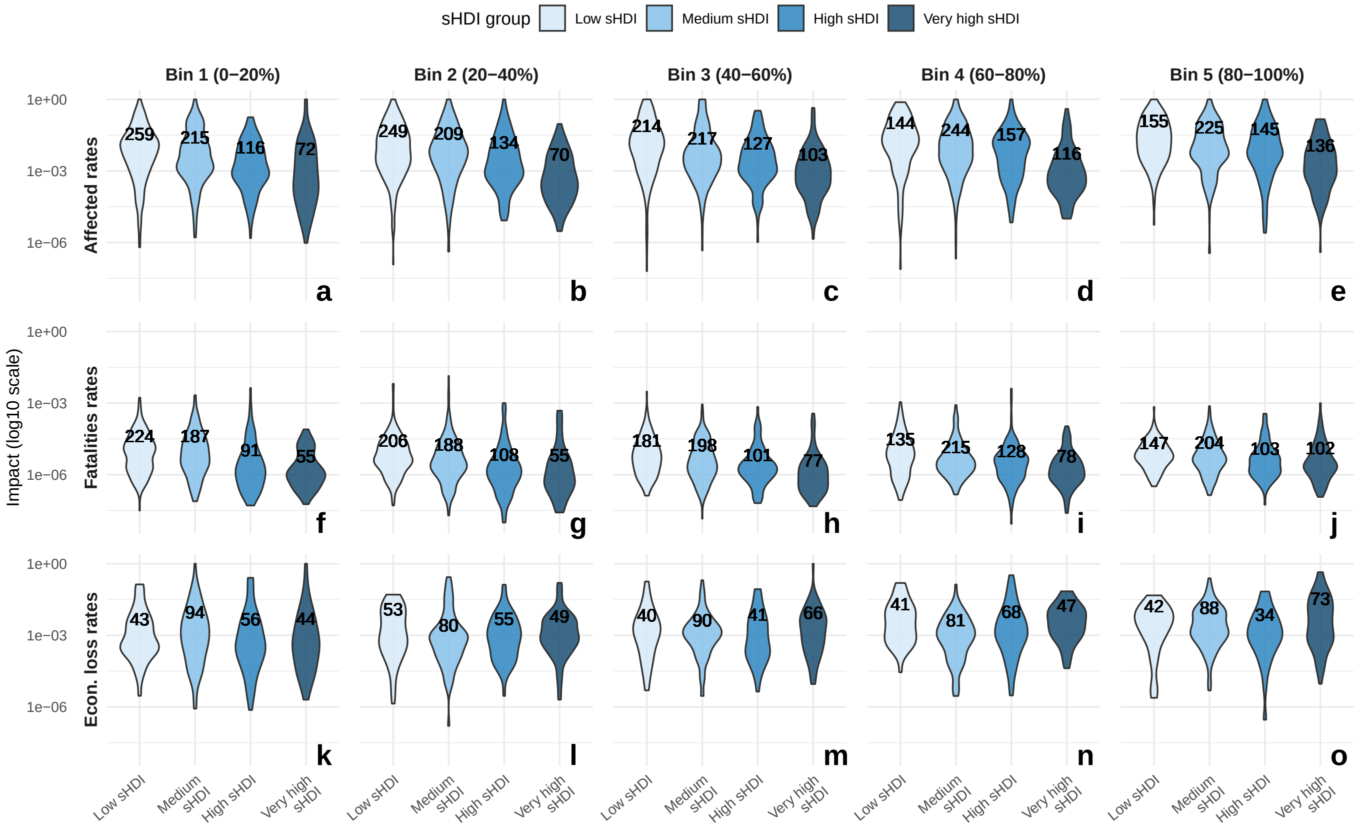

Supplementary Figure 21: Hazard intensity stratification of flood impacts per subnational Human Development Index (sHDI) group. Flood events are partitioned into quintiles of standardized intensity (0–20%, 20–40%, 40–60%, 60–80%, 80–100%) based on maximum daily precipitation anomalies. Panels are labeled from left to right and top to bottom as (a–e) affected rates, (f–j) fatality rates, and (k–o) economic loss rates, with columns corresponding to increasing intensity bins. Violin plots show distributions of affected rates, fatality rates, and economic loss rates (log10 scale) for each sHDI group; labels denote the number of events retained after applying an $n \geq 20$ threshold. The separation between development groups persists across all intensity strata and impact dimensions, suggesting that differences in impacts are not solely explained by hazard intensity. Violin plots show the full distribution of the impact rates, with width proportional to the estimated probability density.

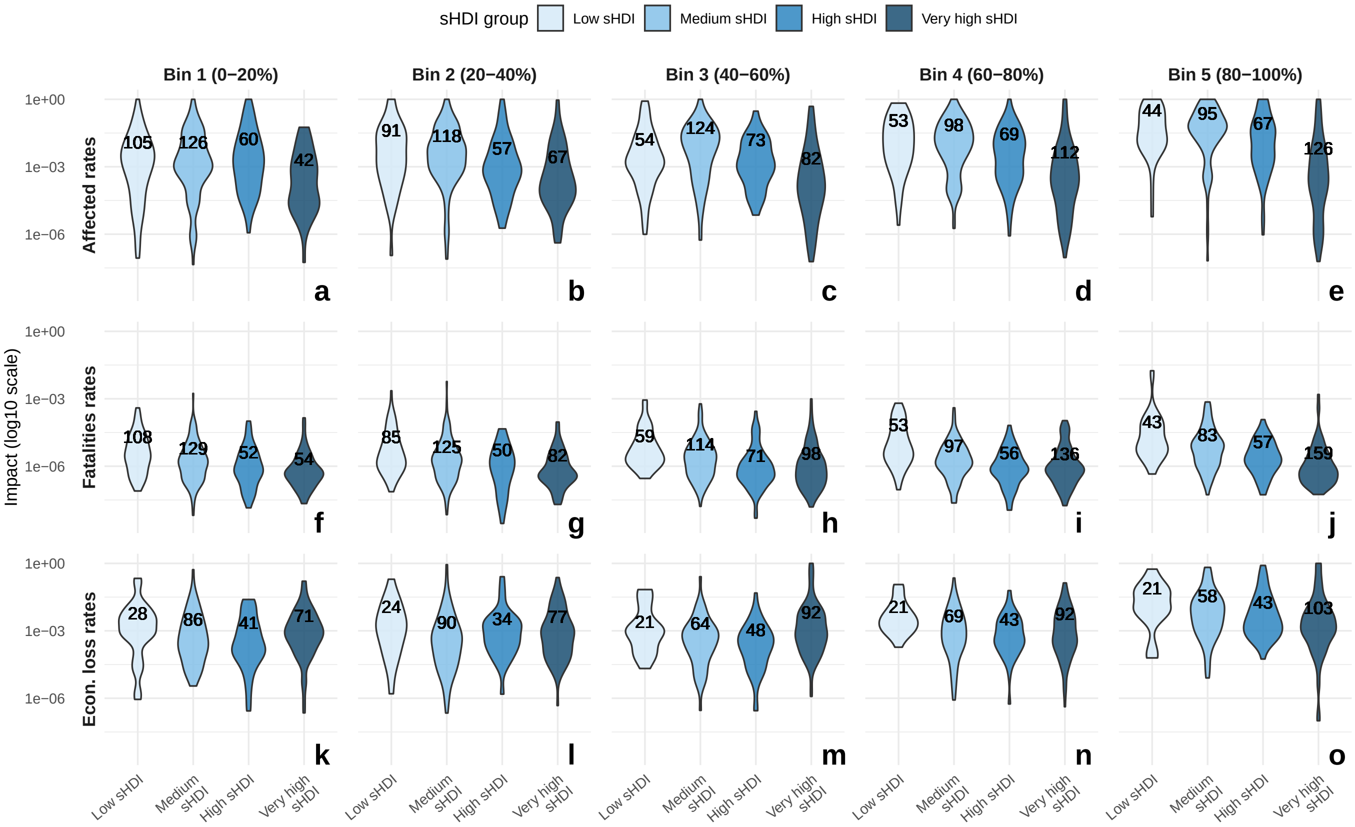

Supplementary Figure 22: Hazard intensity stratification of storm impacts per subnational Human Development Index (sHDI) group. Storm intensities are divided into quintiles (0–20%, 20–40%, 40–60%, 60–80%, 80–100%) based on maximum daily wind gust anomalies. Panels are labeled from left to right and top to bottom as (a–e) affected rates, (f–j) fatality rates, and (k–o) economic loss rates, with columns corresponding to increasing intensity bins. Violin plots depict the distributions of affected rates, fatality rates, and economic loss rates (log10 scale) across sHDI groups; event counts after filtering ($n \geq 20$) are shown above each distribution. The observed ordering of impacts across sHDI groups remains consistent across intensity quintiles, suggesting that variation in impacts is not solely explained by hazard intensity. Violin plots show the full distribution of the impact rates, with width proportional to the estimated probability density.

Supplementary Table 1: Counts and percentages of disaster types.

Disaster type	count	percentage (%)
Flood	3694	52.32
Storm	2090	29.60
Landslide	521	7.38
Cold wave	260	3.68
Heat wave	125	1.77
Wildfire	239	3.38
Drought	132	1.87

Disaster types follow the classification shown in Supplementary Table 23.

Supplementary Table 2: Counts of event types per subnational Human Development Index (sHDI) group from 1990 to 2020.

Disaster type	Low sHDI	Medium sHDI	High sHDI	Very high sHDI
Flood	1145	1228	744	577
Storm	404	634	373	679
Landslide	163	250	73	35
Cold wave	31	49	92	88
Heat wave	16	18	18	73
Wildfire	15	37	46	141
Drought	66	33	23	10

Event data are from the Emergency Events Database (EM-DAT). The total number of events is 7,061. Floods are the most frequent disaster type across all sHDI groups, while cold waves, heat waves, and wildfires are predominantly reported in very high sHDI regions.

Supplementary Table 3: Counts of impacted regions by subnational Human Development Index (sHDI) group and deviation from national Human Development Index (HDI) from 1990 to 2020.

sHDI group	better-off	nat-average	worse-off
Low sHDI	338	875	627
Medium sHDI	540	1162	547
High sHDI	333	888	148
Very high sHDI	202	1309	92

The deviation is defined as sHDI minus national HDI in the event year. Regions are classified, based on the distribution of deviations across all impacted regions, as worse-off ($\leq$20th percentile), national-average (between the 20th and 80th percentiles), or better-off ($\geq$80th percentile).

Supplementary Table 4: Count of the number of regions that have a different subnational Human Development Index (sHDI) compared to the national Human Development Index (HDI) per country and continent for the year 2020

Country	# Mismatch	HDI 2020	sHDI Categories	Continent
Nigeria	23	Low	Medium	Africa
Angola	12	Medium	High , Low	Africa
India	12	Medium	High	Asia/Pacific
Philippines	12	High	Medium	Asia/Pacific
Indonesia	11	High	Medium	Asia/Pacific
Tanzania	11	Low	High , Medium	Africa
Republic of the Congo	10	Medium	Low	Africa
China	9	High	Medium , Very high	Asia/Pacific
Ivory Coast	9	Medium	Low	Africa
Gabon	9	High	Medium	Africa
Mauritania	8	Medium	High , Low	Africa
Panama	8	Very high	High , Medium	America
Botswana	7	High	Medium	Africa
Georgia	7	Very high	High	Europe
Venezuela	7	Medium	High	America
Iran	6	High	Medium , Very high	Asia/Pacific
Serbia	6	Very high	High	Europe
Azerbaijan	5	High	Medium	Europe
Bulgaria	5	Very high	High	Europe
Bolivia	5	Medium	High	America
Bhutan	5	Medium	High , Low , Very high	Asia/Pacific
Colombia	5	High	Medium , Very high	America
Guyana	5	High	Medium	America
Malaysia	5	Very high	High	Asia/Pacific
Cameroon	4	Medium	Low	Africa
Egypt	4	High	Medium , Very high	Africa
Ethiopia	4	Low	High , Medium	Africa
Equatorial Guinea	4	Medium	High , Low	Africa
Mexico	4	High	Medium , Very high	America
Uruguay	4	Very high	High	America
Zambia	4	Medium	Low	Africa
Albania	3	High	Very high	Europe
Belarus	3	Very high	High	Europe
Chile	3	Very high	High	America
Jamaica	3	High	Medium	America
Cambodia	3	Medium	High , Low	Asia/Pacific
Laos	3	Medium	High , Low	Asia/Pacific
Madagascar	3	Low	Medium	Africa
Malawi	3	Low	Medium	Africa
Pakistan	3	Low	Medium	Asia/Pacific
Romania	3	Very high	High	Europe
Senegal	3	Low	Medium	Africa
Syria	3	Medium	Low	Asia/Pacific
Thailand	3	Very high	High	Asia/Pacific
Timor-Leste	3	Medium	High , Low	Asia/Pacific
Turkey	3	Very high	High	Asia/Pacific
Vietnam	3	High	Medium	Asia/Pacific
South Africa	3	High	Medium	Africa
Zimbabwe	3	Medium	High , Low	Africa
Benin	2	Low	Medium	Africa
Belize	2	Medium	High	America
Costa Rica	2	Very high	High	America

(Continued)

Country	# Mismatch	HDI 2020	sHDI Categories	Continent
France	2	Very high	High	Europe
Ghana	2	Medium	High , Low	Africa
Honduras	2	Medium	Low	America
Haiti	2	Low	Medium	America
Jordan	2	High	Medium	Asia/Pacific
Kazakhstan	2	Very high	High	Asia/Pacific
Kenya	2	Medium	Low	Africa
Mongolia	2	High	Medium	Asia/Pacific
Mauritius	2	Very high	High	Africa
Namibia	2	Medium	High , Low	Africa
Nicaragua	2	Medium	High , Low	America
Uganda	2	Low	Medium	Africa
Uzbekistan	2	High	Medium , Very high	Asia/Pacific
Vanuatu	2	Medium	Low	Asia/Pacific
Afghanistan	1	Low	Medium	Asia/Pacific
Burkina Faso	1	Low	Medium	Africa
Bangladesh	1	Medium	High	Asia/Pacific
Bosnia and Herzegovina	1	High	Very high	Europe
Brazil	1	High	Very high	America
Barbados	1	High	Very high	America
Central African Republic	1	Low	Medium	Africa
Democratic Republic of the Congo	1	Low	Medium	Africa
Comoros	1	Medium	Low	Africa
Cuba	1	High	Very high	America
Ecuador	1	High	Medium	America
Eritrea	1	Low	Medium	Africa
Guinea	1	Low	Medium	Africa
Gambia	1	Low	Medium	Africa
Guinea-Bissau	1	Low	Medium	Africa
Guatemala	1	Medium	High	America
Croatia	1	Very high	High	Europe
Kyrgyzstan	1	Medium	High	Asia/Pacific
Lebanon	1	High	Medium	Asia/Pacific
Libya	1	High	Medium	Africa
Lesotho	1	Low	Medium	Africa
Morocco	1	Medium	High	Africa
Moldova	1	High	Very high	Europe
Mali	1	Low	Medium	Africa
Myanmar (Burma)	1	Medium	Low	Asia/Pacific
Mozambique	1	Low	Medium	Africa
Peru	1	High	Very high	America
Rwanda	1	Low	Medium	Africa
Sierra Leone	1	Low	Medium	Africa
El Salvador	1	Medium	High	America
Suriname	1	High	Medium	America
Togo	1	Low	Medium	Africa
Tajikistan	1	Medium	High	Asia/Pacific
Trinidad and Tobago	1	Very high	High	America
Tunisia	1	High	Medium	Africa

The table shows countries where subnational regions fall into different human development categories than the country's overall national HDI for the year 2020. For example, Nigeria has a national Low HDI classification, but contains 23 regions classified as Medium HDI at the subnational level. The '# Mismatch' column indicates the number of subnational regions within each country that differ from the national HDI category. The four HDI/sHDI groups follow the United Nations Development Programme thresholds: low (< 0.55), medium ($0.55 \leq \text{sHDI} < 0.70$), high ($0.70 \leq \text{sHDI} < 0.80$).

Supplementary Table 5: Total count and proportion of exposure and impacts from 1990 to 2020 per subnational Human Development Index (sHDI) group.

sHDI category	Variable	Total count	Percentage (%)
Low sHDI	pop exposure	3.48e+10	28.71
Medium sHDI	pop exposure	4.37e+10	35.99
High sHDI	pop exposure	2.37e+10	19.51
Very high sHDI	pop exposure	1.92e+10	15.79
Low sHDI	Total affected	1.01e+09	41.21
Medium sHDI	Total affected	1.02e+09	41.89
High sHDI	Total affected	2.91e+08	11.91
Very high sHDI	Total affected	1.22e+08	4.99
Low sHDI	Total fatalities	4.31e+05	56.57
Medium sHDI	Total fatalities	1.43e+05	18.71
High sHDI	Total fatalities	2.61e+04	3.42
Very high sHDI	Total fatalities	1.62e+05	21.3
Low sHDI	GDP exposure	9.59e+13	6.58
Medium sHDI	GDP exposure	2.59e+14	17.8
High sHDI	GDP exposure	3.08e+14	21.14
Very high sHDI	GDP exposure	7.94e+14	54.48
Low sHDI	Economic loss	1.27e+11	6.51
Medium sHDI	Economic loss	2.93e+11	15.05
High sHDI	Economic loss	2.48e+11	12.76
Very high sHDI	Economic loss	1.28e+12	65.68

Population exposure ("pop exposure") is the sum of the exposed population across all events; economic exposure ("GDP exposure") is the sum of exposed gross domestic product (GDP) in constant 2011 USD. Total affected and total fatalities are summed across reported events; economic losses are in constant 2011 USD. Percentages are computed within each variable and sum to 100% across the four sHDI groups.

Supplementary Table 6: Results from quantile regression analysis to estimate trends in population and economic exposure and impacts (human and economic losses) from 1990 to 2020 for each subnational Human Development Index (sHDI) group.

impact	sHDI group	value 1990	value 2020	slope	*p*-value
pop exposure (billion people)	all	1.34	4.12	1.51e-01	3.14e-08
pop exposure (billion people)	Low sHDI	1.01	0.57	-2.97e-02	1.00e-01
pop exposure (billion people)	Medium sHDI	0.08	0.88	8.02e-02	2.27e-04
pop exposure (billion people)	High sHDI	0.04	1.60	6.54e-02	1.85e-05
pop exposure (billion people)	Very high sHDI	0.21	1.06	2.60e-02	1.60e-05
affected (million people)	all	62.23	78.53	7.38e-01	3.50e-01
affected (million people)	Low sHDI	55.17	20.20	-7.57e-01	1.39e-01
affected (million people)	Medium sHDI	6.71	37.05	1.03e+00	6.00e-02
affected (million people)	High sHDI	0.19	20.53	5.50e-01	6.35e-04
affected (million people)	Very high sHDI	0.15	0.75	5.03e-02	1.08e-02
fatalities (thousand people)	all	5.42	12.59	-7.44e-02	8.64e-01
fatalities (thousand people)	Low sHDI	3.57	1.86	-1.85e-01	4.60e-02
fatalities (thousand people)	Medium sHDI	1.51	2.29	6.79e-03	8.82e-01
fatalities (thousand people)	High sHDI	0.16	1.51	4.06e-02	3.97e-04
fatalities (thousand people)	Very high sHDI	0.18	6.94	4.89e-02	1.71e-01
GDP exposure (trillion US$)	all	12.83	69.67	2.41e+00	1.59e-09
GDP exposure (trillion US$)	Low sHDI	2.25	1.64	-7.95e-02	1.22e-01
GDP exposure (trillion US$)	Medium sHDI	0.69	4.67	4.63e-01	6.00e-04
GDP exposure (trillion US$)	High sHDI	0.90	20.09	7.85e-01	2.65e-05
GDP exposure (trillion US$)	Very high sHDI	8.99	43.28	1.11e+00	5.39e-05
Economic loss (billion US$)	all	24.89	66.61	9.76e-01	1.80e-01
Economic loss (billion US$)	Low sHDI	8.93	2.51	-1.74e-01	4.23e-02
Economic loss (billion US$)	Medium sHDI	3.55	19.20	7.87e-02	6.18e-01
Economic loss (billion US$)	High sHDI	1.58	12.77	4.61e-01	2.90e-04
Economic loss (billion US$)	Very high sHDI	10.82	32.12	9.59e-01	1.50e-01

Population exposure ("pop exposure") is the sum of the exposed population across all events; economic exposure ("GDP exposure") is the sum of exposed gross domestic product (GDP) in constant 2011 USD. Slopes are estimated using quantile regression (τ = 0.5, the 0.5 quantile). The values for 1990 and 2020 are the corresponding sum of each variable at the start and end years of the analysis.

Supplementary Table 7: Total counts and proportion of exposure and impacts from 1990 to 2020 per disaster type.

Variable	Disaster type	Total count	Prop (%)
Pop exposure	Flood	5.25e+10	43.23
Pop exposure	Storm	5.02e+10	41.4
Pop exposure	Landslide	2.81e+09	2.31
Pop exposure	Cold wave	7.15e+09	5.89
Pop exposure	Heat wave	5.31e+09	4.37
Pop exposure	Wildfire	1.55e+09	1.28
Pop exposure	Drought	1.83e+09	1.51
Total affected	Flood	1.33e+09	54.5
Total affected	Storm	8.01e+08	32.75
Total affected	Landslide	6.66e+06	0.27
Total affected	Cold wave	1.49e+07	0.61
Total affected	Heat wave	1.88e+06	0.08
Total affected	Wildfire	3.88e+06	0.16
Total affected	Drought	2.85e+08	11.63
Total fatalities	Flood	1.61e+05	21.16
Total fatalities	Storm	3.81e+05	50.03
Total fatalities	Landslide	2.63e+04	3.45
Total fatalities	Cold wave	1.37e+04	1.79
Total fatalities	Heat wave	1.55e+05	20.28
Total fatalities	Wildfire	2.17e+03	0.29
Total fatalities	Drought	2.29e+04	3.01
GDP exposure	Flood	4.43e+14	30.41
GDP exposure	Storm	7.47e+14	51.24
GDP exposure	Landslide	1.80e+13	1.24
GDP exposure	Cold wave	8.56e+13	5.88
GDP exposure	Heat wave	9.25e+13	6.35
GDP exposure	Wildfire	5.07e+13	3.48
GDP exposure	Drought	2.05e+13	1.4
Economic loss	Flood	5.60e+11	28.8
Economic loss	Storm	1.19e+12	61.22
Economic loss	Landslide	7.70e+09	0.4
Economic loss	Cold wave	1.01e+10	0.52
Economic loss	Heat wave	1.58e+10	0.81
Economic loss	Wildfire	1.03e+11	5.3
Economic loss	Drought	5.74e+10	2.95

Population exposure (”pop exposure”) is the sum of the exposed population across all events; economic exposure (”GDP exposure”) is the sum of exposed gross domestic product (GDP) in constant 2011 USD.

Supplementary Table 8: Results from quantile regression analysis to estimate trends in human (affected and fatality rates) and economic loss rates from 1990 to 2020 overall and for each subnational Human Development Index (sHDI) group.

Impact rate	sHDI group	rate 1990	rate 2020	% change	slope	p-value	npoints
Affected (‱people)	all	34.05	32.79	-3.69	-4.19e-02	8.54e-01	5753
Affected (‱people)	Low sHDI	63.18	95.10	50.51	1.06e+00	2.49e-01	1573
Affected (‱people)	Medium sHDI	67.39	37.56	-44.26	-9.94e-01	6.70e-02	1904
Affected (‱people)	High sHDI	7.27	38.29	426.58	1.03e+00	2.18e-03	1159
Affected (‱people)	Very high sHDI	4.56	5.14	12.72	1.93e-02	8.08e-01	1117
Fatalities (‱people)	all	0.06	0.02	-69.77	-1.28e-03	0.00e+00	5487
Fatalities (‱people)	Low sHDI	0.08	0.05	-40.09	-1.12e-03	3.30e-02	1466
Fatalities (‱people)	Medium sHDI	0.07	0.02	-76.45	-1.88e-03	3.49e-09	1860
Fatalities (‱people)	High sHDI	0.05	0.01	-80.10	-1.27e-03	2.43e-07	1004
Fatalities (‱people)	Very high sHDI	0.01	0.01	2.55	9.33e-06	9.40e-01	1157
Econ. loss (% GDP)	all	0.16	0.07	-58.01	-3.19e-03	1.10e-04	2580
Econ. loss (% GDP)	Low sHDI	0.15	0.29	98.00	4.76e-03	3.45e-01	363
Econ. loss (% GDP)	Medium sHDI	0.17	0.03	-80.76	-4.48e-03	3.86e-05	864
Econ. loss (% GDP)	High sHDI	0.19	0.03	-82.03	-5.29e-03	1.14e-02	516
Econ. loss (% GDP)	Very high sHDI	0.13	0.11	-17.77	-7.86e-04	7.09e-01	837

The affected and fatality rates are both estimated in parts per $10,000$ (‱) of exposed population, and the economic loss rates are calculated in percentage of exposed gross domestic product (GDP). The slopes are estimated using quantile regression ($\tau = 0.5$, the 0.5 quantile). The rates for 1990 and 2020 correspond to the median values at both years.

Supplementary Table 9: Results from quantile regression analysis to estimate trends in annual population exposure from 1990 to 2020 for each subnational Human Development Index (sHDI) group per disaster type.

Impact	sHDI group	disaster type	count 1990	count 2020	slope	p-value	npoints
pop exposure	all	Flood	505.56	2353.07	57.73	5.03e-08	31
pop exposure	all	Storm	605.06	1508.92	66.66	1.42e-05	31
pop exposure	all	Landslide	49.67	22.45	-1.69	3.51e-01	31
pop exposure	all	Cold wave	125.12	5.37	11.75	7.07e-02	31
pop exposure	all	Wildfire	13.35	21.20	0.72	4.66e-01	31
pop exposure	all	Heat wave	38.29	160.61	4.52	3.60e-01	30
pop exposure	all	Drought	7.37	45.11	2.24	1.90e-01	25
pop exposure	Low sHDI	Flood	447.73	464.61	-12.87	1.28e-01	31
pop exposure	Low sHDI	Storm	420.32	74.09	-11.46	1.04e-03	31
pop exposure	Low sHDI	Landslide	48.41	7.82	-1.72	2.14e-01	31
pop exposure	Low sHDI	Cold wave	92.48		-0.99	9.30e-01	17
pop exposure	Low sHDI	Wildfire			0.02	9.36e-01	14
pop exposure	Low sHDI	Heat wave			3.25	7.25e-01	14
pop exposure	Low sHDI	Drought		26.61	0.25	8.44e-01	20
pop exposure	Medium sHDI	Flood	31.89	602.13	38.96	8.62e-06	31
pop exposure	Medium sHDI	Storm	40.77	264.17	21.41	2.75e-02	31
pop exposure	Medium sHDI	Landslide	0.90	10.94	0.91	6.86e-02	31
pop exposure	Medium sHDI	Cold wave			0.94	4.41e-01	22
pop exposure	Medium sHDI	Wildfire	0.17	3.42	0.46	4.64e-01	19
pop exposure	Medium sHDI	Heat wave	1.41		2.03	3.28e-01	12
pop exposure	Medium sHDI	Drought	7.37		0.20	9.28e-01	15
pop exposure	High sHDI	Flood	24.04	1135.01	34.20	2.72e-06	31
pop exposure	High sHDI	Storm		422.19	30.30	7.69e-05	30
pop exposure	High sHDI	Landslide	0.36	3.24	0.10	5.11e-01	25
pop exposure	High sHDI	Cold wave		5.37	0.31	7.37e-01	27
pop exposure	High sHDI	Wildfire	13.19	17.15	0.29	5.77e-01	21
pop exposure	High sHDI	Heat wave			-0.04	9.29e-01	10
pop exposure	High sHDI	Drought		18.50	-0.47	6.91e-01	14
pop exposure	Very high sHDI	Flood	1.90	151.32	6.04	2.31e-05	31
pop exposure	Very high sHDI	Storm	143.96	748.48	15.01	5.60e-05	31
pop exposure	Very high sHDI	Landslide		0.45	-0.11	7.42e-01	17
pop exposure	Very high sHDI	Cold wave	32.64		0.81	7.83e-01	21
pop exposure	Very high sHDI	Wildfire		0.63	-0.49	5.43e-01	30
pop exposure	Very high sHDI	Heat wave	36.88	160.61	5.53	8.36e-03	24
pop exposure	Very high sHDI	Drought			-0.79	3.94e-01	8

Missing count values indicate years with insufficient data. All slopes are calculated using the full time series of annual data (see npoints column), not just the 1990 and 2020 values shown. Population exposure (”pop exposure”) is the sum of the exposed population across all events. The slopes represent the change in exposed population (millions) per year and are estimated using quantile regression ($\tau = 0.5$, the 0.5 quantile). Values for 1990 and 2020 represent the annual sum of exposed population per sHDI group and disaster type for those years. npoints: number of data points used in the regression analysis.

Supplementary Table 10: Results from quantile regression analysis to estimate trends in annual reported affected people from 1990 to 2020 for each subnational Human Development Index (sHDI) group per disaster type.

Impact	sHDI group	disaster type	count 1990	count 2020	slope	p-value	npoints
affected	all	Flood	44.99	28.59	-0.38	5.37e-01	31
affected	all	Storm	16.97	40.24	0.39	2.57e-01	31
affected	all	Landslide	0.01	0.18	-0.00	7.53e-01	31
affected	all	Drought	0.25	9.22	0.25	2.26e-01	23
affected	all	Wildfire		0.27	0.00	5.20e-01	30
affected	all	Heat wave			0.00	8.56e-01	18
affected	all	Cold wave		0.03	0.02	3.64e-01	26
affected	Low sHDI	Flood	44.67	8.89	-0.71	1.32e-01	31
affected	Low sHDI	Storm	10.50	2.09	-0.39	1.51e-02	31
affected	Low sHDI	Landslide	0.01	0.00	0.00	9.32e-01	29
affected	Low sHDI	Drought		9.22	0.48	4.19e-02	19
affected	Low sHDI	Wildfire			0.00	9.10e-01	13
affected	Low sHDI	Heat wave			0.00	9.10e-01	13
affected	Low sHDI	Cold wave			0.00	6.06e-01	10
affected	Medium sHDI	Flood	0.13	6.86	0.73	1.27e-02	31
affected	Medium sHDI	Storm	6.32	29.89	0.14	7.15e-01	31
affected	Medium sHDI	Landslide		0.16	0.00	9.05e-01	30
affected	Medium sHDI	Drought	0.25		-0.05	9.54e-01	14
affected	Medium sHDI	Wildfire		0.14	0.00	4.39e-01	14
affected	Medium sHDI	Heat wave			0.00	4.39e-01	14
affected	Medium sHDI	Cold wave			0.00	9.54e-01	16
affected	High sHDI	Flood	0.19	12.54	0.25	2.46e-03	31
affected	High sHDI	Storm		7.81	0.32	1.76e-03	29
affected	High sHDI	Landslide		0.02	0.00	5.79e-01	20
affected	High sHDI	Drought			0.00	5.79e-01	20
affected	High sHDI	Wildfire		0.13	0.00	9.35e-01	16
affected	High sHDI	Heat wave			0.00	9.35e-01	16
affected	High sHDI	Cold wave		0.03	0.01	6.28e-01	22
affected	Very high sHDI	Flood	0.00	0.30	0.01	2.37e-01	31
affected	Very high sHDI	Storm	0.15	0.45	0.01	3.32e-01	31
affected	Very high sHDI	Landslide		0.00	0.00	7.21e-01	17
affected	Very high sHDI	Drought			0.00	7.21e-01	17
affected	Very high sHDI	Wildfire		0.00	0.00	7.29e-01	28
affected	Very high sHDI	Heat wave			0.00	9.19e-01	14
affected	Very high sHDI	Cold wave			0.00	5.40e-01	10

Missing count values indicate years with insufficient data. All slopes are calculated using the full time series of annual data (see npoints column), not just the 1990 and 2020 values shown. Affected population (”affected”) is the sum of the reported affected people by the Emergency Events Database (EM-DAT) across all events in the considered sHDI group and disaster type. The slopes represent the change in affected population (millions) per year and are estimated using quantile regression ($\tau = 0.5$, the 0.5 quantile). Values for 1990 and 2020 represent the annual sum of affected population per sHDI group and disaster type for those years. npoints: number of data points used in the regression analysis.

Supplementary Table 11: Results from quantile regression analysis to estimate trends in annual reported fatalities from 1990 to 2020 for each subnational Human Development Index (sHDI) group per disaster type.

Impact	sHDI group	disaster type	count 1990	count 2020	slope	p-value	npoints
fatalities	all	Flood	1.88	3.91	-0.06	2.79e-01	31
fatalities	all	Landslide	0.17	0.51	-0.01	2.68e-01	31
fatalities	all	Storm	2.80	1.55	-0.06	3.40e-01	31
fatalities	all	Cold wave	0.18	0.00	-0.00	8.47e-01	31
fatalities	all	Heat wave	0.39	6.57	0.01	7.97e-01	30
fatalities	all	Wildfire		0.04	0.00	1.00e+00	30
fatalities	all	Drought			-0.04	8.97e-01	8
fatalities	Low sHDI	Flood	1.41	1.61	-0.07	4.32e-02	31
fatalities	Low sHDI	Landslide	0.10	0.04	-0.01	4.06e-02	31
fatalities	Low sHDI	Storm	1.88	0.20	-0.06	1.60e-01	31
fatalities	Low sHDI	Cold wave	0.18		-0.01	6.02e-01	17
fatalities	Low sHDI	Heat wave			0.00	9.58e-01	14
fatalities	Low sHDI	Wildfire			-0.00	2.11e-01	11
fatalities	Low sHDI	Drought			-0.08	9.04e-02	6
fatalities	Medium sHDI	Flood	0.27	1.49	0.02	3.14e-01	31
fatalities	Medium sHDI	Landslide	0.05	0.23	-0.00	9.34e-01	31
fatalities	Medium sHDI	Storm	0.81	0.57	-0.02	4.54e-01	31
fatalities	Medium sHDI	Cold wave			0.00	3.86e-01	18
fatalities	Medium sHDI	Heat wave	0.38		0.00	9.31e-01	12
fatalities	Medium sHDI	Wildfire		0.00	-0.00	4.23e-01	15
fatalities	Medium sHDI	Drought			-0.04	9.86e-01	3
fatalities	High sHDI	Flood	0.15	0.61	0.02	8.10e-06	31
fatalities	High sHDI	Landslide	0.01	0.22	0.00	5.39e-01	23
fatalities	High sHDI	Storm		0.64	0.01	1.88e-02	30
fatalities	High sHDI	Cold wave		0.00	-0.01	6.00e-02	26
fatalities	High sHDI	Heat wave			-0.00	9.38e-01	9
fatalities	High sHDI	Wildfire		0.04	0.00	8.68e-01	17
fatalities	Very high sHDI	Flood	0.06	0.19	0.01	1.32e-02	30
fatalities	Very high sHDI	Landslide		0.03	-0.00	3.88e-01	15
fatalities	Very high sHDI	Storm	0.11	0.15	0.00	4.67e-01	31
fatalities	Very high sHDI	Cold wave			0.00	8.32e-01	19
fatalities	Very high sHDI	Heat wave	0.01	6.57	0.01	8.67e-01	23
fatalities	Very high sHDI	Wildfire			0.00	6.59e-01	26

Missing count values indicate years with insufficient data. All slopes are calculated using the full time series of annual data (see npoints column), not just the 1990 and 2020 values shown. "fatalities" is the sum of the reported fatalities by the Emergency Events Database (EM-DAT) across all events in the considered sHDI group and disaster type. The slopes represent the change in fatalities (thousands) per year and are estimated using quantile regression (τ = 0.5, the 0.5 quantile). Values for 1990 and 2020 represent the annual sum of fatalities per sHDI group and disaster type for those years. npoints: number of data points used in the regression analysis.

Supplementary Table 12: Results from quantile regression analysis to estimate trends in economic exposure from 1990 to 2020 for each subnational Human Development Index (sHDI) group per disaster type.

Impact	sHDI group	disaster type	count 1990	count 2020	slope	p-value	npoints
GDP exposure	all	Flood	1.64	25.54	0.80	4.93e-13	31
GDP exposure	all	Storm	7.18	36.51	1.07	2.96e-06	31
GDP exposure	all	Landslide	0.08	0.16	-0.01	4.25e-01	31
GDP exposure	all	Cold wave	1.62	0.08	0.07	1.78e-01	31
GDP exposure	all	Wildfire	0.52	0.21	0.02	6.26e-01	31
GDP exposure	all	Heat wave	1.76	6.71	0.19	1.51e-02	30
GDP exposure	all	Drought	0.03	0.47	0.02	1.75e-01	25
GDP exposure	Low sHDI	Flood	0.95	1.31	-0.02	4.52e-01	31
GDP exposure	Low sHDI	Storm	0.97	0.22	-0.02	5.89e-02	31
GDP exposure	Low sHDI	Landslide	0.07	0.01	-0.01	1.22e-01	31
GDP exposure	Low sHDI	Cold wave	0.26		0.00	8.85e-01	17
GDP exposure	Low sHDI	Wildfire			0.00	9.24e-01	14
GDP exposure	Low sHDI	Heat wave			0.03	4.49e-01	14
GDP exposure	Low sHDI	Drought		0.10	0.00	1.39e-01	20
GDP exposure	Medium sHDI	Flood	0.28	2.89	0.26	9.53e-09	31
GDP exposure	Medium sHDI	Storm	0.36	1.71	0.14	1.52e-02	31
GDP exposure	Medium sHDI	Landslide	0.00	0.05	0.00	5.46e-01	31
GDP exposure	Medium sHDI	Cold wave			0.00	9.04e-01	22
GDP exposure	Medium sHDI	Wildfire	0.00	0.01	-0.00	9.82e-01	19
GDP exposure	Medium sHDI	Heat wave	0.03		0.01	5.62e-01	12
GDP exposure	Medium sHDI	Drought	0.03		0.00	9.00e-01	15
GDP exposure	High sHDI	Flood	0.36	15.14	0.40	2.05e-05	31
GDP exposure	High sHDI	Storm		4.27	0.32	1.73e-04	30
GDP exposure	High sHDI	Landslide	0.01	0.05	0.00	1.50e-01	25
GDP exposure	High sHDI	Cold wave		0.08	-0.00	9.37e-01	27
GDP exposure	High sHDI	Wildfire	0.52	0.17	0.00	9.16e-01	21
GDP exposure	High sHDI	Heat wave			-0.01	2.40e-01	10
GDP exposure	High sHDI	Drought		0.37	0.00	9.31e-01	14
GDP exposure	Very high sHDI	Flood	0.05	6.20	0.22	6.00e-04	31
GDP exposure	Very high sHDI	Storm	5.85	30.30	0.66	1.75e-04	31
GDP exposure	Very high sHDI	Landslide		0.04	-0.00	8.14e-01	17
GDP exposure	Very high sHDI	Cold wave	1.36		0.02	8.29e-01	21
GDP exposure	Very high sHDI	Wildfire		0.02	-0.03	4.15e-01	30
GDP exposure	Very high sHDI	Heat wave	1.73	6.71	0.19	1.34e-02	24
GDP exposure	Very high sHDI	Drought			-0.06	4.11e-01	8

Missing count values indicate years with insufficient data. All slopes are calculated using the full time series of annual data (see npoints column), not just the 1990 and 2020 values shown. "GDP exposure" is the sum of the exposed gross domestic product (GDP) across all events in the considered sHDI group and disaster type. The slopes represent the change in exposed GDP (trillion in constant 2011 US$) per year and are estimated using quantile regression ($\tau = 0.5$, the 0.5 quantile). Values for 1990 and 2020 represent the annual sum of exposed GDP per sHDI group and disaster type for those years. npoints: number of data points used in the regression analysis.

Supplementary Table 13: Results from quantile regression analysis to estimate trends in economic loss rates from 1990 to 2020 for each subnational Human Development Index (sHDI) group per disaster type.

Impact	sHDI group	disaster type	count 1990	count 2020	slope	p-value	npoints
Econ. loss	all	Flood	5.38	22.93	0.38	2.17e-01	31
Econ. loss	all	Storm	17.63	40.59	1.07	2.25e-02	31
Econ. loss	all	Cold wave	0.07		0.02	6.50e-01	15
Econ. loss	all	Wildfire	1.70	0.15	0.01	9.08e-01	31
Econ. loss	all	Drought	0.11	2.83	0.08	1.22e-01	22
Econ. loss	all	Landslide		0.11	0.00	5.57e-01	26
Econ. loss	Low sHDI	Flood	1.82	1.65	-0.06	4.88e-01	29
Econ. loss	Low sHDI	Storm	7.11	0.64	-0.06	2.67e-01	27
Econ. loss	Low sHDI	Landslide			0.00	7.15e-01	11
Econ. loss	Medium sHDI	Flood	0.56	4.16	0.07	4.88e-01	31
Econ. loss	Medium sHDI	Storm	2.69	15.04	-0.03	7.56e-01	31
Econ. loss	Medium sHDI	Landslide			-0.00	6.86e-01	17
Econ. loss	High sHDI	Flood	0.07	7.08	0.14	6.62e-03	31
Econ. loss	High sHDI	Storm		2.94	0.28	1.27e-03	29
Econ. loss	High sHDI	Wildfire	1.51	0.14	-0.01	7.19e-01	12
Econ. loss	High sHDI	Drought		2.61	0.03	7.64e-01	12
Econ. loss	Very high sHDI	Flood	2.93	10.03	0.20	2.24e-01	31
Econ. loss	Very high sHDI	Storm	7.83	21.97	0.51	2.66e-01	31
Econ. loss	Very high sHDI	Wildfire		0.01	0.07	2.79e-01	29

Missing count values indicate years with insufficient data. All slopes are calculated using the full time series of annual data (see npoints column), not just the 1990 and 2020 values shown. Economic loss "Econ. loss" is the sum of the reported economic losses by the Emergency Events Database (EM-DAT) across all events in the considered sHDI group and disaster type. The slopes represent the change in economic losses (billion in constant 2011 US$) per year and are estimated using quantile regression ($\tau = 0.5$, the 0.5 quantile). Values for 1990 and 2020 represent the annual sum of economic losses per sHDI group and disaster type for those years. npoints: number of data points used in the regression analysis.

Supplementary Table 14: Results from quantile regression analysis to estimate trends in fatality rates (‱ people) from 1990 to 2020 per subnational Human Development Index (sHDI) group and disaster type.

Impact	sHDI group	disaster type	rate 1990 (‱)	rate 2020 (‱)	(%) change	slope	*p*-value	npoints
fatalities	all	Flood	0.07	0.02	-75.17	-1.71e-03	0.00e+00	2783
fatalities	all	Storm	0.03	0.01	-78.65	-7.29e-04	8.88e-16	1711
fatalities	all	Landslide	0.37	0.25	-32.52	-4.06e-03	2.98e-01	502
fatalities	all	Cold wave	0.06	0.00	-96.73	-2.07e-03	1.48e-02	221
fatalities	all	Heat wave	0.01	0.09	557.42	2.64e-03	1.89e-01	117
fatalities	all	Wildfire	0.02	0.03	44.26	3.41e-04	7.16e-01	144
fatalities	Low sHDI	Flood	0.10	0.04	-61.24	-2.04e-03	3.19e-03	893
fatalities	Low sHDI	Storm	0.04	0.05	24.67	3.01e-04	6.55e-01	348
fatalities	Low sHDI	Landslide	0.34	0.38	13.38	1.50e-03	8.50e-01	162
fatalities	Low sHDI	Cold wave	0.04	0.00	-90.54	-1.25e-03	8.06e-01	30
fatalities	Low sHDI	Heat wave	0.01	0.00	-79.09	-3.25e-04	9.65e-01	16
fatalities	Low sHDI	Wildfire	0.10	0.06	-40.67	-1.38e-03	9.67e-01	11
fatalities	Medium sHDI	Flood	0.08	0.02	-78.65	-2.10e-03	1.60e-09	992
fatalities	Medium sHDI	Storm	0.03	0.01	-65.37	-6.29e-04	2.81e-03	548
fatalities	Medium sHDI	Landslide	0.44	0.07	-83.78	-1.23e-02	5.51e-03	240
fatalities	Medium sHDI	Cold wave	0.04	0.01	-87.18	-1.25e-03	5.77e-01	35
fatalities	Medium sHDI	Heat wave	0.08	0.13	49.38	1.39e-03	9.65e-01	16
fatalities	Medium sHDI	Wildfire	0.03	0.01	-71.32	-7.28e-04	6.55e-01	26
fatalities	High sHDI	Flood	0.05	0.01	-75.93	-1.16e-03	3.65e-04	531
fatalities	High sHDI	Storm	0.03	0.00	-87.49	-9.38e-04	3.47e-05	286
fatalities	High sHDI	Landslide	1.00	0.42	-58.09	-1.94e-02	6.01e-01	69
fatalities	High sHDI	Cold wave	0.09	0.01	-83.61	-2.39e-03	2.68e-01	73
fatalities	High sHDI	Heat wave	0.01	0.09	557.42	2.64e-03	9.40e-01	14
fatalities	High sHDI	Wildfire	0.08	0.01	-92.41	-2.45e-03	5.44e-01	31
fatalities	Very high sHDI	Flood	0.01	0.02	21.16	9.35e-05	7.04e-01	367
fatalities	Very high sHDI	Storm	0.01	0.01	-38.11	-1.04e-04	1.62e-01	529
fatalities	Very high sHDI	Landslide	0.31	0.38	23.25	2.40e-03	9.48e-01	31
fatalities	Very high sHDI	Cold wave	0.03	0.01	-70.65	-6.59e-04	7.15e-01	83
fatalities	Very high sHDI	Heat wave	0.07	0.07	5.50	1.28e-04	9.83e-01	71
fatalities	Very high sHDI	Wildfire		0.05		2.08e-03	2.58e-01	76

Missing rate values indicate cases where the regression fit produced negative values, which are not physically meaningful for fatality rates. Fatality rates "fatalities" are estimated in parts per $10,000$ (‱) of exposed people across all events in the considered sHDI group and disaster type. The slopes represent the change in median fatality rates per year and are estimated using quantile regression ($\tau = 0.5$, the 0.5 quantile). All slopes are calculated using the full impact rates (see npoints column), not just the shown rates for 1990 and 2020. The (%) change column represents the percent change between the 1990 and 2020 fitted rates. Values for 1990 and 2020 represent the regression fit per sHDI group and disaster type. npoints: number of data points used in the regression analysis.

Supplementary Table 15: Results from quantile regression analysis to estimate trends in affected rates (‱people) from 1990 to 2020 per subnational Human Development Index (sHDI) group and disaster type.

Impact	sHDI group	disaster type	rate 1990 (‱)	rate 2020 (‱)	(%) change	slope	p-value	npoints
affected	all	Flood	69.57	31.37	-54.91	-1.27e+00	1.35e-03	3307
affected	all	Storm	23.36	20.45	-12.48	-9.72e-02	7.34e-01	1663
affected	all	Landslide	7.50	6.44	-14.12	-3.53e-02	7.60e-01	335
affected	all	Cold wave		60.32		2.62e+00	1.16e-02	105
affected	all	Drought	1306.27	3563.52	172.80	7.52e+01	3.94e-01	106
affected	all	Heat wave		1.48		5.72e-02	1.63e-01	40
affected	all	Wildfire		18.87		6.88e-01	4.04e-03	196
affected	Low sHDI	Flood	157.19	60.44	-61.55	-3.22e+00	1.06e-02	1021
affected	Low sHDI	Storm	14.37	77.73	440.90	2.11e+00	3.56e-02	347
affected	Low sHDI	Landslide	2.60	21.99	746.29	6.46e-01	4.57e-01	106
affected	Low sHDI	Cold wave	23.78	2.53	-89.34	-7.08e-01	8.81e-01	14
affected	Low sHDI	Drought	847.29	4663.22	450.37	1.27e+02	1.49e-01	65
affected	Low sHDI	Wildfire	50.16			-1.71e+00	6.25e-01	14
affected	Medium sHDI	Flood	87.96	32.30	-63.28	-1.86e+00	7.84e-03	1110
affected	Medium sHDI	Storm	86.96	62.52	-28.10	-8.14e-01	6.31e-01	561
affected	Medium sHDI	Landslide	10.58	1.27	-88.01	-3.10e-01	8.00e-02	152
affected	Medium sHDI	Cold wave	42.12	41.08	-2.48	-3.48e-02	9.99e-01	23
affected	Medium sHDI	Drought	1950.21	2275.64	16.69	1.08e+01	9.44e-01	28
affected	Medium sHDI	Wildfire		108.61		4.43e+00	3.38e-01	24
affected	High sHDI	Flood	26.82	35.83	33.62	3.00e-01	5.74e-01	679
affected	High sHDI	Storm	8.27	27.38	231.10	6.37e-01	1.93e-01	326
affected	High sHDI	Landslide	7.98	10.70	34.10	9.07e-02	9.15e-01	47
affected	High sHDI	Cold wave		110.87		5.26e+00	1.01e-03	54
affected	High sHDI	Drought	2664.07			-9.17e+01	5.49e-01	13
affected	High sHDI	Wildfire	1.17	10.18	768.00	3.00e-01	8.55e-01	31
affected	Very high sHDI	Flood	8.94	7.16	-19.93	-5.94e-02	7.35e-01	497
affected	Very high sHDI	Storm	3.88	1.55	-59.96	-7.76e-02	1.68e-01	429
affected	Very high sHDI	Landslide		10.51		3.82e-01	8.02e-01	30
affected	Very high sHDI	Cold wave		63.41		4.22e+00	9.80e-01	14
affected	Very high sHDI	Heat wave	3.65	0.88	-75.82	-9.21e-02	9.99e-01	19
affected	Very high sHDI	Wildfire		20.50		8.74e-01	3.31e-04	127

Missing rate values indicate cases where the regression fit produced negative values, which are not physically meaningful for fatality rates. Affected rates "affected" are estimated in parts per $10,000$ (‱) of exposed people across all events in the considered sHDI group and disaster type. The slopes represent the change in median affected rates per year and are estimated using quantile regression ($\tau = 0.5$, the 0.5 quantile). All slopes are calculated using the full impact rates (see npoints column), not just the shown rates for 1990 and 2020. The (%) change column represents the percent change between the 1990 and 2020 fitted rates. Values for 1990 and 2020 represent the regression fit per sHDI group and disaster type. npoints: number of data points used in the regression analysis.

Supplementary Table 16: Results from quantile regression analysis to estimate trends in economic loss rates (% GDP) from 1990 to 2020 per subnational Human Development Index (sHDI) group and disaster type.

Impact	sHDI group	disaster type	rate 1990	rate 2020	(%) change	slope	p-value	npoints
Econ. loss	all	Flood	0.19	0.07	-61.26	-3.81e-03	4.15e-03	1186
Econ. loss	all	Storm	0.14	0.05	-66.49	-3.13e-03	8.01e-04	1126
Econ. loss	all	Landslide	0.57			-1.93e-02	1.51e-01	65
Econ. loss	all	Wildfire		0.57		2.00e-02	2.08e-01	112
Econ. loss	all	Drought	0.43	0.63	46.77	6.70e-03	7.82e-01	43
Econ. loss	all	Heat wave	0.42			-1.86e-02	8.97e-01	10
Econ. loss	Low sHDI	Flood	0.15	0.15	0.39	1.98e-05	9.97e-01	219
Econ. loss	Low sHDI	Storm	0.11	0.51	376.43	1.35e-02	2.73e-01	115
Econ. loss	Low sHDI	Landslide	0.31	0.79	151.88	1.59e-02	7.55e-01	13
Econ. loss	Low sHDI	Drought		1.06		4.14e-02	1.91e-01	11
Econ. loss	Medium sHDI	Flood	0.19	0.06	-70.33	-4.51e-03	4.36e-02	434
Econ. loss	Medium sHDI	Storm	0.11	0.01	-91.70	-3.38e-03	9.15e-03	367
Econ. loss	Medium sHDI	Landslide	1.32			-5.08e-02	8.44e-01	28
Econ. loss	Medium sHDI	Wildfire	0.11	0.19	76.81	2.72e-03	9.97e-01	12
Econ. loss	Medium sHDI	Drought	1.54	0.35	-77.43	-3.97e-02	8.74e-01	16
Econ. loss	High sHDI	Flood	0.21	0.04	-82.38	-5.70e-03	3.27e-02	254
Econ. loss	High sHDI	Storm	0.15	0.03	-76.24	-3.69e-03	8.07e-02	209
Econ. loss	High sHDI	Landslide	0.40	0.07	-82.84	-1.10e-02	9.00e-01	12
Econ. loss	High sHDI	Wildfire	0.29	1.62	457.30	4.43e-02	6.08e-01	15
Econ. loss	High sHDI	Drought	0.06	0.27	368.58	6.98e-03	8.07e-01	16
Econ. loss	Very high sHDI	Flood	0.17	0.16	-4.20	-2.34e-04	9.64e-01	279
Econ. loss	Very high sHDI	Storm	0.15	0.06	-59.61	-3.00e-03	1.20e-01	435
Econ. loss	Very high sHDI	Landslide	0.97	0.06	-93.76	-3.02e-02	8.23e-01	12
Econ. loss	Very high sHDI	Wildfire		0.52		2.01e-02	3.11e-01	85
Econ. loss	Very high sHDI	Heat wave	0.42			-1.86e-02	8.95e-01	10

Missing rate values indicate cases where the regression fit produced negative values, which are not physically meaningful for fatality rates. Economic loss rates "Econ. loss" are estimated in percent (%) of exposed gross domestic product (GDP; in constant 2011 US$) across all events in the considered sHDI group and disaster type. The slopes represent the change in median economic loss rates per year and are estimated using quantile regression ($\tau = 0.5$, the 0.5 quantile). All slopes are calculated using the full impact rates (see npoints column), not just the shown rates for 1990 and 2020. Values for 1990 and 2020 represent the regression fit per sHDI group and disaster type. npoints: number of data points used in the regression analysis.

Supplementary Table 17: Results from logistic regression analysis to estimate odds ratios of the likelihood of being affected per subnational Human Development Index (sHDI) group and disaster type.

Impact	disaster type	Case: sHDI group	Odds ratio	CI low	CI high	p-value	npoints
Likelihood affected	All types	Low : Very high	3.43	2.53	4.84	2.75e-14	2690
Likelihood affected	All types	Medium : Very high	2.43	1.80	3.42	3.69e-08	3021
Likelihood affected	All types	High : Very high	1.59	1.13	2.29	1.03e-02	2276
Likelihood affected	Flood	Low : Very high	4.70	3.09	7.55	1.80e-11	1518
Likelihood affected	Flood	Medium : Very high	3.77	2.42	6.10	1.47e-08	1607
Likelihood affected	Flood	High : Very high	2.25	1.40	3.80	1.44e-03	1176
Likelihood affected	Storm	Low : Very high	2.51	1.52	4.60	1.17e-03	776
Likelihood affected	Storm	Medium : Very high	2.28	1.43	4.06	1.67e-03	990
Likelihood affected	Storm	High : Very high	1.70	0.96	3.16	8.28e-02	755
Likelihood affected	Landslide	Low : Very high	1.72	0.43	8.94	4.77e-01	136
Likelihood affected	Landslide	Medium : Very high	0.57	0.18	2.87	4.21e-01	182
Likelihood affected	Landslide	High : Very high	0.98	0.10	5.88	9.85e-01	77
Likelihood affected	Cold wave	Low : Very high	0.15	0.01	11.17	3.42e-01	28
Likelihood affected	Cold wave	Medium : Very high	2.10	0.35	145.03	6.99e-01	37
Likelihood affected	Cold wave	High : Very high	0.69	0.11	48.18	8.46e-01	68
Likelihood affected	Wildfire	Low : Very high	0.43	0.09	2.02	2.84e-01	141
Likelihood affected	Wildfire	Medium : Very high	0.66	0.14	2.88	5.76e-01	151
Likelihood affected	Wildfire	High : Very high	1.98	0.15	11.56	5.35e-01	158
Likelihood affected	Heat wave	Low : Very high	0.00	0.00	0.01	2.14e-04	25
Likelihood affected	Heat wave	Medium : Very high	0.04	0.00	0.39	2.41e-01	25
Likelihood affected	Heat wave	High : Very high	0.00	0.00	0.03	1.26e-04	28
Likelihood affected	Drought	Low : Very high	3.97	2.85	5.48	2.15e-16	66
Likelihood affected	Drought	Medium : Very high	2.16	1.16	3.74	9.47e-03	29
Likelihood affected	Drought	High : Very high	0.51	0.21	0.89	6.36e-02	14

Odds ratios (OR) represent the likelihood of affected people in each sHDI group relative to Very high sHDI regions (reference group). ORs are calculated using affected rates (number of affected people per 10,000 exposed population). Impact data are from the Emergency Events Database (EM-DAT) for climate-related disasters occurring between 1990 and 2020. The four sHDI groups follow the United Nations Development Programme thresholds: low (sHDI < 0.55), medium ($0.55 \leq$ sHDI < 0.70), high ($0.70 \leq$ sHDI < 0.80), and very high (sHDI ≥ 0.80). CI: 95% confidence interval estimated by bootstrapping the logistic regression coefficients (5,000 iterations). npoints: number of observations used in the logistic regression model.

Supplementary Table 18: Results from logistic regression analysis to estimate odds ratios of the likelihood of fatalities per subnational Human Development Index (sHDI) group and disaster type.

Impact	disaster type	Case: sHDI group	Odds ratio	ci low	ci high	*p*-value	npoints
Likelihood fatalities	All types	Low : Very high	1.99	0.91	4.79	1.12e-01	2623
Likelihood fatalities	All types	Medium : Very high	1.58	0.72	3.76	2.93e-01	3017
Likelihood fatalities	All types	High : Very high	1.27	0.56	3.16	5.97e-01	2161
Likelihood fatalities	Flood	Low : Very high	2.99	1.64	5.63	5.91e-04	1260
Likelihood fatalities	Flood	Medium : Very high	3.04	1.46	6.63	4.15e-03	1359
Likelihood fatalities	Flood	High : Very high	2.39	1.04	5.06	3.26e-02	898
Likelihood fatalities	Storm	Low : Very high	8.24	2.16	23.06	4.01e-04	877
Likelihood fatalities	Storm	Medium : Very high	2.08	0.81	5.31	1.30e-01	1077
Likelihood fatalities	Storm	High : Very high	0.45	0.23	0.98	2.89e-02	815
Likelihood fatalities	Landslide	Low : Very high	0.38	0.12	3.38	2.85e-01	193
Likelihood fatalities	Landslide	Medium : Very high	0.26	0.09	2.25	1.23e-01	271
Likelihood fatalities	Landslide	High : Very high	0.60	0.18	5.23	5.69e-01	100
Likelihood fatalities	Cold wave	Low : Very high	3.00	1.03	7.44	2.86e-02	113
Likelihood fatalities	Cold wave	Medium : Very high	12.55	0.50	48.77	8.71e-02	118
Likelihood fatalities	Cold wave	High : Very high	2.02	1.00	4.37	6.31e-02	156
Likelihood fatalities	Wildfire	Low : Very high	0.76	0.15	3.49	7.27e-01	87
Likelihood fatalities	Wildfire	Medium : Very high	0.21	0.05	0.96	3.92e-02	102
Likelihood fatalities	Wildfire	High : Very high	0.36	0.11	1.51	1.23e-01	107
Likelihood fatalities	Heat wave	Low : Very high	0.11	0.01	0.36	1.32e-02	87
Likelihood fatalities	Heat wave	Medium : Very high	0.53	0.08	1.73	4.11e-01	87
Likelihood fatalities	Heat wave	High : Very high	0.13	0.02	0.47	1.50e-02	85

Odds ratios (OR) represent the likelihood of fatalities in each sHDI group relative to Very high sHDI regions (reference group). ORs are calculated using fatality rates (number of fatalities per 10,000 exposed population). Impact data are from the Emergency Events Database (EM-DAT) for climate-related disasters occurring between 1990 and 2020. The four sHDI groups follow the United Nations Development Programme thresholds: low (sHDI < 0.55), medium ($0.55 \leq$ sHDI < 0.70), high ($0.70 \leq$ sHDI < 0.80), and very high (sHDI ≥ 0.80). CI: 95% confidence interval estimated by bootstrapping the logistic regression coefficients (5,000 iterations). npoints: number of observations used in the logistic regression model.

Supplementary Table 19: Results from logistic regression analysis to estimate odds ratios of the likelihood of economic loss per subnational Human Development Index (sHDI) group and disaster type.

Impact	Disaster type	Case: sHDI group	Odds ratio	ci low	ci high	*p*-value	npoints
Likelihood Econ. loss	All types	Low : Very high	0.66	0.39	1.12	1.11e-01	1200
Likelihood Econ. loss	All types	Medium : Very high	0.65	0.41	1.02	6.30e-02	1701
Likelihood Econ. loss	All types	High : Very high	0.46	0.28	0.75	1.65e-03	1353
Likelihood Econ. loss	Flood	Low : Very high	0.49	0.27	1.00	3.52e-02	498
Likelihood Econ. loss	Flood	Medium : Very high	0.53	0.27	1.12	8.72e-02	712
Likelihood Econ. loss	Flood	High : Very high	0.55	0.29	1.15	1.00e-01	533
Likelihood Econ. loss	Storm	Low : Very high	0.90	0.43	1.86	7.82e-01	550
Likelihood Econ. loss	Storm	Medium : Very high	0.60	0.30	1.21	1.65e-01	802
Likelihood Econ. loss	Storm	High : Very high	0.49	0.19	1.08	1.18e-01	644
Likelihood Econ. loss	Landslide	Low : Very high	0.44	0.13	1.53	1.86e-01	25
Likelihood Econ. loss	Landslide	Medium : Very high	2.92	0.38	12.89	2.37e-01	40
Likelihood Econ. loss	Landslide	High : Very high	0.85	0.11	3.56	8.52e-01	24
Likelihood Econ. loss	Wildfire	Low : Very high	9.03	0.01	15400.1[illegible]	[illegible].09e-01	88
Likelihood Econ. loss	Wildfire	Medium : Very high	2.18	0.04	9.41	5.25e-01	97
Likelihood Econ. loss	Wildfire	High : Very high	0.27	0.11	0.66	3.43e-03	100
Likelihood Econ. loss	Drought	Low : Very high	2.31	0.45	21.66	4.12e-01	20
Likelihood Econ. loss	Drought	Medium : Very high	9.62	0.80	93.65	5.15e-02	25
Likelihood Econ. loss	Drought	High : Very high	1.54	0.26	13.32	6.76e-01	25

Odds ratios (OR) represent the likelihood of economic losses in each sHDI group relative to Very high sHDI regions (reference group). ORs are calculated using economic loss rates (economic losses in percent of exposed gross domestic product GDP in constant 2011 US$). Impact data are from the Emergency Events Database (EM-DAT) for climate-related disasters occurring between 1990 and 2020. The four sHDI groups follow the United Nations Development Programme thresholds: low (sHDI < 0.55), medium ($0.55 \leq$ sHDI < 0.70), high ($0.70 \leq$ sHDI < 0.80), and very high (sHDI ≥ 0.80). CI: 95% confidence interval estimated by bootstrapping the logistic regression coefficients (5,000 iterations). npoints: number of observations used in the logistic regression model.

Supplementary Table 20: Results from logistic regression analysis to estimate odds ratios of the likelihood of being affected per subnational Human Development Index (sHDI) group, deviation from national Human Development Index (HDI), and disaster type.

Impact	Disaster type	Case: sHDI group & deviation	Odds ratio	CI low	CI high	*p*-value	npoints
Likelihood affected	All types	Low, better-off : Very high	2.29	1.45	3.52	2.31e-04	1411
Likelihood affected	All types	Low, nat-average : Very high	3.05	2.21	4.40	1.65e-10	1857
Likelihood affected	All types	Low, worst-off : Very high	4.63	3.31	6.67	4.00e-18	1656
Likelihood affected	All types	Medium, better-off : Very high	1.67	1.07	2.61	2.35e-02	1557
Likelihood affected	All types	Medium, nat-average : Very high	2.47	1.81	3.53	1.15e-07	2133
Likelihood affected	All types	Medium, worst-off : Very high	3.12	2.12	4.67	7.38e-09	1565
Likelihood affected	All types	High, better-off : Very high	1.36	0.73	2.33	2.91e-01	1396
Likelihood affected	All types	High, nat-average : Very high	1.60	1.11	2.38	1.44e-02	1867
Likelihood affected	All types	High, worst-off : Very high	2.01	0.96	3.69	4.15e-02	1247
Likelihood affected	Flood	Low, better-off : Very high	2.69	1.53	4.87	8.57e-04	681
Likelihood affected	Flood	Low, nat-average : Very high	4.55	2.89	7.63	6.62e-10	978
Likelihood affected	Flood	Low, worst-off : Very high	6.01	3.85	10.03	1.65e-13	853
Likelihood affected	Flood	Medium, better-off : Very high	2.42	1.26	4.46	6.56e-03	774
Likelihood affected	Flood	Medium, nat-average : Very high	3.69	2.35	6.14	9.52e-08	1074
Likelihood affected	Flood	Medium, worst-off : Very high	5.45	3.24	9.51	5.05e-10	753
Likelihood affected	Flood	High, better-off : Very high	2.57	1.08	5.42	2.16e-02	628
Likelihood affected	Flood	High, nat-average : Very high	2.08	1.28	3.53	4.91e-03	964
Likelihood affected	Flood	High, worst-off : Very high	2.73	1.14	5.87	1.72e-02	578
Likelihood affected	Storm	Low, better-off : Very high	2.61	1.10	5.51	1.67e-02	504
Likelihood affected	Storm	Low, nat-average : Very high	2.18	1.11	4.17	2.08e-02	591
Likelihood affected	Storm	Low, worst-off : Very high	2.94	1.40	5.77	2.84e-03	539
Likelihood affected	Storm	Medium, better-off : Very high	1.83	0.90	3.53	8.26e-02	556
Likelihood affected	Storm	Medium, nat-average : Very high	2.32	1.39	4.13	2.53e-03	735
Likelihood affected	Storm	Medium, worst-off : Very high	2.65	1.30	5.19	4.80e-03	557
Likelihood affected	Storm	High, better-off : Very high	1.38	0.52	3.07	4.70e-01	533
Likelihood affected	Storm	High, nat-average : Very high	1.80	0.92	3.49	8.29e-02	624
Likelihood affected	Storm	High, worst-off : Very high	2.25	0.13	6.77	3.75e-01	456

Odds ratios (OR) represent the likelihood of affected people in each sHDI group and deviation from HDI, relative to Very high sHDI regions (reference group). ORs are calculated using affected rates (number of affected people per exposed population). Impact data are from the Emergency Events Database (EM-DAT) for climate-related disasters occurring between 1990 and 2020. The four sHDI groups follow the United Nations Development Programme thresholds: low (sHDI < 0.55), medium ($0.55 \leq$ sHDI < 0.70), high ($0.70 \leq$ sHDI < 0.80), and very high (sHDI ≥ 0.80). The deviation is defined as sHDI minus national HDI in the event year. Regions are classified, based on the distribution of deviations across all impacted regions, as worse-off ($\leq$20th percentile), national-average (between the 20th and 80th percentiles), or better-off ($\geq$80th percentile). CI: 95% confidence interval estimated by bootstrapping the logistic regression coefficients (5,000 iterations). npoints: number of observations used in the logistic regression model.

Supplementary Table 21: Results from logistic regression analysis to estimate odds ratios of the likelihood of fatality per subnational Human Development Index (sHDI) group, deviation from national Human Development Index (HDI), and disaster type.

Impact	Disaster type	Case	Odds ratio	CI low	CI high	*p*-value	npoints
Likelihood fatalities	All types	Low, better-off : Very high	2.27	0.82	6.16	1.12e-01	1426
Likelihood fatalities	All types	Low, nat-average : Very high	2.38	0.96	6.23	7.68e-02	1885
Likelihood fatalities	All types	Low, worst-off : Very high	1.22	0.54	2.97	6.65e-01	1626
Likelihood fatalities	All types	Medium, better-off : Very high	1.03	0.43	2.69	9.47e-01	1628
Likelihood fatalities	All types	Medium, nat-average : Very high	1.39	0.53	3.75	5.12e-01	2086
Likelihood fatalities	All types	Medium, worst-off : Very high	2.50	0.97	6.64	6.49e-02	1617
Likelihood fatalities	All types	High, better-off : Very high	1.36	0.37	4.11	6.21e-01	1435
Likelihood fatalities	All types	High, nat-average : Very high	0.92	0.32	2.51	8.72e-01	1787
Likelihood fatalities	All types	High, worst-off : Very high	3.30	0.88	9.63	5.11e-02	1253
Likelihood fatalities	Flood	Low, better-off : Very high	2.77	1.10	6.11	1.92e-02	520
Likelihood fatalities	Flood	Low, nat-average : Very high	2.79	1.30	5.77	7.08e-03	809
Likelihood fatalities	Flood	Low, worst-off : Very high	3.40	1.35	8.14	9.88e-03	665
Likelihood fatalities	Flood	Medium, better-off : Very high	2.13	0.95	4.45	5.56e-02	635
Likelihood fatalities	Flood	Medium, nat-average : Very high	2.22	1.16	4.27	1.60e-02	858
Likelihood fatalities	Flood	Medium, worst-off : Very high	5.80	1.33	16.93	1.67e-02	600
Likelihood fatalities	Flood	High, better-off : Very high	3.93	0.70	10.60	5.11e-02	489
Likelihood fatalities	Flood	High, nat-average : Very high	1.82	0.64	4.35	2.34e-01	725
Likelihood fatalities	Flood	High, worst-off : Very high	2.74	0.75	6.42	6.40e-02	418
Likelihood fatalities	Storm	Low, better-off : Very high	2.74	1.31	6.53	1.20e-02	606
Likelihood fatalities	Storm	Low, nat-average : Very high	12.72	1.19	41.39	5.39e-03	698
Likelihood fatalities	Storm	Low, worst-off : Very high	4.96	1.93	12.44	7.94e-04	631
Likelihood fatalities	Storm	Medium, better-off : Very high	0.68	0.30	1.64	3.61e-01	657
Likelihood fatalities	Storm	Medium, nat-average : Very high	1.04	0.49	2.40	9.20e-01	816
Likelihood fatalities	Storm	Medium, worst-off : Very high	5.68	1.29	17.04	9.89e-03	662
Likelihood fatalities	Storm	High, better-off : Very high	0.36	0.15	0.86	2.09e-02	631
Likelihood fatalities	Storm	High, nat-average : Very high	0.34	0.17	0.78	4.73e-03	690
Likelihood fatalities	Storm	High, worst-off : Very high	1.57	0.39	4.64	4.78e-01	552

Odds ratios (OR) represent the likelihood of fatalities in each sHDI group and deviation from HDI, relative to Very high sHDI regions (reference group). ORs are calculated using fatality rates (number of fatalities per exposed population). Impact data are from the Emergency Events Database (EM-DAT) for climate-related disasters occurring between 1990 and 2020. The four sHDI groups follow the United Nations Development Programme thresholds: low (sHDI < 0.55), medium ($0.55 \leq$ sHDI < 0.70), high ($0.70 \leq$ sHDI < 0.80), and very high (sHDI ≥ 0.80). The deviation is defined as sHDI minus national HDI in the event year. Regions are classified, based on the distribution of deviations across all impacted regions, as worse-off ($\leq$20th percentile), national-average (between the 20th and 80th percentiles), or better-off ($\geq$80th percentile). CI: 95% confidence interval estimated by bootstrapping the logistic regression coefficients (5,000 iterations). npoints: number of observations used in the logistic regression model.

Supplementary Table 22: Results from logistic regression analysis to estimate odds ratios of the likelihood of economic loss per subnational Human Development Index (sHDI) group, deviation from national Human Development Index (HDI), and disaster type.

Impact	Disaster type	Case	Odds ratio	CI low	CI high	*p*-value	npoints
Likelihood Econ. loss	All types	Low, better-off : Very high	0.46	0.22	0.86	3.03e-02	894
Likelihood Econ. loss	All types	Low, nat-average : Very high	0.53	0.32	0.86	1.16e-02	1016
Likelihood Econ. loss	All types	Low, worst-off : Very high	0.92	0.34	1.88	8.53e-01	964
Likelihood Econ. loss	All types	Medium, better-off : Very high	0.56	0.20	1.22	2.18e-01	1010
Likelihood Econ. loss	All types	Medium, nat-average : Very high	0.61	0.35	1.02	7.05e-02	1328
Likelihood Econ. loss	All types	Medium, worst-off : Very high	0.82	0.33	1.56	6.09e-01	1036
Likelihood Econ. loss	All types	High, better-off : Very high	0.30	0.12	0.59	2.71e-03	968
Likelihood Econ. loss	All types	High, nat-average : Very high	0.46	0.25	0.81	9.85e-03	1176
Likelihood Econ. loss	All types	High, worst-off : Very high	0.91	0.37	1.80	8.16e-01	883
Likelihood Econ. loss	Flood	Low, better-off : Very high	0.63	0.16	1.71	4.48e-01	309
Likelihood Econ. loss	Flood	Low, nat-average : Very high	0.47	0.25	0.96	3.10e-02	385
Likelihood Econ. loss	Flood	Low, worst-off : Very high	0.47	0.21	1.03	5.67e-02	362
Likelihood Econ. loss	Flood	Medium, better-off : Very high	1.14	0.26	3.42	8.44e-01	355
Likelihood Econ. loss	Flood	Medium, nat-average : Very high	0.47	0.24	0.95	2.69e-02	535
Likelihood Econ. loss	Flood	Medium, worst-off : Very high	0.23	0.09	0.55	1.65e-03	380
Likelihood Econ. loss	Flood	High, better-off : Very high	0.24	0.08	0.58	3.53e-03	329
Likelihood Econ. loss	Flood	High, nat-average : Very high	0.54	0.26	1.15	1.00e-01	458
Likelihood Econ. loss	Flood	High, worst-off : Very high	1.33	0.25	3.62	6.75e-01	304
Likelihood Econ. loss	Storm	Low, better-off : Very high	0.44	0.19	0.92	4.28e-02	459
Likelihood Econ. loss	Storm	Low, nat-average : Very high	0.88	0.37	1.87	7.55e-01	493
Likelihood Econ. loss	Storm	Low, worst-off : Very high	1.29	0.31	3.65	6.97e-01	468
Likelihood Econ. loss	Storm	Medium, better-off : Very high	0.17	0.09	0.33	1.29e-07	524
Likelihood Econ. loss	Storm	Medium, nat-average : Very high	0.34	0.16	0.70	3.84e-03	632
Likelihood Econ. loss	Storm	Medium, worst-off : Very high	1.77	0.58	4.10	2.45e-01	516
Likelihood Econ. loss	Storm	High, better-off : Very high	0.40	0.08	1.01	1.51e-01	503
Likelihood Econ. loss	Storm	High, nat-average : Very high	0.54	0.13	1.44	3.23e-01	561
Likelihood Econ. loss	Storm	High, worst-off : Very high	0.46	0.14	1.06	1.30e-01	450

Odds ratios (OR) represent the likelihood of economic losses in each sHDI group and deviation from HDI, relative to Very high sHDI regions (reference group). ORs are calculated using economic loss rates (economic losses per exposed gross domestic product GDP in constant 2011 US$). Impact data are from the Emergency Events Database (EM-DAT) for climate-related disasters occurring between 1990 and 2020. The four sHDI groups follow the United Nations Development Programme thresholds: low (sHDI < 0.55), medium ($0.55 \leq$ sHDI < 0.70), high ($0.70 \leq$ sHDI < 0.80), and very high (sHDI ≥ 0.80). The deviation is defined as sHDI minus national HDI in the event year. Regions are classified, based on the distribution of deviations across all impacted regions, as worse-off ($\leq$20th percentile), national-average (between the 20th and 80th percentiles), or better-off ($\geq$80th percentile). CI: 95% confidence interval estimated by bootstrapping the logistic regression coefficients (5,000 iterations). npoints: number of observations used in the logistic regression model.

Supplementary Table 23: EM-DAT disaster type classification (left), and the disaster classification used in this study (right).

EM-DAT classification	Adopted classification
Flood	Flood
Storm	Storm
Mass movement (wet)	Landslide
Mass movement (dry)	Landslide
Extreme temperature (Cold wave)	Cold wave
Extreme temperature (Severe winter conditions)	Cold wave
Extreme temperature (Heat wave)	Heat wave
Wildfire	Wildfire
Drought	Drought

The EM-DAT classification uses detailed subcategories for certain disaster types. For analytical consistency, we consolidated these into broader categories: both wet and dry mass movements are classified as landslides, and extreme temperature events (cold waves, severe winter conditions, and heat waves) are categorized according to their hazard dynamics. This consolidation enables robust statistical analysis while maintaining physically meaningful distinctions between disaster types. Event counts per disaster type are provided in Supplementary Table 1.
