# Supplementary material for: Inequality in human development amplifies climate-related disaster risk
Source: Nat Commun. 2026 Jun 17;17:5067. doi: 10.1038/s41467-026-73873-9 (PMC13276189; doi:10.1038/s41467-026-73873-9)
Supplement: Supplementary file 2 — Transparent Peer Review file [file 41467_2026_73873_MOESM2_ESM.pdf]

nature portfolio

Peer Review File

Inequality in human development amplifies climate-related disaster risk

Corresponding Author: Mr Khalil Teber

This manuscript has been previously reviewed at another journal. This document only contains information relating to versions considered at Nature Communications.

This file contains all reviewer reports in order by version, followed by all author rebuttals in order by version.

Version 0:

Reviewer comments:

Reviewer #1

(Remarks to the Author)
I am satisfied with the revisions made, therefore I can recommend publication at this stage.

(Remarks on code availability)
The authors present a well-developed set of code, accompanied by clear and thorough inline comments that greatly improve readability. The underlying logic is consistent with the intended objectives. GeoNames requires an individual account but the authors have provided all the supplementary files.

Reviewer #2

(Remarks to the Author)
Based on the subnational Human Development Index (sHDI) and global climate-related disaster data, this study systematically analyzes the driving role of human development in shaping disaster impact patterns and the exacerbating effect of within-country sHDI inequality on disaster risk during the period 1990–2020. In general, this manuscript has a clear logic and well-written. However, there are still some aspects of the manuscript that need improvement:
1.This study explores the impacts of disasters with different severity levels on different human development groups during 1990–2020. Considering that the human development group of a region may change over a long time series, supplementing the dynamic evolution paths of human development index and disaster impacts in representative regions can further enhance the depth of the study. Does the improvement of regional sHDI significantly reduce its disaster impact rate? Is there regional heterogeneity or time lag effect (e.g., the human development index takes years to show its effect)?
2.Disaster intensity is not controlled in the analysis of Result 2.3, which may lead to the confounding of impact differences among different development groups with the inherent intensity heterogeneity of disasters. Can disaster intensity be classified according to disaster intensity indicators (such as precipitation, maximum wind speed, etc.)? Under similar disaster intensity, the disaster impacts of different sHDI groups should be considered.
3.In Method 4.2.1, the Superposed Epoch Analysis (SEA) method uniformly uses "6-year daily-scale climate data and a 1-year window before and after the event" to calculate composite anomalies for all disaster types, without distinguishing the temporal heterogeneity of different disasters. For example, disasters such as droughts may last for several years, while storms, floods and other disasters are mostly short-term events. A unified time window may lead to excessive dilution of the climate background data for long-duration disaster types, or interference of key anomaly data of short-term disasters by redundant data. Please provide the reference sources for the setting of the time window or verify the impact of window adjustment on the calculation results of different disaster types.
4.The source of the sHDI data provided in the paper is Reference 27, and the time series of the sHDI data given in this reference seems to be 1990–2017, rather than 1990–2020 as claimed in this study. Please confirm whether the time series are consistent; if not, please explain how the missing part is supplemented.
5.The current discussion section covers multiple topics. It is suggested to add subheadings to enhance readability and logical organization.
6.In Figure 1a, colors represent different sHDI levels, but do the shades of colors correspond to other variables? Please supplement the legend.

(Remarks on code availability)
The code is appropriate and provides a README file.

Reviewer #3

(Remarks to the Author)
Thank you very much for the opportunity to review the revised/transferred. I understand that the authors defend the use of EM-DAT being the only global dataset with no additional analysis to explore the known areas of limitation. I am fine with this approach as long as the discussions of alternative datasets suggested (e.g. Desinvestar, satellite) etc are explored sufficiently in the main text as further avenues to validate the findings etc. Given the paper's interest to explore the socioeconomic vulnerabilities, I think a meaningful additional analysis can be done for those sub-set of countries for which data is known to be sparse. This was the spirit of my previous comments and the author chooses not to, which is fine. But the idea of scientific contribution is to advance our knowledge and not to continue defending the limited approach just because many other studies have done them.

In terms of terminology, I appreciate the author's clarification on hazard intensity. The manuscript is now clear for the general scientific audience who will be following a more conventional framing of hazard, exposure vulnerability as adopted by IPCC. On terminology, as much as possible, please refrain from including in-precise use of terms such as 'humanitarian disasters'. 'affected population (requiring humanitarian assistance)'. Please align as much to IPCC or PDNA/DALA, or other internationally accepted glossaries such as those of UNDRR. The affected population are simply affected population, if they require humanitarian assistance as such, for example, is a function of another dimension such as their local coping capacities. As the focus of this paper is on social vulnerability of disasters, for which well-established strands of literature exist. Please pay due attention to those and avoid introducing new terminology/concept that will confuse the readers.

(Remarks on code availability)
N.A.

Version 1:

Reviewer comments:

Reviewer #2

(Remarks to the Author)
The reviewers have enhanced the rigour of the research and the clarity of its presentation. The findings demonstrate clear innovation and academic contribution. Prior to publication, the following details require refinement:
1.In the caption of Figure 1a, it is suggested to clarify that "point density reflects only the number of events, and the apparent clustering results from spatial overlap of points, rather than representing any additional variable."
2.Regarding the sentence "To limit spatial uncertainty, we excluded events with a total area larger than one million square km," it is advisable to provide literature support for the selection of this threshold.

(Remarks on code availability)

Open Access This Peer Review File is licensed under a Creative Commons Attribution 4.0 International License, which permits use, sharing, adaptation, distribution and reproduction in any medium or format, as long as you give appropriate credit to the original author(s) and the source, provide a link to the Creative Commons license, and indicate if changes were made.
In cases where reviewers are anonymous, credit should be given to 'Anonymous Referee' and the source.
The images or other third party material in this Peer Review File are included in the article's Creative Commons license, unless indicated otherwise in a credit line to the material. If material is not included in the article's Creative Commons license and your intended use is not permitted by statutory regulation or exceeds the permitted use, you will need to obtain permission directly from the copyright holder.
To view a copy of this license, visit https://creativecommons.org/licenses/by/4.0/

Dear Editors:

Many thanks for handling the manuscript. Please find below our point by point responses to the reviewer comments. Our replies are provided in blue after each comment. Line numbers used in this document correspond to the manuscript version with track changes. New figures produced in response to reviewer comments have been assigned their final numbering within the Supplementary Information, ensuring consistent referencing between the manuscript, the responses, and the supplementary file.

Reviewers Comments:

Reviewer #1 (Remarks to the Author):

I am satisfied with the revisions made, therefore I can recommend publication at this stage.

We thank the reviewer for the positive recommendation and we are pleased that all concerns are resolved. We appreciate the constructive feedback provided through all of the review process, which helped improve the manuscript in clarity and robustness. We acknowledge the importance of your comments in the new manuscript.

Reviewer #1 (Remarks on code availability):

The authors present a well-developed set of code, accompanied by clear and thorough inline comments that greatly improve readability. The underlying logic is consistent with the intended objectives. GeoNames requires an individual account but the authors have provided all the supplementary files.

We thank the reviewer for the positive assessment of the code availability and documentation.

Reviewer #2 (Remarks to the Author):

Based on the subnational Human Development Index (sHDI) and global climate-related disaster data, this study systematically analyzes the driving role of human development in shaping disaster impact patterns and the exacerbating effect of within-country sHDI inequality on disaster risk during the period 1990–2020. In general, this manuscript has a clear logic and well-written. However, there are still some aspects of the manuscript that need improvement:

We would like to thank the reviewer for the careful and positive assessment of our paper. Please find below our answers and the details of our revisions based on your comments and suggestions. We acknowledge the importance of your comments in the new manuscript.

1.This study explores the impacts of disasters with different severity levels on different human development groups during 1990–2020. Considering that the human development group of a region may change over a long time series, supplementing the dynamic evolution paths of human development index and disaster impacts in representative regions can further enhance the depth of the study. Does the improvement of regional sHDI significantly reduce its disaster impact rate? Is there regional heterogeneity or time lag effect (e.g., the human development index takes years to show its effect)?

We thank the reviewer for this insightful comment. Our answer addresses (i) the development–impact relationship, (ii) long-term co-evolution, (iii) regional heterogeneity, and (iv) potential lag effects.

Relationship between human development and impact rates
To complement the global patterns shown Fig. 4 which establishes a robust negative association between subnational human development and disaster impacts at the global scale, we now computed continent-level scatterplots showing the relationship between event-level sHDI and normalized impacts (Fig. S12). These stratified results confirm that the negative development–impact gradient holds across continents and impact types, strengthening the generality of the finding:

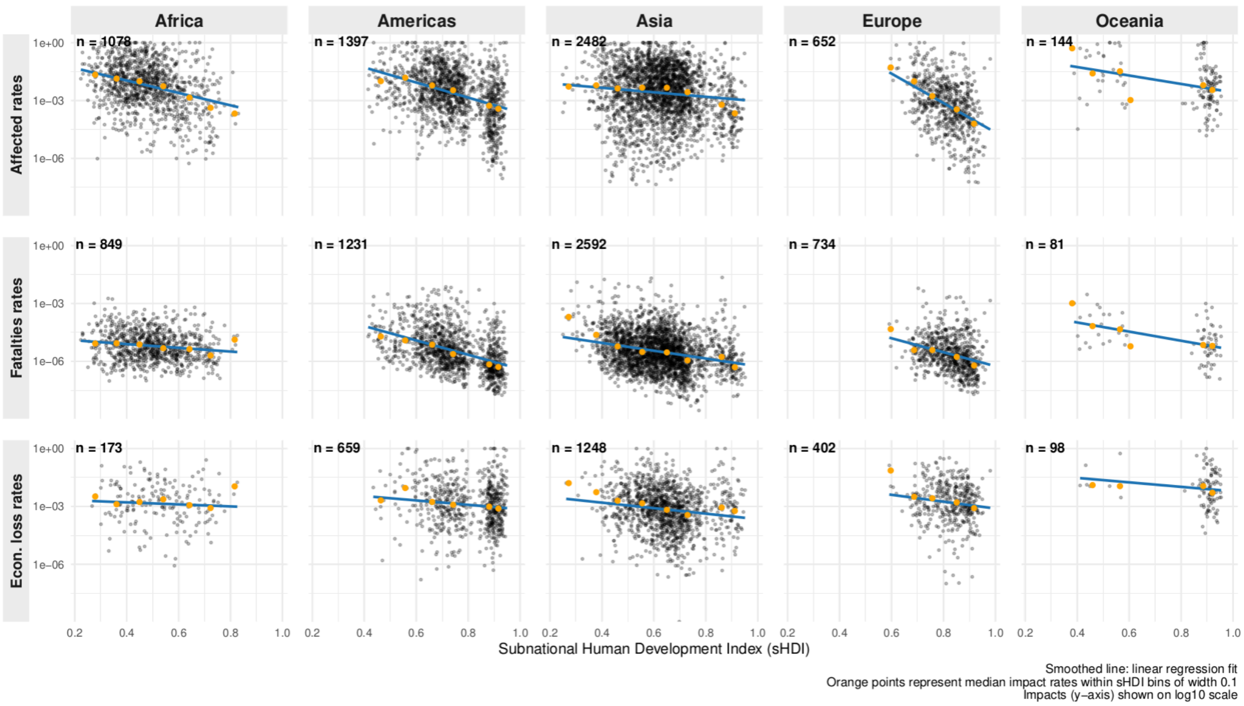

Figure S12. Relationship between subnational Human Development Index (sHDI) and disaster impact rates across continents (1990–2020). Each panel shows impact rates (normalized affected-population rates, fatality rates, and economic loss rates) as functions of sHDI for disaster-impacted regions. Regions are grouped by continent, with clusters illustrating how impact severity decreases with increasing sHDI, but with substantial intra-continental heterogeneity. In three continents, we see a divide between a cluster of highly developed regions (sHDI ≥ 0.85) and the rest—formed by Australia and New Zealand in Oceania; Japan, South Korea, Saudi Arabia, and T¨urkiye in Asia; and the United States, Canada, Chile, and Argentina in the Americas.

Dynamic evolution of human development

We already document long-term improvements in development conditions globally and across development groups (Fig. 3, Fig. S6, Fig. S7, Fig. S8, Fig. S10). To provide the temporal dimension requested by the reviewer, we added continent-specific 5-year binned trajectories of sHDI for disaster-affected regions (Figs. S14–S16). These new figures clearly show the consistent, monotonic increase in sHDI across all continents, indicating long-term structural improvements independent of short-term impact variability.

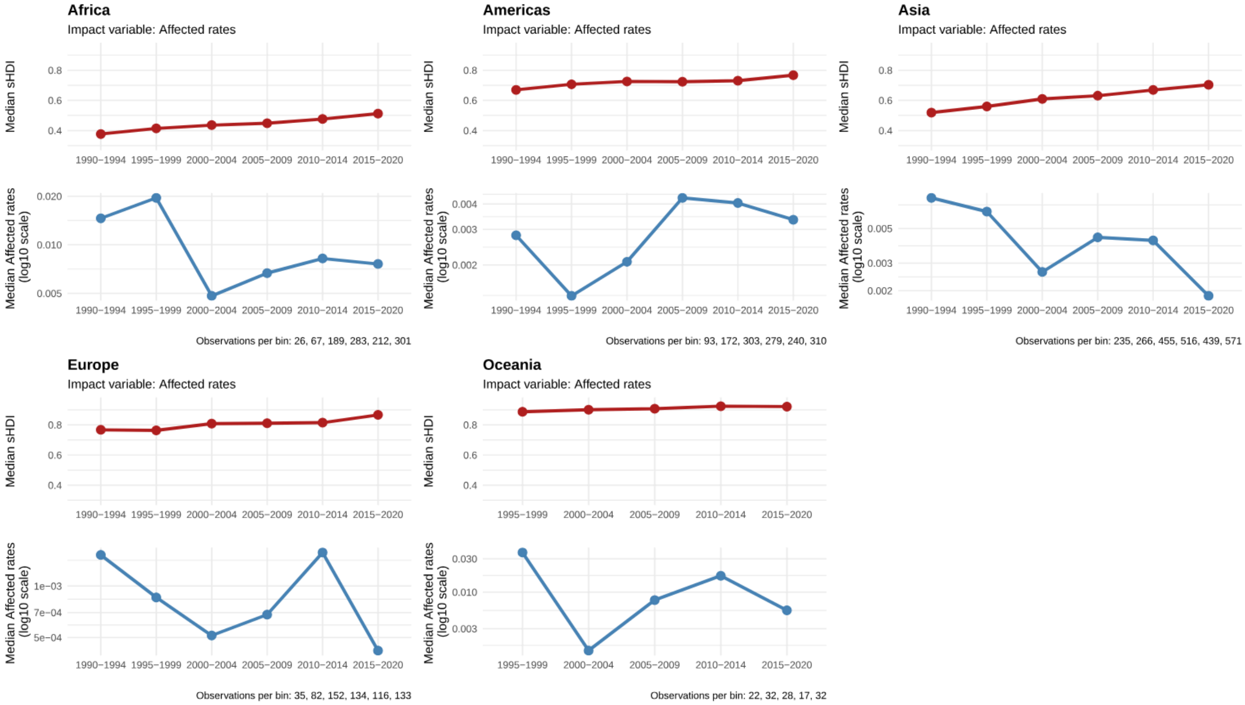

Figure S14. Temporal co-evolution of subnational Human Development Index (sHDI) and affected rates across continents (1990–2020). Median sHDI and median affected rates are computed over 5-year time bins for each continent (bins with fewer than 20 events excluded). The upper panels show long-term increases in sHDI, while the lower panels show corresponding changes in affected rates (log10 scale). Event counts per bin are reported below each panel.

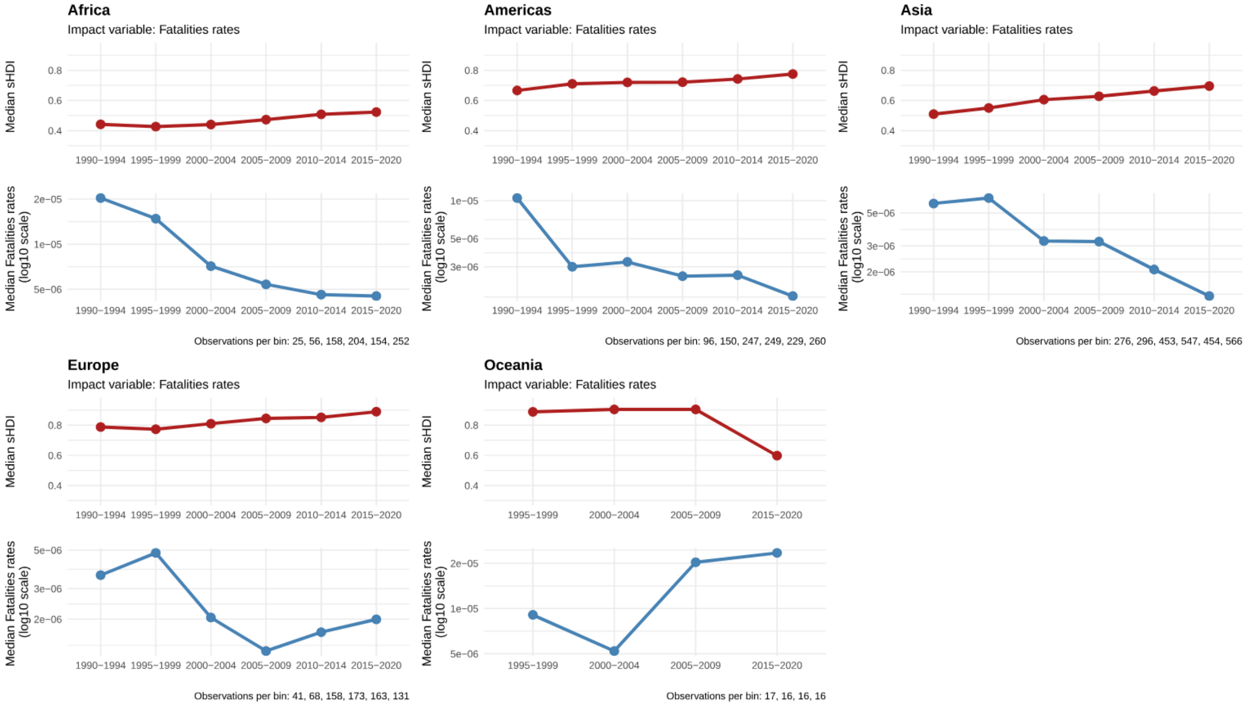

Figure S15. Temporal co-evolution of subnational Human Development Index (sHDI) and fatalities rates across continents (1990–2020). Median sHDI and median fatalities rates are computed over 5-year time bins for each continent (bins with fewer than 20 events excluded). Fatality rates show marked long-term reductions in several continents, paralleling increases in sHDI. Impacts are shown on a log10 scale. Event counts per bin are reported below each panel. The apparent decline in median sHDI for Oceania for 2015-2020 reflects the events with sufficient fatality data in this region in the recent years that occurred predominantly in lower-development countries (Papua New Guinea and Vanuatu), which dominated the available observations of the continent.

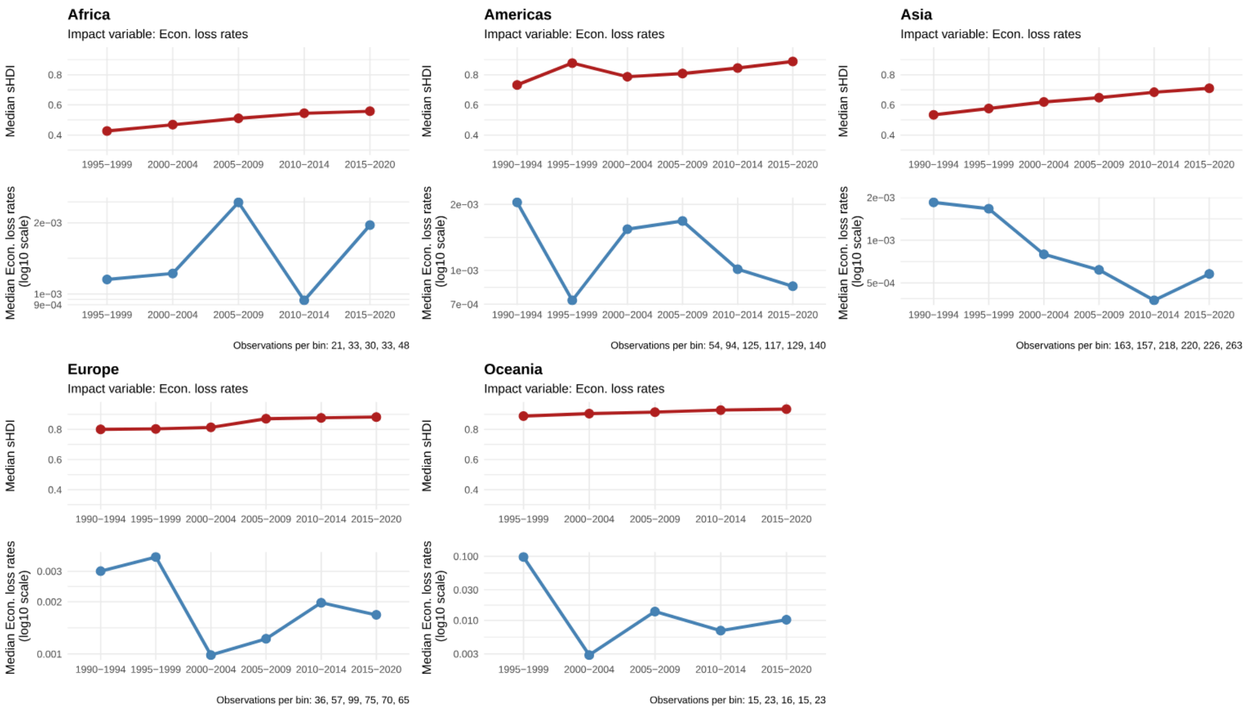

Figure S16. Temporal co-evolution of subnational Human Development Index (sHDI) and economic loss rates across continents (1990–2020). Median sHDI and median economic loss rates are calculated for each 5-year time bin (bins with fewer than 20 events excluded). Economic loss rates are shown on a log10 scale. Temporal patterns exhibit greater variability due to sparse and heterogeneous reporting of economic loss data in EM-DAT and the heavy-tailed nature of disaster economic losses. Event counts for each bin are shown below the panels.

Dynamic evolution of disaster impacts

Fig. 4 shows global decline in normalized fatalities and stabilization or decline of declines in other impact indicators over 1990–2020. To mirror the development trajectories, we now additionally compute median impacts within the same 5-year at the continental scale (Supplementary Figs. S14–S16). These patterns corroborate the global declines in fatalities rates and stabilize or decline in other impact indicators.

Regional heterogeneity

The continental level scatterplots and temporal trajectories reveal regional differences: Africa shows a steep decline in human losses (affected and fatalities) but the median impact rates remain among the highest; Asia and the Americas show moderate but persistent reductions; and Europe shows smaller declines of human losses fractions. For normalized economic losses, all continents except Africa show weak but consistent declines. These differences align with variations in hazard exposure, reporting practices, type of hazards occurring, and development pathways across regions.

Potential time lag effects

The main text explains that sHDI evolves slowly, whereas disaster impacts fluctuate from year to year. When aggregating events into multi-year bins, meaningful “lag-like” patterns emerge: improvements in development correspond to reductions in impacts at multi-decadal timescales. To ensure clarity, we now reference these supplementary analyses explicitly in two locations in the main text:

L198-200: “This negative association between human development and disaster impacts holds consistently across continents and across all impact variables (Fig. S12)”.

L244-247: “Consistent with these global patterns, the continent-level co-evolution trajectories of sHDI and disaster impacts also indicate gradual improvements in development alongside declining impact rates (Figs. S14–S16).”

We hope that taken together, these additions strengthen and validate the global patterns while maintaining the sHDI-based analytical framework, which to our mind provides a more meaningful socioeconomic structure than geopolitical regions.

We thank the reviewer for this valuable suggestion; the requested robustness analyses have been incorporated into the Supplementary Information and referenced in the main text.

2.Disaster intensity is not controlled in the analysis of Result 2.3, which may lead to the confounding of impact differences among different development groups with the inherent intensity heterogeneity of disasters. Can disaster intensity be classified according to disaster intensity indicators (such as precipitation, maximum wind speed, etc.)? Under similar disaster intensity, the disaster impacts of different sHDI groups should be considered.

We thank the reviewer for this remark. Please note that the manuscript already addresses the potential role of disaster intensity as confounding by disaster intensity at a descriptive level. Supplementary Fig. S18 shows that hazard-intensity anomalies (e.g., maximum daily precipitation (AMAX_tp), maximum daily windgust (AMAX_windgustmax), minimum daily surface moisture (AMIN_smsurf)) are comparable across sHDI development groups, and in several cases more intense in higher-sHDI regions. This indicates that the global development–impact gradients cannot be explained by systematic differences in mean hazard intensity.
We implemented an intensity-stratified comparison that holds hazard intensity approximately constant while varying development level. To do so, we applied the following procedure:

Hazard types eligible for stratification
Only hazard types with sufficient event counts across both intensity ranges and sHDI groups can support a valid joint stratification. After applying minimum sample-size constraints (≥20 events per sHDI group within an intensity bin, and at least two sHDI groups represented per bin), only floods and storms meet these conditions.

For the remaining hazard types (heat waves, cold waves, droughts, wildfires, landslides), the available observations collapse into bins containing a single sHDI group or too few events for stable comparisons. We therefore restrict stratified analyses to hazard types where inference is statistically defensible, as recommended in best practice for stratified impact modeling.

Stratification procedure
Events were classified into three and five intensity bins using the climate anomalies defined in the Methods:

- Floods: maximum daily precipitation anomaly (AMAX_tp)
- Storms: maximum daily wind-gust anomaly (AMAX_windgustmax)

We present the five-bin stratification (Figs. S21–S22) in the Supplementary Information, as it provides finer resolution while satisfying sample-size requirements. Both the three-bin and five-bin approaches yield consistent results.

Findings from intensity-controlled comparisons
Across all eligible hazard types and across all three impact metrics (affected population rates, fatalities rates, economic losses rates), the development–impact gradient remains monotonic and robust within each intensity bin.
In other words:

- At comparable hazard intensity, lower-sHDI regions consistently exhibit higher impact rates.
- Higher hazard intensity does not eliminate the vulnerability gradient.
- The stratified analysis confirms that differences in hazard intensity do not drive the global sHDI–impact disparities.

These results directly address the reviewer's concern and provide a strong causal consistency check: development-level differences remain predictive even after controlling for hazard severity.

Integration into manuscript
To ensure clarity, we added the following statement to the Methods:
L282-285: To assess whether these patterns persist when hazard intensity is held approximately constant, we further stratified flood and storm events into intensity bins. Across all comparable intensity ranges, the development–impact gradients remain evident (Figs. S21–S22).

L568-573: We additionally performed an intensity-stratified comparison for hazard types with sufficient event counts, to estimate how the development–impact differences could be

confounded by variations in hazard intensity. This analysis is reported in Supplementary Figs. S21–S22.

We thank the reviewer for this constructive suggestion, which substantially strengthened the analysis.

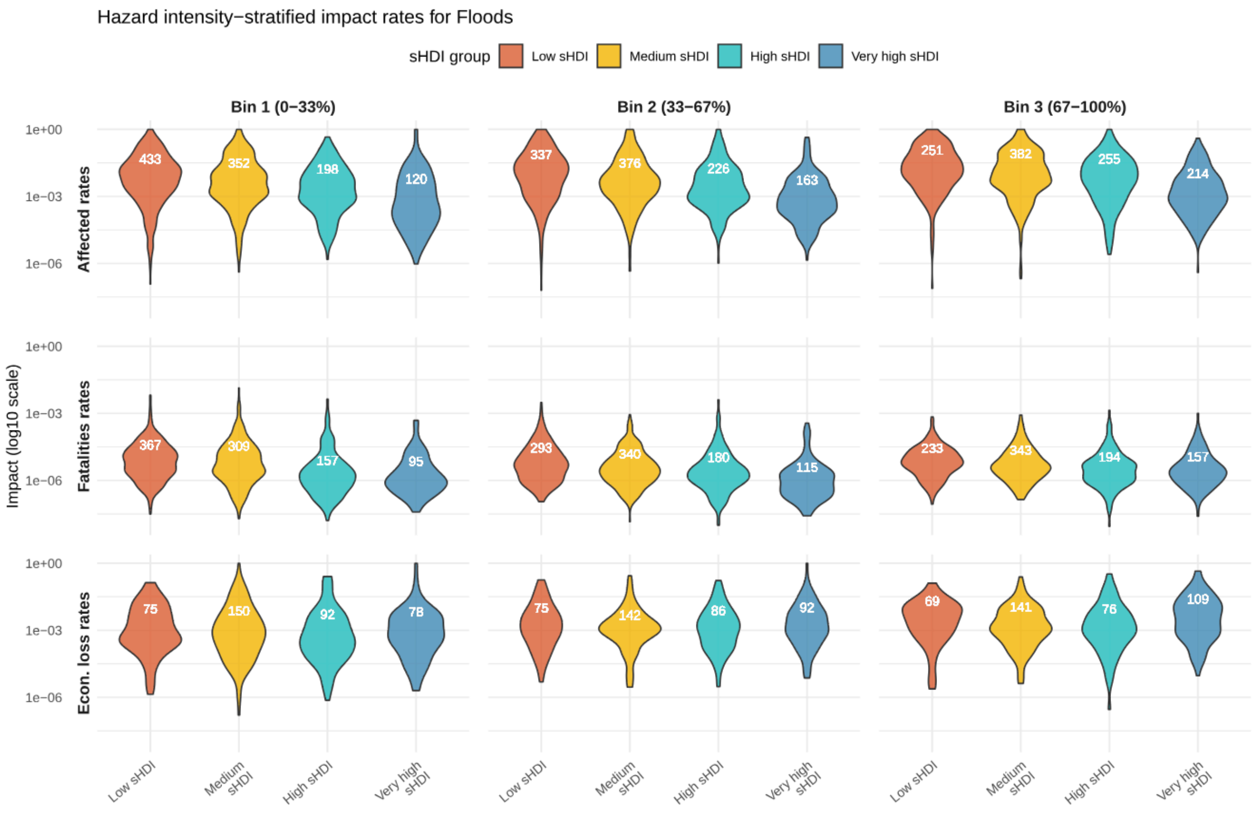

Figure S21A. *Intensity-stratified disaster impacts for floods (three-bin stratification).* *Events are grouped into terciles of flood intensity (estimated with maximum daily precipitation anomaly). For each bin, distributions of normalized affected population, normalized fatalities, and normalized damages (log$_{10}$ scale) are shown across sHDI development groups. Only sHDI groups with ≥20 events per bin are retained, and bins with fewer than two sHDI groups are excluded. Sample sizes are indicated above each distribution. Across all impact categories, lower-development regions exhibit systematically higher normalized impacts within comparable intensity ranges.*

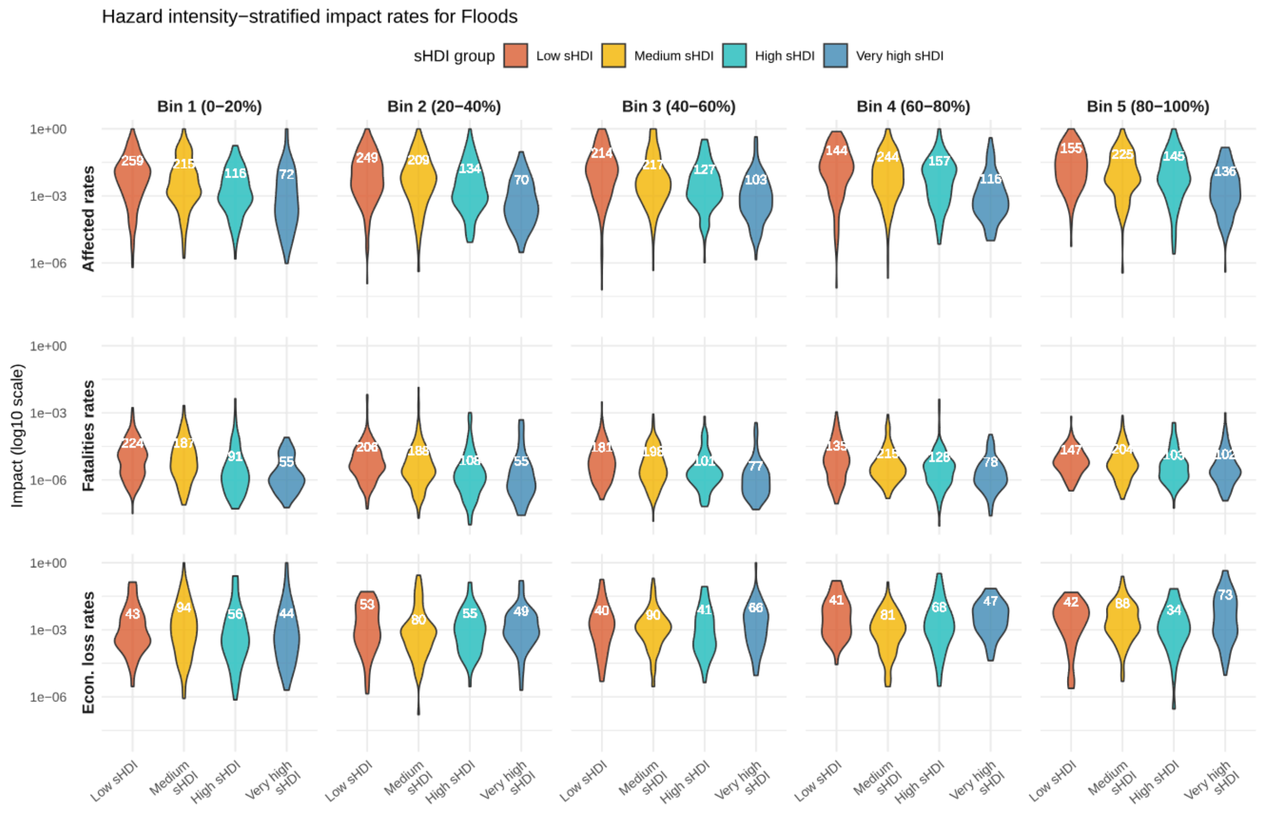

Supplementary Figure S21. *Fine-grained intensity stratification of flood impacts (five-bin stratification). Flood events are partitioned into quintiles of standardized intensity (0–20%, 20–40%, 40–60%, 60–80%, 80–100% of maximum daily precipitation anomalies). Violin plots show distributions of affected rates, normalized rates, and economic loss rates (log10 scale) for each sHDI group; labels denote the number of events retained after applying an n ≥ 20 threshold. Bins without at least two sHDI groups are removed. The separation between development groups persists across all intensity strata and impact dimensions, indicating that the development–impact gradient is not attributable to differences in flood intensity.*

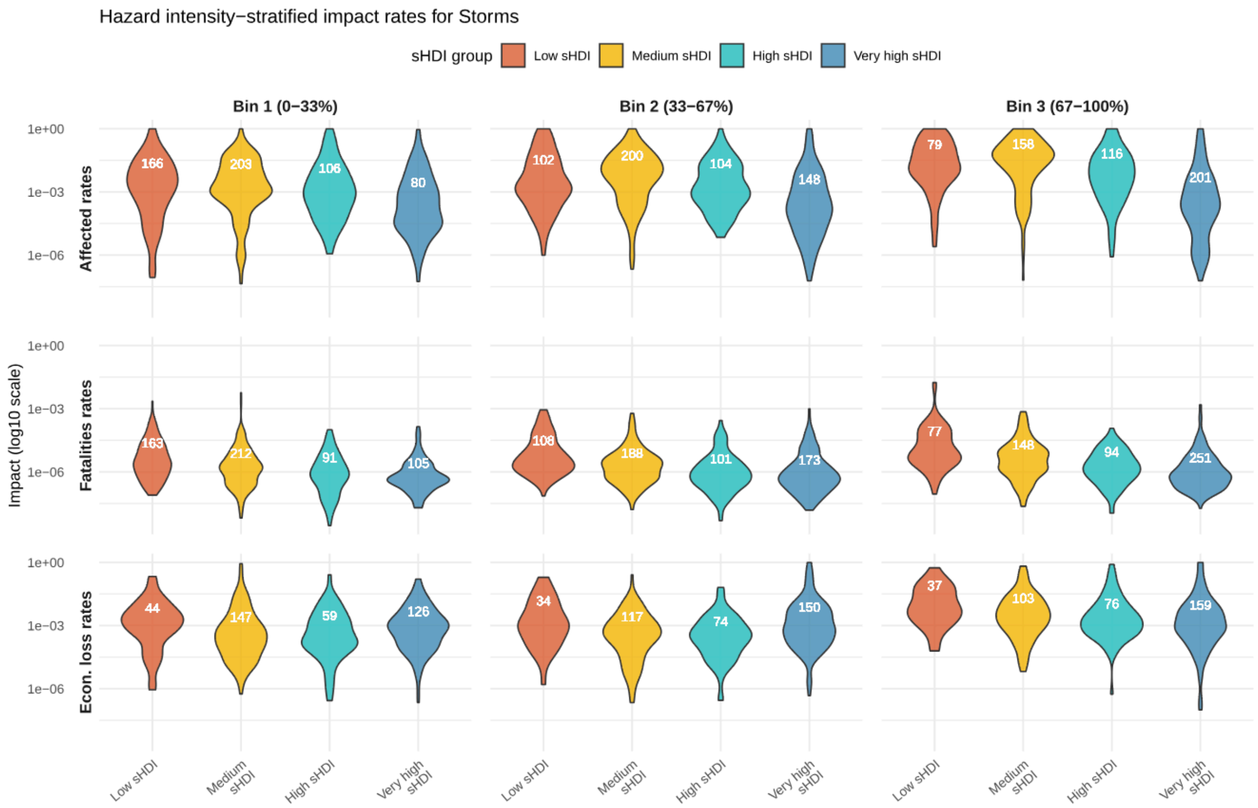

Figure S22A *Intensity-stratified disaster impacts for storms (three-bin stratification). Storm events are stratified into terciles of daily maximum wind-gust anomaly. Distributions of normalized affected population, normalized fatalities, and normalized damages (log_{10} scale) are shown for each sHDI category. Only sHDI groups with ≥20 observations per intensity bin are displayed, and bins with fewer than two sHDI groups are omitted. Sample sizes are indicated above each distribution. Impact levels consistently decline with increasing development even within matched storm-intensity ranges.*

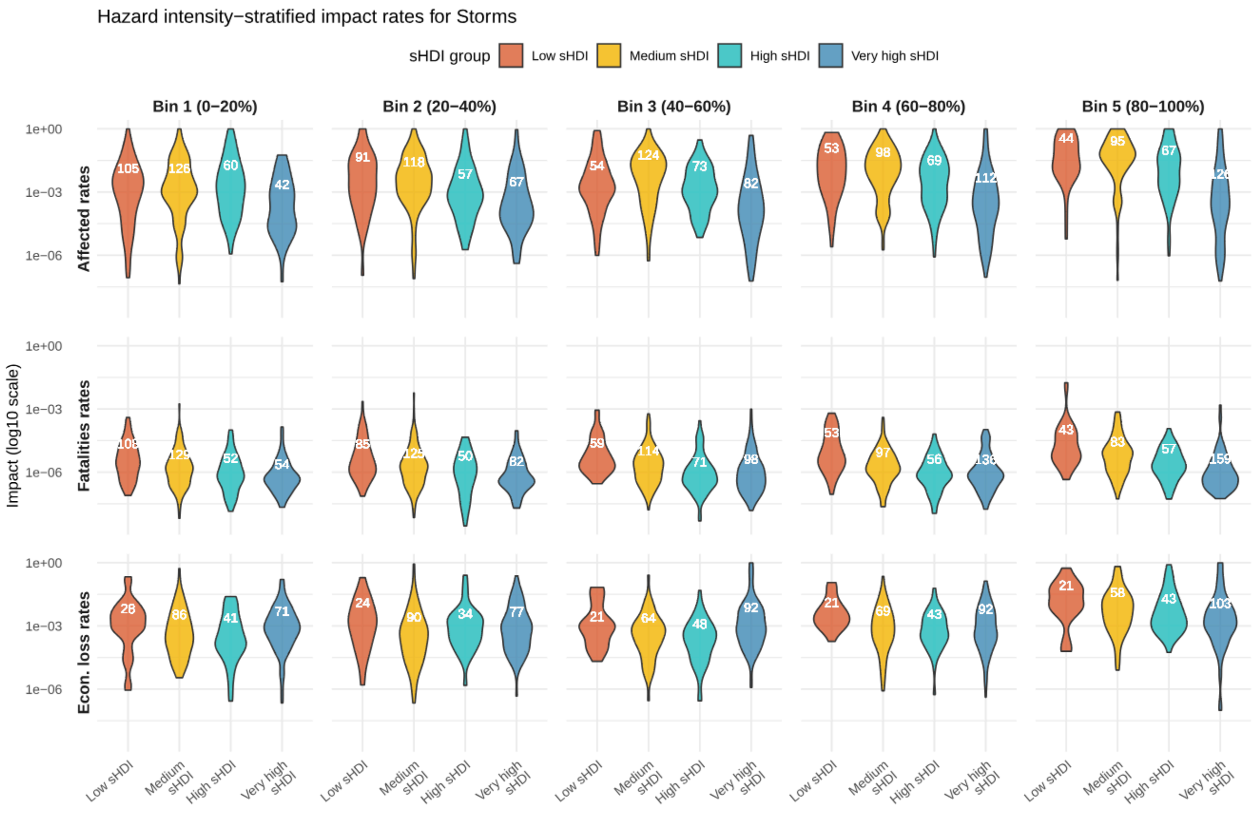

Supplementary Figure S22. *Fine-grained intensity stratification of storm impacts (five-bin stratification). Storm intensities are divided into quintiles (0–20%, 20–40%, 40–60%, 60–80%, 80–100% of maximum daily wind gust anomalies. Violin plots depict the distributions of affected rates, fatalities rates, and economic loss rates (log scale) across sHDI groups; event counts after filtering (n ≥ 20) are shown above each distribution. Bins lacking at least two development categories are excluded. The observed ordering of impacts across sHDI groups remains consistent across intensity quintiles, providing further evidence that differences in development conditions, rather than hazard intensity, underpin the observed variation in disaster impacts.*

3.In Method 4.2.1, the Superposed Epoch Analysis (SEA) method uniformly uses "6-year daily-scale climate data and a 1-year window before and after the event" to calculate composite anomalies for all disaster types, without distinguishing the temporal heterogeneity of different disasters. For example, disasters such as droughts may last for several years, while storms, floods and other disasters are mostly short-term events. A unified time window may lead to excessive dilution of the climate background data for long-duration disaster types, or interference of key anomaly data of short-term disasters by redundant data. Please provide the reference sources for the setting of the time window or verify the impact of window adjustment on the calculation results of different disaster types.

We thank the reviewer for raising this important methodological point. We clarify that the Superposed Epoch Analysis (SEA) is used exclusively for descriptive climatological context, and none of the statistical analyses or conclusions rely on SEA-derived metrics. Our choice of window therefore does not influence any analytical result.

The purpose of the Superposed Epoch Analysis (SEA) is to describe the climatological context of hazardous events (Fig. 5 ,Fig. S17). It is used solely to illustrate the typical temporal evolution of climate anomalies around reported event start dates, not to estimate event duration, not to define hazard windows, and not to compute any of the climate anomalies used in the main analyses. All statistical analyses (hazard–impact associations, stratification, correlations, etc.) employ event-level anomalies computed independently using a ±3-day window (Methods 4.2.2).

We employ a fixed multi-year window because this is standard practice in SEA applications in climate and climate-impact research, where events must be aligned at a common reference point (day 0). A uniform window ensures that differences across hazard types reflect the properties of the hazards themselves rather than arbitrary differences in analytical setup. This approach has been already used in the literature (https://www.nature.com/articles/nature16467#Abs3, https://link.springer.com/article/10.1007/s10584-019-02556-w).

Our composite climatological anomalies in Fig. S17 explicitly reveal the heterogeneity that the reviewer refers to:

- Fast-onset hazards (floods, storms, landslides) produce sharp, short-lived anomalies concentrated within a few days around day 0.
- Slow-onset hazards (heat waves, droughts, wildfires) exhibit persistent soil-moisture and temperature anomalies extending months before and after the reported onset.

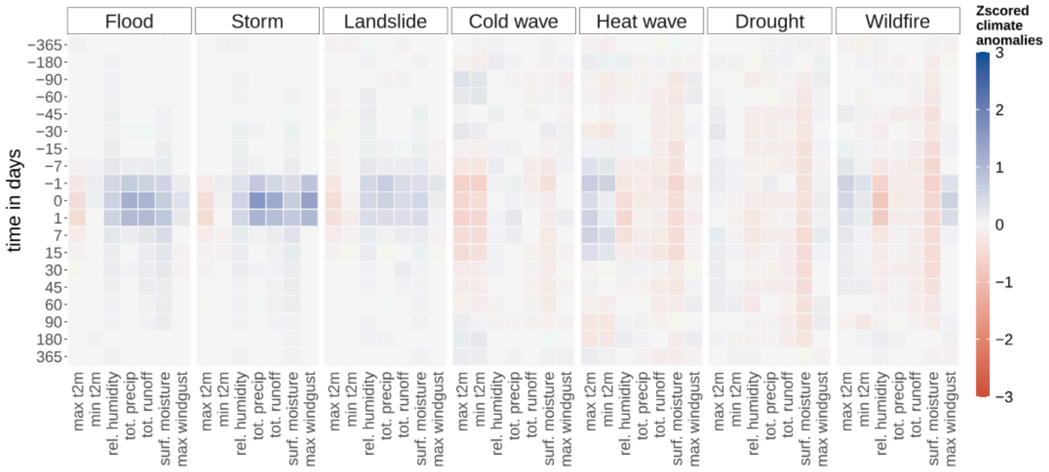

Fig. S17: **Average climatological anomalies of the different hazard types**. Average climate anomalies per hazard type (panel) at selected dates between one year before and after the reported start of the events. The Composite climatological anomalies were calculated using the superposed epoch analysis (reference in methods 4.2.1). These anomalies show the temporal evolution of the averaged daily anomalies. All climate variables (x-axis) are scaled to zero mean and unit variance. Long names of the climate variables: maximum temperature, minimum temperature, relative humidity, total precipitation, surface moisture, total runoff and maximum wind gust.

These patterns appear clearly even with the fixed window, demonstrating that SEA successfully reveals hazard-specific temporal profiles without dilution or interference.

Varying the SEA window (shorter or longer) does not change the qualitative behavior of the composites:

- Peaks for storms and floods remain sharply localized around day 0.
- Long-lived anomalies for droughts and wildfires remain visible months before and after the reported event dates.

The SEA figures remain consistent under different window lengths and continue to depict the expected hazard-specific temporal structure.

To avoid possible confusion, we have added the following clarifications to the Methods section:

L542-545: Using a fixed multi-year window is standard practice in SEA applications in climate impact research, as it provides a consistent temporal frame for averaging heterogeneous events and allows comparison across hazard types.

L549-552: The SEA is used only for descriptive context and does not contribute to the event-level anomalies used in the analyses. Hazard anomalies used in the analysis are instead computed with a ±3-day window around the reported event start date of each hazard (Section 4.2.2).

4.The source of the sHDI data provided in the paper is Reference 27, and the time series of the sHDI data given in this reference seems to be 1990–2017, rather than 1990–2020 as claimed in this study. Please confirm whether the time series are consistent; if not, please explain how the missing part is supplemented.

We thank the reviewer for this comment.

Reference 27 documents the methodology used to construct the subnational Human Development Index (sHDI), but does not correspond to the most recent data release. The Global Data Lab (GDL) continues to update the dataset annually using the same methodology and provides sHDI estimates beyond 2017. We cite reference 27 as recommended by the Global Data Lab.

In our study, we used the 7th update (release year: 2023) which provides annual sHDI estimates and their components (education, health, income) for administrative level-1 regions for the full period 1990–2020. This ensures that the temporal coverage of our analysis is consistent with the study period.

In a small number of cases with short missing sequences, we applied a conservative gap-filling rule (linear interpolation for gaps ≤ 5 years; longer gaps excluded), as indicated in methods 4.6. This affects only a small fraction of observations and does not change the temporal trends.

We have introduced the following changes in the methods section:

L620-625: We use the 2023 release (7th update) of the Global Data Lab (GDL) Subnational Human Development Index, which provides annual sHDI estimates and their components (education, health, income) for administrative level-1 regions from 1990 to 2020.

L631-634: In a small number of cases involving short missing sequences, we applied a conservative gap-filling procedure using linear interpolation for up to 5 consecutive years; gaps exceeding this limit were treated as missing, and the corresponding events were removed.

5.The current discussion section covers multiple topics. It is suggested to add subheadings to enhance readability and logical organization.

We thank the reviewer for this comment. We agree that improving the clarity and flow of the Discussion is important. However, Nature Communications does not permit subheadings within the Discussion section. To address the reviewer's concern while adhering to journal style, we carefully revised and edited the discussion by merging paragraphs with similar content, and by strengthening transitions between thematic blocks. These edits improve the logical progression of arguments and enhance readability without altering the substantive content of the discussion, and should guide the reader more effectively.

In doing so, we streamlined the Discussion into seven coherent paragraphs covering:

(1) summary of the main findings,
(2) the role of sHDI components,
(3) long-term vulnerability trends across hazard types,
(4) implications for future risk trajectories in low sHDI regions,
(5) data and methodological limitations,
(6) actionable insights for vulnerability reduction
(7) broader political and societal implications

We strengthened transitions between these paragraphs to guide the reader through the narrative more clearly. For example:

L392-394: Having established these global patterns, we next examine the underlying mechanisms that link human development to disaster impacts.

L404-405: Taken together, these component differences inform the vulnerability profiles of impacted regions.

L430-431: Moreover, tracking this evolution is challenging, as reliable data are often scarce in these areas.

L478-479: Despite these limitations, several actionable insights emerge regarding pathways to reduce disaster risk.

We merged the 2 limitations paragraphs: the one on sHDI and the one on disaster data, as they concern the same thematic issue—measurement constraints—and therefore read more coherently as a single integrated limitations section.

These edits preserve compliance with journal style while achieving the reviewer's objective of improved structure and logical progression.

We thank the reviewer for this helpful suggestion; the revised Discussion is now clearer, more concise, and more logically organized.

6.In Figure 1a, colors represent different sHDI levels, but do the shades of colors correspond to other variables? Please supplement the legend.

We thank the reviewer for this comment. In Figure 1a, the colors represent only the four sHDI development groups. No additional variables are encoded through shading or color intensity. We note that, for some hazard types, the concentration of points on the map may visually resemble a gradient due to overlap. To avoid ambiguity, we clarify this in the figure legend: "Colors indicate sHDI development groups. No additional shading or gradient encodes further variables; apparent density patterns result from point overlap."

Reviewer #2 (Remarks on code availability):

The code is appropriate and provides a README file.

We thank the reviewer for the positive assessment of the code availability and documentation.

Reviewer #3 (Remarks to the Author):

Thank you very much for the opportunity to review the revised/transferred. I understand that the authors defend the use of EM-DAT being the only global dataset with no additional analysis to explore the known areas of limitation. I am fine with this approach as long as the discussions of alternative datasets suggested (e.g. Desinvestar, satellite) etc are explored sufficiently in the main text as further avenues to validate the findings etc. Given the paper's interest to explore the socioeconomic vulnerabilities, I think a meaningful additional analysis can be done for those sub-set of countries for which data is known to be sparse. This was the spirit of my previous comments and the author chooses not to, which is fine. But the idea of scientific contribution is to advance our knowledge and not to continue defending the limited approach just because many other studies have done them.

We thank the reviewer for this constructive and clarifying comment. We agree that strengthening the contextualization of EM-DAT and acknowledging opportunities for complementary validation enhances the scientific contribution of the manuscript.

Our analysis focuses on global, cross-country patterns at the subnational level, and therefore requires harmonized, internationally comparable observations across thousands of disaster events and all major climate-related hazard categories. At present, EM-DAT remains the only global database that provides consistent reporting of human and economic disaster impacts across 150+ countries and multiple hazard types over multi-decadal time scales. Our use of EM-DAT is therefore not a defense of the status quo, but a methodological necessity for a unified global analysis.

That said, we fully agree with the reviewer that complementary impact databases -such as national level sources like DesInventar, or emerging satellite-based damage assessments products- offer valuable opportunities for validation and for improving coverage in regions with sparse EM-DAT reporting. However, integrating these sources into a global analysis of 7,061 events would require substantial harmonization across reporting systems, impact definitions, spatial referencing, and temporal consistency. This is, indeed, an important suggestion for the direction of future work.

In response to the reviewer's suggestion, we have expanded the Discussion to explicitly acknowledge both the limitations of relying solely on EM-DAT and the potential of additional datasets for future validation and refinement. The revised text now reads:

L473-477: Complementary impact datasets, including national disaster inventories and emerging satellite-based assessments, offer promising avenues for validation and improved coverage in regions with sparse reporting. Integrating and harmonizing such sources in future work could substantially enhance empirical assessments of disaster impacts.

This addition ensures that the manuscript explicitly acknowledges the limitations of relying solely on EM-DAT and clarifies that our choice reflects the requirements of global comparability. We thank the reviewer for highlighting this issue; their comment helped us substantially strengthen the contextual framing of the study.

In terms of terminology, I appreciate the author's clarification on hazard intensity. The manuscript is now clear for the general scientific audience who will be following a more conventional framing of hazard, exposure vulnerability as adopted by IPCC. On terminology, as much as possible, please refrain from including in-precise use of terms such as 'humanitarian disasters'. 'affected population (requiring humanitarian assistance)'. Please align as much to IPCC or PDNA/DALA, or other internationally accepted glossaries such as those of UNDRR. The affected population are simply affected population, if they require humanitarian assistance as such, for example, is a function of another dimension such as their local coping capacities. As the focus of this paper is on social vulnerability of disasters, for which well-established strands of literature exist. Please pay due attention to those and avoid introducing new terminology/concept that will confuse the readers.

We thank the reviewer for this important comment and fully agree that terminological consistency with internationally recognized glossaries is essential for clarity and conceptual precision.

In response, we revised the manuscript to align terminology with the IPCC and UNDRR risk framework, ensuring consistent use of the core concepts hazard, exposure, vulnerability, and impacts, following established definitions.

To avoid ambiguity, we refrained from using the term “severity” as a standalone concept. Where it previously referred to physical characteristics of climate-related processes, it was clarified as hazard intensity; where it referred to consequences for populations or economic assets, it was clarified as impact magnitude or losses, depending on context. This ensures conceptual precision without changing the interpretation of the results.

We further removed non-standard or potentially misleading expressions such as “economic damage,” “mitigate disaster risk,” “humanitarian disasters”, “humanitarian impacts”, and now refer to “economic losses”, “manage disaster risk,” “climate-related disasters”, and “adverse impacts”. The main text and figures were updated accordingly.

Finally, we ensured consistent use of the term “hazard” when considering the physical climate-related process of the event, while reserving references to “disaster” for events with realized human and economic outcomes. These revisions improve clarity and ensure alignment with established international usage without introducing new concepts.

Reviewer #3 (Remarks on code availability):

N.A.

REVIEWERS' COMMENTS

Please find below our point by point responses to the reviewer comments. Our replies are provided in blue after each comment.

Reviewer #2 (Remarks to the Author):

The reviewers have enhanced the rigour of the research and the clarity of its presentation. The findings demonstrate clear innovation and academic contribution. Prior to publication, the following details require refinement:

1.In the caption of Figure 1a, it is suggested to clarify that "point density reflects only the number of events, and the apparent clustering results from spatial overlap of points, rather than representing any additional variable."

We thank the reviewer for this comment. We updated the caption of figure 1a as follows: Climate-related disasters and intra-country deviation of human development in impacted regions. (a) Locations of climate-related disasters from the Emergency Events Database (EM-DAT) colored by subnational Human Development Index (sHDI) group, shown separately for each disaster type between 1990 and 2020 (total n=7061; per-type counts indicated in each panel). Disaster counts by latitude are shown on the right of each map. Point density reflects only the number of events, and the apparent clustering results from spatial overlap of points, rather than representing any additional variable.

2.Regarding the sentence "To limit spatial uncertainty, we excluded events with a total area larger than one million square km," it is advisable to provide literature support for the selection of this threshold.

We thank the reviewer for this comment. We chose this threshold as a compromise between geographic accuracy and accounting for large scale events. We are aware that the subnational reporting of EM-DAT is based on administrative units from where impacts are reported and not the hazard footprints (Teber et al., 2025). Since even at the subnational administrative level the reported hazard does not necessarily cover the entire administrative region (Rosvold & Buhaug, 2021), aggregating population and GDP across the full region introduces uncertainty that grows with area. To reduce this uncertainty, we set the upper threshold to one million square km. However, the bulk of our sample is composed of much smaller regions as shown in Fig. R1. This approach helps ensure that the events in our sample are of a scale where the underlying hazard-exposure dynamics can be analyzed with greater homogeneity and reduced uncertainty. Despite this inherent spatial uncertainty, analyzing events at the subnational administrative level represents an improvement over national-level approaches, which cannot capture within-country heterogeneity.

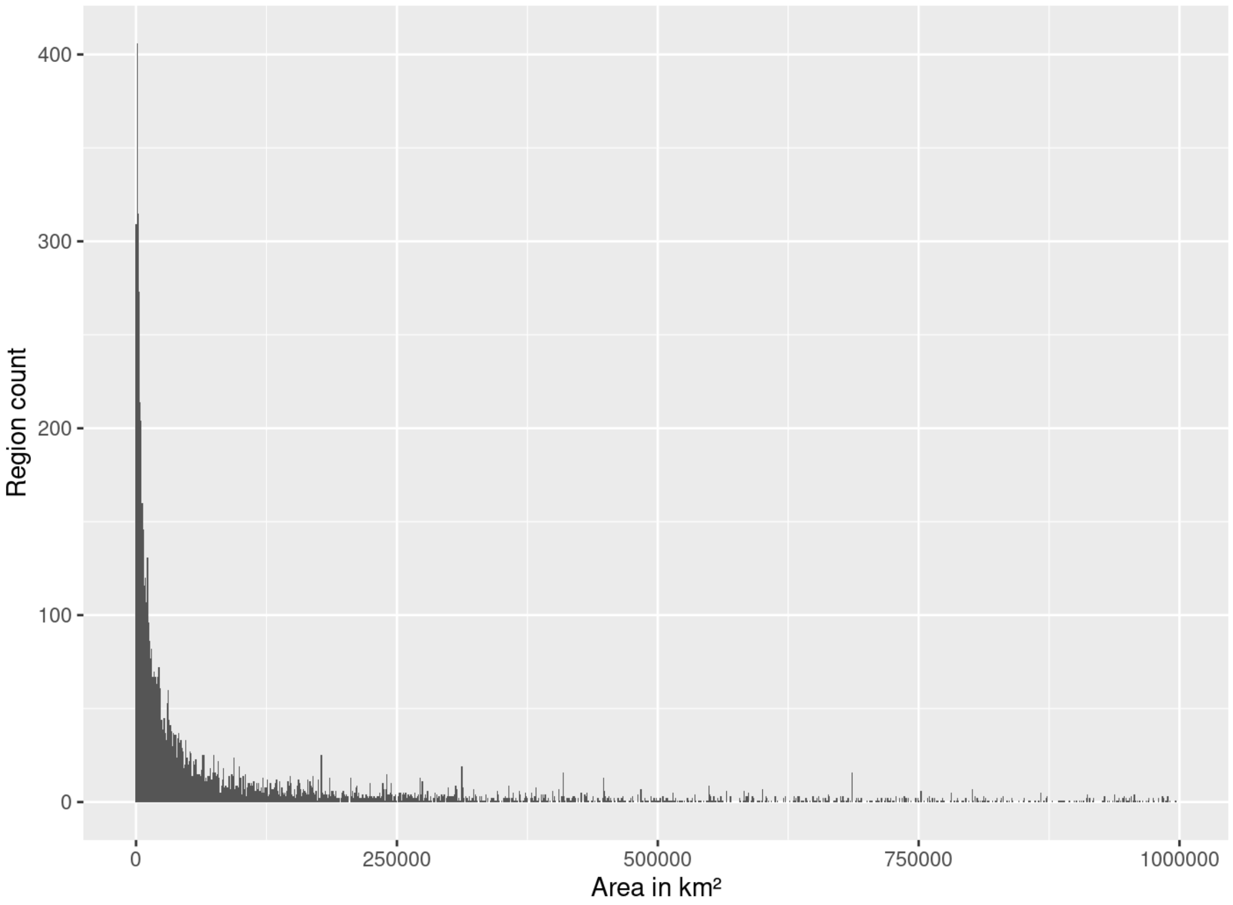

Fig. R1 Distribution of impacted region areas in the analyzed sample (km²). The vast majority of events involve regions well below the 1,000,000 km² threshold, with a median area of 27,004 km² and a 75th percentile of 125,003 km².

To ensure more clarity, we revise the Geocoding of disaster events section in the Methods as follows:

L463 - 468: To limit spatial uncertainty, we excluded events with a total area larger than one million square km. EM-DAT reports impacts at the subnational administrative unit level rather than the hazard footprint (Teber et al., 2025), and subnational administrative regions are not necessarily fully impacted by a hazard (Rosvold & Buhaug, 2021), meaning that aggregation uncertainty grows with area. The vast majority of events in our sample fall well below this threshold, with a median impacted area of 27,004 km² and a 75th percentile of 125,003 km².
